# Factors Associated With the Acceptance of an eHealth App for Electronic Health Record Sharing System: Population-Based Study

**DOI:** 10.2196/40370

**Published:** 2022-12-12

**Authors:** Junjie Huang, Wing Sze Pang, Yuet Yan Wong, Fung Yu Mak, Florence S W Chan, Clement S K Cheung, Wing Nam Wong, Ngai Tseung Cheung, Martin C S Wong

**Affiliations:** 1 The Jockey Club School of Public Health and Primary Care Faculty of Medicine Chinese University of Hong Kong Sha Tin Hong Kong; 2 Centre for Health Education and Health Promotion, Faculty of Medicine Chinese University of Hong Kong Sha Tin Hong Kong; 3 Information Technology and Health Informatics Division Hospital Authority Kowloon Hong Kong

**Keywords:** digital health, eHealth, electronic health record, system, mobile app, app, public, private, community, caregiver, awareness, perception, improvement, utility, technology, model, health information

## Abstract

**Background:**

In the second stage of the Electronic Health Record Sharing System (eHRSS) development, a mobile app (eHealth app) was launched to further enhance collaborative care among the public sector, the private sector, the community, and the caregivers.

**Objective:**

This study aims to investigate the factors associated with the downloading and utilization of the app, as well as the awareness, perception, and future improvement of the app.

**Methods:**

We collected 2110 surveys; respondents were stratified into 3 groups according to their status of enrollment in the eHRSS. The primary outcome measure was the downloading and acceptance of the eHealth app. We collected the data on social economics factors, variables of the Technology Acceptance Model and Theory of Planned Behavior. Any factors identified as significant in the univariate analysis (*P*<.20) will be included in a subsequent multivariable regression analysis model. All *P* values ≤.05 will be considered statistically significant in multiple logistic regression analysis. The structural equation modeling was performed to identify interactions among the variables.

**Results:**

The respondents had an overall high satisfaction rate and a positive attitude toward continuing to adopt and recommend the app. However, the satisfaction rate among respondents who have downloaded but not adopted the app was relatively lower, and few of them perceived that the downloading and acceptance processes are difficult. A high proportion of current users expressed a positive attitude about continuing to adopt and recommend the app to friends, colleagues, and family members. The behavioral intention strongly predicted the acceptance of the eHealth app (β=.89; *P*<.001). Attitude (β=.30; *P*<.001) and perceived norm; β=.37; *P*<.001) played important roles in determining behavioral intention, which could predict the downloading and acceptance of the eHealth app (β=.14; *P*<.001).

**Conclusions:**

Despite the high satisfaction rate among the respondents, privacy concerns and perceived difficulties in adopting the app were the major challenges of promoting eHealth. Further promotion could be made through doctors and publicity. For future improvement, comprehensive health records and tailored health information should be included.

## Introduction

In Hong Kong, a substantial proportion of hospital services is provided by the public sector (90% of all in-patient bed-days) and up to 70% of the outpatient services are offered by the private sector [1]. In view of the dual-track health care system, the Electronic Health Record Sharing System (eHRSS) was developed by the Hospital Authority (HA) to facilitate the information flow between the public and private sectors. It was launched in March 2016 [2] as an electronic platform to provide accurate and quick retrieval of clinical details, such as patient demographics, clinical information, and prescription profiles. The benefits of eHRSS are facilitation of patient communication, improvement of patient care continuity, accuracy of drug prescription, and enablement of holistic management [3]. Stage 2 development of the eHRSS started in July 2017, which further expanded the benefits to the relevant stakeholders and users. These include the broadening of the scope of sharable data by the system; provision of patients’ choice over data sharing scope; and their access to some of the data in the eHRSS [4]. As of May 2022, over 5.5 million citizens, 50,000 health care professionals, all the 13 private hospitals, and over 2400 health care professionals working in the private sectors have enrolled in the eHRSS [5].

In stage 2 development, a mobile app, an “eHealth app,” was launched in January 2021 [6]. It facilitates users to access their integrated health records and manage own health. Our team has previously evaluated the perceptions of and factors associated with the acceptance of the eHRSS in 2018 among 2000 patients in Hong Kong [7]. More than 70% (707/1000, 70.70%) of the patients were satisfied with the overall performance of the eHRSS. The expansion of sharable scope in the eHRSS (32/124, 25.8%) and allowing patients to access their medical records (30/124, 24.2%) were considered as the features to be developed in the future development of the eHRSS by the enrollees. This is one of the survey findings that provides support for the second stage of the eHRSS, where the users may access their health records and other health information via the utilization of an eHealth app. This mobile app further enhances collaborative care among the public sector, private sector, community, and caregivers. Importantly, citizens could be empowered in self-health management and disease prevention by recording health data within the app. It further empowers citizens’ self-care ability by involving family members and other stakeholders to understand their current health status.

Across the world, similar mobile health apps were developed for people to upload and view health records, manage personal health care activities, share clinical information with doctors, and improve public health. Apps such as “Capzule PHR,” “Health and Family,” and “Health Notes” allow patients to view and get access to their medical information and record their data at any time and any place through the internet or by offline access [8]. The government of various countries is promoting electronic medical health records. For example, “MIDATA” is the UK government program with the goal of providing consumers a better control over their data [9]. The Mi Health App was developed accordingly to record health data and support long-term health management [10]. In 2019, the Korean government initiated the “MyData” program, which aims to give citizens increased access to personal data through mobile phones. In the medical field, it enables the public to manage their medical record [11]. The My HealthWay app was launched in 2021 by the Korean Ministry of Health and Welfare to integrate scattered medical data [12].

To further promote quality and efficiency, as well as to recommend the future development of the mobile (eHealth) app, perceptions and views from users are required to inform a more system-friendly design. The objectives of this project are to evaluate the factors associated with the downloading and utilization of the eHealth app; to examine the awareness, use, and acceptability of the mobile eHealth app; to explore whether eHealth app use may be associated with the joining of the eHRSS; the reasons for nonuse among those who joined the eHRSS; the extent to which the app improves user experience and influences health service utilization; and to recommend a potential room for improvement of the eHealth apps.

## Methods

### Sampling Frame and Recruitment

A self-administered questionnaire was adopted in this study. Prospective study participants were based on a list of patients provided by the HA. A simple random sampling methodology was mainly used. Over 5.5 million existing eHRSS users were included in the population, and computer-generated numbers were listed correspondingly for participant recruitment. An invitational SMS was first sent by the HA to existing eHRSS users. This served to alert the participants that they would receive a subsequent survey invitation by Chinese University of Hong Kong via SMS [13]. Then research teams at the Chinese University of Hong Kong sent messages to those people who had received an invitation from the HA through a bulk SMS sending platform (MD SMS by Media Digital Technologies Corporation Limited). Supplemented by a convenience sampling methodology, the online survey link was shared on the website of the HA, eHealth Facebook and Instagram page, eHealth app, eHealth website so that both health care recipients and non–health care recipients could access the questionnaires. The overall response rate was 66.71% (3026/4536).

### Survey Instruments

Survey items focused on the awareness, use, and acceptability of the eHealth app; the association between the use of the eHealth app and the joining of the eHRSS; the reasons why some users did not use the eHealth app after joining the eHRSS; the extent to which the eHealth app improved user experience, modified health service access, and health management; and recommendations for possible improvement of the eHealth app. The surveys were designed by an academic physician with relevant experience in projects related to the eHRSS, and extensive expertise in both clinical and public health research studies. The questionnaire draft was face-validated by a panel of epidemiologists, biostatisticians, and professionals in the field of health care policy, public health, and primary care. It was subsequently pilot tested for feasibility and item comprehensiveness among 20 people. The completion rate was 65% (13/20), and the average response time was 7 minutes and 40 seconds (Multimedia Appendix 1).

The surveys were available in both Chinese and English versions. All surveys were anonymous, and written consent was provided by the participants at the start of the questionnaire. The study participants were informed that all information presented would be in the form of aggregated data that could not identify any individuals.

### Ethics Approval

This study was approved by the Survey and Behavioral Research Ethics Committee of the Chinese University of Hong Kong (approval number SBRE-21-0184).

### Statistical Analysis

All surveys were checked for their completeness and the presence of participant consent. All data entry and analysis were conducted using SPSS version 26.0 (IBM, Inc.). As part of quality control, at least 20% (422/2110) of all surveys were randomly checked for the validity, quality, and accuracy. All items in the survey were analyzed as stratified according to the status of enrollment. The primary outcome measure was the downloading and acceptance of the eHealth app. We tested for the presence of statistical association by identifying potential associated factors using univariate and multivariate regression analyses. We included age, gender, educational level, job status, monthly household income, the types of mobile phone operating systems currently in use, the eHRSS enrollment status, perceived enablers of acceptance, and perceived barriers of the eHealth app use. Any factors identified as significant in univariate analysis (*P*<.20) will be included in a subsequent multivariable regression analysis model. All *P*values ≤.05 will be considered statistically significant in the multiple logistic regression analysis. Assuming the proportion of the primary outcomes was 50%, which would provide the largest sample size, a total of 2110 surveys would result in precision of approximately 2.2%. In addition, we performed structural equation modeling to identify interactions among the variables.

### Health Behavioral Models

To investigate the factors that could predict downloading and acceptance of the eHealth app, we used 2 internationally recognized models that have been widely adopted to examine the use of new technologies. These were the Technology Acceptance Model (TAM), which was first developed by Fred D Davis, Richard P Bagozzi, and Paul R Warshaw [14]. It is an adaptation of the Theory of Reasoned Action (TRA) to the discipline of information systems. The TAM hypothesizes that perceived usefulness and perceived ease of use could influence an individual’s intention to use an information system [15]. The meditator of actual acceptance of the system is the intention to use. The model also considered perceived ease of use as a direct determinant of perceived usefulness. The TAM has been simplified by omitting the construct pertinent to attitude, as used in the TRA model. In the survey, perceived usefulness has been assessed using a series of questions related to the convenience and the benefit of using the app. To measure the ease of use, respondents have been asked about their experience in the downloading and acceptance processes, whether the app is easy to download, easy to find function, and contains the health information they want. For perceived barriers, respondents were asked about factors preventing them from downloading or adopting the app, such as doctors do not recommend or participate and concerns about personal privacy (Multimedia Appendix 2).

Furthermore, we employed the Theory of Planned Behavior (TPB), a commonly used psychological theory that links people’s beliefs and behaviors [16]. The underpinning theory identified 3 core predictors, namely, attitude (A1-4), subjective norms (SN1-3), and perceived behavioral control (BI1-2) as modifiers of intention. Items from these 3 constructs, for example, suggestions from people who influence users’ behavior, were recoded into the questionnaire as measurement (Multimedia Appendix 3) [17,18]. A 5-point Likert scale (strongly disagree, disagree, neutral, agree, and strongly agree) and a 2-point Likert scale (yes and no) were used in the survey design. Survey questions related to the acceptance and use of the app were designed according to the components of the TAM and TPB models. In our survey, attitude was the measurement of enabling factors, and the subjective norm referred to how the respondents viewed the idea of other people’s perceptions about the app, including the recommendation from doctors, friends, and family members. The specific questions related to attitude and subjective norm are “Do you agree with the following reasons that can increase your motivation to continuously use/start to use the eHealth app” and “Do you agree with the following reasons that hinder your motivation to continuously use/start to use the eHealth App” (Multimedia Appendix 4). The theory hypothesized that behavioral intention is the most antecedent influencer of behavior. In the current structural equation modeling, we included the following additional variables into the TAM: age, gender, educational level, occupation, types of mobile phone operating systems, and enrollment status of the eHRSS. All *P* values ≤.05 were regarded as statistically significant.

## Results

### Participant Characteristics

A total of 2110 completed surveys were collected (Table 1). Overall, there were more male than female respondents (1184/2110, 56.11%, vs 926/2110, 43.89%). Among the study participants, 46.16% (974/2110) were aged between 41 and 60, while 39.72% (838/2110) were aged above 60. Over one-half of the respondents attained secondary educational level (1118/2107, 53.06%). Nearly half of the respondents had full-time or part-time jobs (999/2024, 49.36%). For income level, the highest proportion of monthly household income was HK $10,000-19,999 (1HK $=US $0.12; 458/2110, 26.44%).

**Table 1:** Participant characteristics (N=2110).

Characteristics		Values, n (%)
**Age (years)**		
	16-30	136 (6.45)
	31-40	162 (7.68)
	41-50	343 (16.26)
	51-60	631 (29.91)
	61-70	636 (30.14)
	>70	202 (9.57)
**Gender**		
	Male	1184 (56.11)
	Female	926 (43.89)
**Educational level (n=2107)**		
	Primary or below	150 (7.12)
	Secondary	1118 (53.06)
	Tertiary or above	839 (39.82)
	Other	3 (not counted)^a^
**Job status (n=2024)**		
	Employed (Full-time/part-time)	999 (49.36)
	Unemployed	100 (4.94)
	Retired	695 (34.34)
	Housewives	138 (6.82)
	Students	53 (2.62)
	Others	39 (1.93)
	Refuse to answer	86 (not counted)^a^
**Monthly household income (HK $; n=1732)^b^**		
	<10,000	373 (21.54)
	10,000-19,999	458 (26.44)
	20,000-29,999	335 (19.34)
	30,000-39,999	154 (8.89)
	40,000-59,999	180 (10.39)
	≥60,000	232 (13.39)
	Refuse to answer	378 (not counted)^a^
**Phone currently in use**		
	Apple iOS	700 (33.18)
	Android	1110 (52.61)
	Huawei	174 (8.25)
	Others	126 (5.97)

^a^As these options are out of the original categories, the answers were “not counted” and thus not used in the analysis.

^b^1HK $=US $0.12.

Participants were classified into several groups according to downloading and acceptance of the eHealth app (Multimedia Appendix 5). A total of 1242 respondents have enrolled in the eHRSS, downloaded, and adopted the eHealth app (group 1). There were 275 participants who have enrolled in the eHRSS, downloaded the eHealth app, but did not adopt the app (group 2). The third group included 203 respondents that have enrolled in the eHRSS, but have neither downloaded nor adopted the app (group 3). In the following paragraphs, the findings were stratified according to these 3 groups of respondents.

The COVID-19 vaccination program (649/2110, 30.76%), medical doctors (647/2110, 30.66%), publicity (posters, pamphlets, television, outdoor advertisements; 533/2110, 25.26%), and friends or family members (388/2110, 18.39%) were the 4 major sources of information about the eHealth app among respondents (Multimedia Appendix 6). We did not observe a distinct difference in the distribution of sources among the 3 groups.

### Perceived Enablers and Barriers of the App

In group 1, the majority of participants agreed that the app can show their accurate vaccination records (1118/1242, 90.02%) and other health records (1081/1242, 87.04%). They also expressed that the app provides useful administrative functions, including giving consent to health care providers for sharing their data (1044/1242, 84.06%), easier management of eHealth accounts (1005/1242, 80.92%), and empowerment of their family members and own health (940/1242, 75.68%). A similar result was also noted in the other 2 groups (Tables 2 and 3).

Among the study participants in group 1 (Tables 4 and 5), the major barrier was that their physicians had not joined the eHealth app (505/1028, 49.12%) and that their doctors did not mention, recommend, or think it is necessary to use the eHealth app (417/1078, 38.68%). Respondents in groups 2 and 3 perceived that the downloading procedure is complicated (172/382, 45%) and were concerned about their personal information and privacy (243/461, 52.7%), respectively.

**Table 2:** Perceived enablers of downloading the eHealth app.

Enablers of downloading	Downloaded and used eHealth app (n=1242)	Downloaded but not used eHealth app (n=399)	Not having downloaded and used eHealth app (n=469)
	Strongly agree or agree, n (%)	Strongly agree or agree, n (%)	Strongly agree or agree, n (%)
It is convenient to get information about different government-subsidized medical programs	920 (74.07)	293 (73.43)	332 (70.79)
I can view my accurate health records	1081 (87.04)	309 (77.44)	380 (81.02)
I can manage my eHealth account easily (eg, update the communication means)	1005 (80.92)	281 (70.43)	359 (76.55)
I can give sharing consents to health care providers easily so that they can view my health records	1044 (84.06)	307 (76.94)	378 (80.60)
I can find the health care providers and doctors that are participating in different health programs with ease	899 (72.38)	269 (67.42)	368 (78.46)
I can check the remaining balance and record of the Elderly Health Care Voucher Scheme	904 (72.79)	270 (67.67)	371 (79.10)
I can show the vaccination record/QR code	1118 (90.02)	321 (80.45)	383 (81.66)
It helps to manage my health and my families’ health	940 (75.68)	274 (68.67)	367 (78.25)
My friend recommended me to use the “eHealth” app	691 (55.64)	202 (50.63)	244 (52.03)
My family recommended me to use the “eHealth” app	777 (62.56)	225 (56.39)	282 (60.13)
My doctor recommended me to use the “eHealth” app	797 (64.17)	240 (60.15)	312 (66.52)
Government’s advertisement of the “eHealth” app	730 (58.78)	216 (54.14)	271 (57.78)
I can get souvenirs	466 (37.52)	148 (37.09)	201 (42.86)

**Table 3:** Perceived enablers of acceptance of the eHealth app.

Enablers of acceptance	Downloaded and used eHealth app (n=1242)				Downloaded but not used eHealth app (n=399)				Not having downloaded and used eHealth app (n=469)			
	n	Mean (SD)	95% CI	n		Mean (SD)	95% CI	n		Mean (SD)	95% CI	
It is convenient to get information about different government-subsidized medical programs	920	3.84 (0.78)	3.80-3.89	293		3.75 (0.85)	3.66-3.83	332		3.76 (0.75)	3.69-3.82	
I can view my accurate health records	1081	4.15 (0.79)	4.11-4.20	309		3.87 (0.87)	3.78-3.96	380		3.96 (0.71)	3.89-4.02	
I can manage my eHealth account easily (eg, update the communication means)	1005	3.99 (0.73)	3.95-4.03	281		3.70 (0.86)	3.62-3.79	359		3.86 (0.71)	3.79-3.92	
I can give sharing consents to health care providers easily so that they can view my health records	1044	4.07 (0.73)	4.03-4.11	307		3.84 (0.85)	3.75-3.92	378		3.92 (0.71)	3.86-3.99	
I can find the health care providers and doctors that are participating different health programs with ease	899	3.86 (0.75)	3.82-3.90	269		3.68 (0.80)	3.61-3.76	368		3.89 (0.69)	3.82-3.95	
I can check the remaining balance and record of the Elderly Health Care Voucher Scheme	904	3.90 (0.81)	3.86-3.95	270		3.71 (0.86)	3.63-3.80	371		3.88 (0.72)	3.82-3.95	
I can show the vaccination record/QR code	1118	4.22 (0.72)	4.18-4.26	321		3.95 (0.86)	3.86-4.03	383		4.00 (0.73)	3.93-4.06	
It helps to manage my health and my families’ health	940	3.93 (0.79)	3.89-3.98	274		3.73 (0.89)	3.64-3.82	367		3.89 (0.72)	3.83-3.96	
My friend recommended me to use the “eHealth” app	691	3.55 (0.91)	3.50-3.60	202		3.41 (0.94)	3.32-3.50	244		3.44 (0.86)	3.37-3.52	
My family recommended me to use the “eHealth” app	777	3.68 (0.89)	3.63-3.73	225		3.5 (0.95)	3.40-3.59	282		3.56 (0.87)	3.48-3.64	
My doctor recommended me to use the “eHealth” app	797	3.7 (0.88)	3.65-3.75	240		3.58 (0.88)	3.49-3.67	312		3.71 (0.77)	3.64-3.78	
Government’s advertisement of the “eHealth” app	730	3.61 (0.88)	3.56-3.66	216		3.49 (0.92)	3.40-3.58	271		3.52 (0.86)	3.44-3.59	
m. I can get souvenirs	466	3.21 (1.08)	3.15-3.27	148		3.15 (1.05)	3.05-3.25	201		3.24 (0.99)	3.15-3.33	

**Table 4:** Perceived barriers to downloading of the eHealth app.

Barrier	Downloaded and used the eHealth app (n=1028-1222)	Downloaded but not used the eHealth app (n=301-391)	Not having downloaded and used the eHealth app (n=365-461)
	Strongly agree or agree, n (%)	Strongly agree or agree, n (%)	Strongly agree or agree, n (%)
One’s physician has not joined	505/1028 (49.12)	133/310 (42.90)	151/365 (41.37)
Only see 1 doctor who is familiar with my health records	392/1092 (35.90)	144/347 (41.50)	181/425 (42.59)
No sickness	295/1157 (25.50)	97/358 (27.09)	156/441 (35.37)
Concerned about personal information and privacy	408/1222 (33.39)	168/388 (43.30)	243/461 (52.71)
My doctor did not mention about/recommend/think it is necessary to use the “eHealth” app	417/1078 (38.68)	136/333 (40.84)	183/403 (45.41)
I do not know how to use a smartphone/mobile app	203/1167 (17.40)	94/372 (25.27)	119/441 (26.98)
Not willing for others to read one’s own health records	372/1216 (30.59)	161/391 (41.18)	209/455 (45.93)
Uncertain about the benefits of the eHealth app	266/1198 (22.20)	134/374 (35.83)	172/437 (39.36)
Complicated downloading procedures	321/1216 (26.40)	172/382 (45.03)	173/423 (40.90)

**Table 5:** Perceived barriers to acceptance of the eHealth app.

Barrier	Downloaded and used the eHealth app (n=1242)			Downloaded but not used the eHealth app (n=399)				Not having downloaded and used the eHealth app (n=469)		
	n	Mean (SD)	95% CI	n	Mean (SD)	95% CI	n		Mean (SD)	95% CI
One’s physician has not joined	505	3.30 (1.08)	3.23-3.37	133	3.30 (0.92)	3.18-3.41	151		3.16 (0.95)	3.05-3.26
Only see 1 doctor who is familiar with my health records	392	3.03 (1.02)	2.96-3.09	144	3.09 (0.93)	2.97-3.2	181		3.12 (0.92)	3.02-3.23
No sickness	295	2.73 (1.02)	2.66-2.80	97	2.83 (0.95)	2.71-2.95	156		2.97 (0.97)	2.86-3.08
Concerned about personal information and privacy	408	2.94 (1.14)	2.86-3.02	168	3.09 (1.05)	2.96-3.22	243		3.46 (1.06)	3.34-3.58
My doctor did not mention about/recommend/think it is necessary to use the “eHealth” app	417	3.11 (0.98)	3.05-3.18	136	3.22 (0.84)	3.12-3.33	183		3.23 (0.86)	3.14-3.33
I do not know how to use a smartphone/mobile app	203	2.43 (1.11)	2.36-2.51	94	2.70 (1.07)	2.57-2.83	119		2.78 (1.00)	2.67-2.89
Not willing for others to read one’s own health records	372	2.89 (1.09)	2.82-2.96	161	3.08 (0.99)	2.95-3.2	209		3.28 (1.00)	3.17-3.39
Uncertain about the benefits of the eHealth app	266	2.70 (1.04)	2.63-2.77	134	3.06 (0.95)	2.95-3.18	172		3.15 (0.93)	3.05-3.26
Complicated downloading procedures	321	2.79 (1.05)	2.73-2.86	172	3.20 (1.01)	3.07-3.32	173		3.23 (0.88)	3.13-3.33

### Perception of Processes of Acceptance of the App

The proportion of participants in group 1 who were positive about the downloading and acceptance processes was in general higher than those in group 2. Most respondents in group 1 were satisfied with the downloading processes (908/1242, 73.11%; Multimedia Appendix 7). However, the proportion of group 2 participants expressing satisfaction about the downloading process was lower (239/399, 59.90%). Regarding the acceptance process, respondents in group 1 were satisfied with the app’s user experience and interface. They agreed that the fonts and size of the words were easy to read (947/1242, 76.25%), that the icon and tables were easy to understand (880/1242, 70.85%), and that the app was easy to use overall (869/1242, 69.97%). Among respondents in group 2, 60.6% (242/399) agreed that the fonts and size of the words were easy to read, and nearly half of them agreed that the icons and tables were easy to understand (190/399, 47.6%).

### Applicability and Perception of the App

In terms of applicability, vaccine records (1108/1242, 89.21%), appointment records (1055/1242, 84.94%), medication records (1015/1242, 81.72%), allergy records (924/1242, 74.40%), and health management (786/1242, 63.29%) were the top 5 useful functions among the users (Multimedia Appendix 8). These proportions were higher in group 1 than in group 2.

Turning to the perception of the app (Multimedia Appendix 9), a high percentage of group 1 respondents (ie, app users) were satisfied with the app overall (975/1242, 78.50%), agreed that it enhanced the experience of health services (962/1242, 77.46%), enhanced concerns about health information (926/1242, 74.56%), and enhanced management of health on their own (889/1242, 71.58%). Over 50% (211/399, 52.9%) agreed that the app improved the health of family members. Group 2 respondents (ie, nonusers) also reported a positive perception of the app, although the proportion agreeing with these items was lower.

### Expectations on the Future Development of the App

A high proportion of group 1 respondents, current users, expressed a positive attitude about continuing to adopt (1105/1242, 88.97%) and recommend the app to friends, colleagues, and family members (1024/1242, 82.45%; Multimedia Appendix 10). The proportion agreeing to continuously use and recommend among the nonusers in groups 2 and 3 was also high. Over 70% and 60% of the respondents in groups 2 (283/399, 70.9%, and 290/399, 72.7%) and 3 (320/469, 68.2%, and 304/469, 64.8%), respectively, expressed positive attitude toward future acceptance and recommendation of the app, respectively. Among all respondents, they expected to access more health records via the app, for example, the laboratory results (1707/2110, 80.90%) and the radiographic images (1484/2110, 70.33%), and to have customized health information, for example, age-specific health care recommendations (1378/2110, 65.31%) and tailored health tips (1121/2110, 53.13%). In group 1, the inclusion of the laboratory result was the most frequently cited item (1094/1242, 88.08%), followed by radiographic images (980/1242, 78.90%) and age-specific health care recommendations (843/1242, 67.87%). The results were similar compared with responses in groups 2 and 3.

### Factors Associated With Downloading and Acceptance

Respondents were more likely to download the app when they had joined the eHRSS (adjusted odds ratio [aOR] 9.2, 95% CI 6.35-13.32; *P*<.001); had attained secondary educational level (aOR 1.63, 95% CI 1.08- 2.46; *P*=.02); reported being able to view their accurate health records (aOR 1.41, 95% CI 1.02-1.95; *P*<.035); reported being able to show the vaccination records or QR codes (aOR 1.43, 95% CI 1.03-1.98; *P*=.031); and reported one’s physician had not joined the eHRSS (aOR 1.45, 95% CI 1.18-1.77; *P*<.001; Tables 6 and 7). Housewives (aOR 0.44, 95% CI 0.23-0.84; *P*=.013) and participants who were concerned about personal information and privacy (aOR 0.74, 95% CI 0.60-0.90; *P*=.003) were significantly less likely to download the eHealth app.

The independent factors associated with the acceptance of the eHealth app were similar to those associated with downloading, except that male participants (aOR 1.85, 95% CI 1.36-2.52; *P*<.001) were more likely to adopt, whereas individuals with primary educational level or below (aOR 0.49, 95% CI 0.25-0.94; *P*=.03) and study participants who were uncertain about the benefits of the eHealth app (aOR 0.80, 95% CI 0.66-0.96; *P*=.02) or perceived the downloading procedures as complicated (aOR 0.81, 95% CI 0.68-0.96; *P*=.01) were less likely to adopt (Tables 6 and 7).

**Table 6:** Factors associated with downloading and acceptance of the eHealth app.

Factor		Users, n (n=1159)	Downloading				Acceptance			
			Values, n (%)	aOR^a^ (95% CI)	*P* value	Values, n (%)		aOR (95% CI)	*P* value	
**Age (years)**					.63				.53	
	16-40	150	105 (70)	1 (reference)		82 (54.7)		1 (reference)		
	41-60	571	440 (77.1)	1.22 (0.70-2.11)	.48	347 (60.8)		1.31 (0.81-2.13)	.27	
	>60	438	361 (82.4)	1.40 (0.71-2.78)	.33	280 (63.9)		1.35 (0.75-2.43)	.32	
**Gender**										
	Male	680	553 (81.3)	1.19 (0.83-1.73)	.35	458 (67.4)		1.85 (1.36-2.52)	<.001	
	Female	479	353 (73.7)	1 (reference)		251 (52.4)		1 (reference)		
**Educational level**					.03				.04	
	Primary or below	73	50 (68.5)	0.91 (0.44-1.91)	.81	32 (43.8)		0.49 (0.25-0.94)	.03	
	Secondary	617	491 (79.6)	1.63 (1.08-2.46)	.02	373 (60.5)		1.05 (0.75-1.48)	.76	
	Tertiary or above	469	365 (77.8)	1 (reference)		304 (64.8)		1 (reference)		
**Job status**					.01				.48	
	Full-time/part-time	642	504 (78.5)	1 (reference)		404 (62.9)		1 (reference)		
	Unemployed	49	36 (73.5)	1.38 (0.59-3.21)	.46	26 (53.1)		1.21 (0.57-2.56)	.62	
	Retired	352	297 (84.4)	1.12 (0.67-1.88)	.67	230 (65.3)		0.89 (0.58-1.35)	.58	
	Housewives	74	45 (60.8)	0.44 (0.23-0.84)	.01	29 (39.2)		0.63 (0.34-1.15)	.13	
	Students	22	13 (59.1)	0.49 (0.15-1.57)	.23	11 (50)		0.82 (0.27-2.52)	.73	
	Others	20	11 (55)	0.27 (0.09-0.82)	.02	9 (45)		0.47 (0.16-1.38)	.17	
**Monthly household income (HK $)^b^**					.27				.82	
	<10,000	228	170 (74.6)	1 (reference)		118 (51.8)		1 (reference)		
	10,000-19,999	300	227 (75.7)	0.91 (0.55-1.51)	.72	177 (59)		1.20 (0.78-1.85)	.41	
	20,000-29,999	225	174 (77.3)	0.74 (0.43-1.29)	.29	141 (62.7)		1.07 (0.67-1.70)	.79	
	≥30,000	406	335 (82.5)	1.22 (0.71-2.08)	.47	273 (67.2)		1.18 (0.75-1.86)	.46	
**Phone currently in use**					.05				.19	
	Apple iOS	392	295 (75.3)	1 (reference)		239 (61)		1 (reference)		
	Android	615	501 (81.5)	1.22 (0.82-1.82)	.34	391 (63.6)		0.93 (0.67-1.31)	.69	
	Huawei	93	73 (78.5)	1.24 (0.63-2.46)	.53	54 (58.1)		0.83 (0.47-1.46)	.51	
	Others	59	37 (62.7)	0.46 (0.22-0.97)	.04	25 (42.4)		0.47 (0.24-0.94)	.03	
**Joining of eHRSS^c^**										
	Yes	924	807 (87.3)	9.20 (6.35-13.32)	<.001	665 (72)		9.77 (6.64-14.38)	<.001	
	No	235	99 (42.1)	1 (reference)		44 (18.7)		1 (reference)		

^a^aOR: adjusted odds ratio.

^b^1HK $=US $0.12.

^c^eHRSS: electronic Health Record Sharing System.

**Table 7:** Factors associated with perceived enablers and barriers of the eHealth app.

Factors			aOR^a^ (95% CI)		*P* value		aOR^a^ (95% CI)		*P* value
**Perceived enablers (score: 1 [strongly disagree] to 5 [strongly agree])**									
	It is convenient to get information about different government-subsidized medical programs	0.94 (0.69-1.28)		.70		0.95 (0.74-1.23)		.71	
	I can view my accurate health records	1.41 (1.02-1.95)		.04		1.40 (1.08-1.81)		.01	
	I can manage my eHealth account easily (eg, update the communication means)	0.82 (0.55-1.22)		.32		1.26 (0.90-1.75)		.18	
	I can give sharing consents to health care providers easily so that they can view my health records	1.14 (0.80-1.63)		.47		1.12 (0.84-1.50)		.44	
	I can find the health care providers and doctors who participated in different health programs with ease	0.49 (0.33-0.73)		.001		0.62 (0.45-0.85)		.003	
	I can check the remaining balance and record of the Elderly Health Care Voucher Scheme	0.99 (0.70-1.40)		.95		1.03 (0.78-1.37)		.82	
	I can show the vaccination record/QR code	1.43 (1.03-1.98)		.03		1.33 (1.02-1.75)		.03	
	It helps to manage my health and my families’ health	0.73 (0.51-1.06)		.09		0.76 (0.56-1.01)		.06	
	My friend recommended me to use the “eHealth” app	1.28 (0.88-1.86)		.20		0.98 (0.72-1.35)		.92	
	My family recommended me to use the “eHealth” app	1.10 (0.75-1.62)		.63		1.14 (0.82-1.59)		.42	
	My doctor recommended me to use the “eHealth” app	0.83 (0.60-1.13)		.23		0.85 (0.65-1.11)		.23	
	Government’s advertisement of the “eHealth” app	1.00 (0.76-1.32)		.97		1.01 (0.80-1.27)		.96	
	I can get souvenirs	1.14 (0.93-1.39)		.22		1.13 (0.95-1.34)		.17	
**Perceived barriers (score 1 [strongly disagree] to 5 [strongly agree], discard those answering “N/A”)**									
	One’s physician has not joined	1.45 (1.18-1.77)		<.001		1.18 (1.01-1.39)		.04	
	Only see 1 doctor who is familiar with my health records	1.01 (0.82-1.26)		.90		1.08 (0.90-1.29)		.42	
	No sickness	0.94 (0.76-1.16)		.58		0.97 (0.81-1.16)		.75	
	Concerned about personal information and privacy	0.74 (0.60-0.90)		.003		0.89 (0.75-1.05)		.16	
	My doctor did not mention about/recommend/think it is necessary to use the “eHealth” app	1.20 (0.95-1.51)		.12		1.05 (0.87-1.27)		.58	
	I do not know how to use a smartphone/mobile app	0.97 (0.81-1.17)		.77		1.04 (0.89-1.22)		.62	
	Not willing for others to read one’s own health records	1.05 (0.84-1.31)		.66		1.04 (0.87-1.24)		.68	
	Uncertain about the benefits of the eHealth app	0.81 (0.64-1.01)		.06		0.80 (0.66-0.96)		.02	
	Complicated downloading procedures	0.88 (0.71-1.08)		.23		0.81 (0.68-0.96)		.02	

^a^aOR: adjusted odds ratio.

### Findings From the Health Behavioral Models

**Figure 1:**
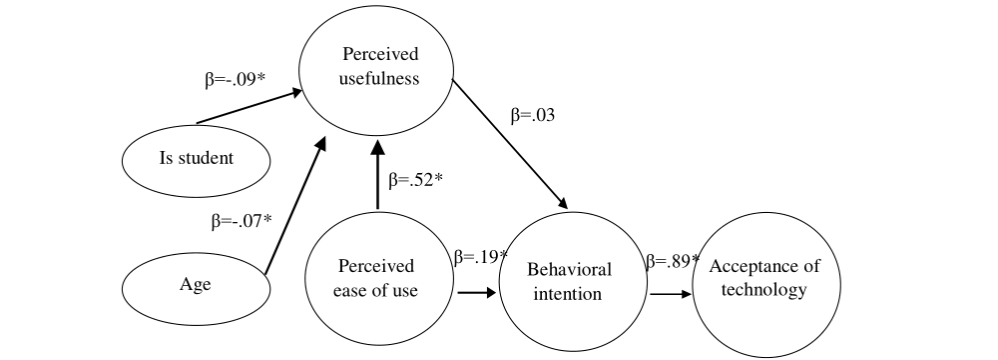
Factors predictive of downloading and acceptance of the eHealth app by the Technology Acceptance Model. *P<.05 (2-tailed).

In the TAM, perceived usefulness (β=.52; *P*<.001) and behavioral intention (β=.19; *P*<.001) were determined by perceived ease of use. The behavioral intention strongly predicted the acceptance of the eHealth app (β=.89; *P*<.001). Age (β=.07; *P*<.001) and whether the participant is a student or not (β=–0.09; *P*<.001) predicted the perceived usefulness. However, perceived usefulness did not significantly predict behavioral intention (β=.03; *P*=.32; Figure 1).

**Figure 2:**
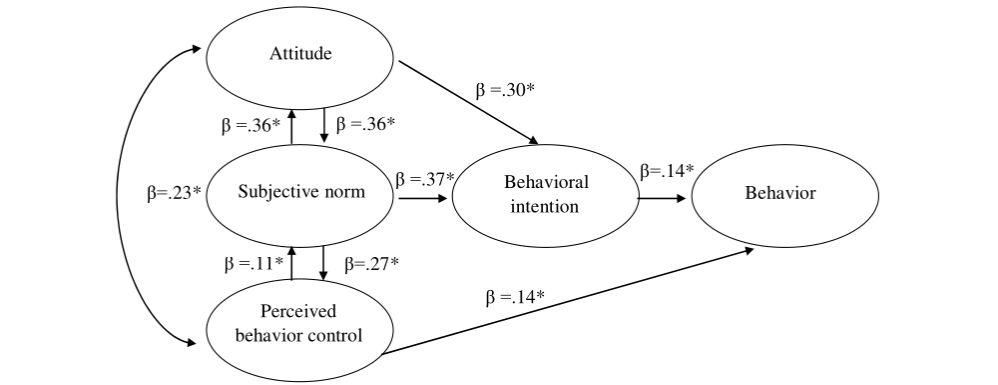
Factors predictive of downloading and acceptance of the eHealth app by the Theory of Planned Behavior. *P<.05 (2-tailed).

Turning to the TPB, attitude (β=.30; *P*<.001) and subjective norm (β=.37; *P*<.001) played important roles in determining behavioral intention, which could predict the downloading and acceptance of the eHealth app (β=.14; *P*<.001). The downloading and acceptance of the eHealth app could also be predicted by perceived behavior control (β=.14; *P*<.001). For the 3 core predictors, attitude was predicted by the subjective norm (β=.36; *P*<.001) and perceived behavior control (β=.23; *P*<.001). Subjective norm was predicted by attitude (β=.36; *P*<.001) and perceived behavior control (β=.11; *P*<.001). Perceived behavior control was predicted by attitude (β=.23; *P*<.001) and subjective norm (β=.27; *P*<.001; Figure 2).

## Discussion

### Principal Findings

Overall, the satisfaction rate among the respondents was high. The satisfaction rate among group 2 respondents was relatively lower, and few of them perceived the downloading process as complicated. The willingness to continue to use and recommend the app was strong among all respondents. The 3 major enablers of adopting the app were the viewing of health records, especially the vaccination record; managing their eHealth accounts and sharing consent; and managing their family members’ and their own health. However, respondents of the 3 groups had different perceived barriers. These include one’s physician had not joined the eHRSS or had not recommended the eHealth app to them, a complicated downloading process, and privacy concerns. Most of the respondents expected to access more health records in the app, such as laboratory results and radiographic images, and have more personalized health information and health tips based on their age groups and health condition.

### Limitations

This study has a few limitations. First, the survey was cross sectional, and so only the correlation could be measured instead of the causal relationship with the possibility of reverse causality. To corroborate the enablers and barriers, prospective longitudinal studies are required. In addition, face validity rather than construct validity was applied in the design of the questionnaire. The consistency reliability of the survey measurements has not yet been evaluated. Besides, some variables that could affect the downloading and acceptance of eHealth app may not be discussed in this study. Hence, there was a possibility of residual confounding. Finally, the study focused on acceptance of the app and examined individual factors affecting its use, which was based on a more individual level by using the TAM and TPB models in study design and analysis. Referring to Shachak et al’s [19] study on the complexity of the health information technology implementation, a more sociotechnical-level study that examines the complex and overall cyber-social system in which users, cultures, networks, technologies, and processes are involved should be conducted in the future.

### Comparison With Prior Work

eHealth app provides accurate and quick retrieval of clinical details for the citizens, as well as a platform for citizens to record self-monitoring health data. Thus, the app also facilitated the work of health care professionals with the integration and sharing of health records [5,7]. A medical app that contained medication, vaccine, and appointment records was convenient for the users of health care services. This helps to contribute to a user-friendly system that enhanced more patients’ use of the app. Among the eHealth app users in different studies, ease of use, user-friendliness, and availability of resources were the success factors facilitating the use of the app [20]. The eHealth app seems to empower the users to participate in health services, access health information, and manage their family members’ and their own health, which has contributed to the overall satisfaction (975/1242, 78.50%) with the eHealth app.

Similar to our previous studies in 2020, many participants learned about the eHRSS from others, including medical doctors, posters, television, and outdoor advertisement [7,21]. However, the occurrence of the coronavirus pandemic has raised public awareness of eHealth technology [22]. Our results showed that the COVID-19 vaccination program has become the major source for people to learn about the app. The practical use of the eHealth app, including COVID-19 vaccination record and vaccine pass, has encouraged a large group of citizens to download and adopt the eHealth app. Based on the systematic analysis of 8 studies from the United States, China, and Switzerland, patient engagement has been enhanced by eHealth technologies, as these supported contact tracing and improved access to surveillance data [23]. A group of Canadian scholars found that the use of an eHealth app could be enhanced and made available widely in a pandemic context when eHealth technologies are integrated with public health policy and programs, which in turn could facilitate the flow of information and communication [24]. These helped to explain why the downloading and acceptance of the eHealth app, as a medical informatics technology, had a large increase during the pandemic.

The participation of doctors was decisive to encourage the citizens to download and use the eHealth app. Our previous study in 2020 found that the actual use of the eHRSS among patients was also significantly associated with the enrollment among physicians [7]. Giving sharing consent to health care providers was one of the major enablers for people to download and use the app. However, if their doctor did not join eHealth, it is of no use for them to give sharing consent to the doctor. This may lower the perceived usefulness of the app and discourage people to adopt. In our result, the TPB implied that subjective norm, doctor’s recommendation, could largely determine the participants’ willingness to download and adopt the app. The downloading and acceptance processes have been found satisfactory in the responses, especially among the respondents in group 1. However, the respondents hesitated to adopt the app because of perceived complicated downloading procedures. The eHealth app had users with a wide range of demographic characteristics and different levels of technical proficiency. Besides, the elderly and less educated citizens might have difficulties in adopting mobile apps. It was also found that the respondents in group 2 reported a lower satisfaction rate with the app. Based on the TAM, perceived usefulness and perceived ease of use are the key factors in the process of adopting new technology. A cross-sectional study by Canadian medical practitioners found that perceived ease of use was the strongest facilitator for electronic health record use, whereas usefulness and ease of use were the main factors influencing system acceptance among nonusers [25]. A systematic review also stated that lower perceived ease of use may lead to resistance to further acceptance and require additional effort and time [26]. In our study, respondents who faced difficulties in the downloading and acceptance processes had reduced willingness to download and use the app.

Privacy was an important perceived barrier to the acceptability of the eHealth app. The respondents in group 3 were worried about their personal information and privacy. As supported by international studies, privacy was a common concern raised by the public about eHealth technologies [27], especially when patients’ lifestyles and activities were collected by multiple mobile health apps [28]. By contrast, our result showed that a significantly lower percentage of the users expressed concern about privacy, and that they had a generally high satisfaction rate with the app. Those who have already used the app valued their experience and benefits outweighed the privacy issue. This result was also suggested by a previous study on perceived benefits and concerns toward health information exchange [29]. Data security was also found to be a major barrier for non-enrollees not registering for the eHRSS in our 2020 study [7].

### Implication

More assistance and support should be provided regarding the perceived difficulties in using mobile apps. To enhance the acceptance rate among people who have not adopted or downloaded the app, the utility and benefit of the app should be emphasized among the public. We suggest further promoting the app through doctors by sharing the benefits of health management in using the app with the citizens. For future development, more sharable scope of the health record, such as laboratory results and radiographic images, and customized health information, including age-specific health care recommendations and tailored health tips, should be included.

Regarding the perception of difficulties in using mobile apps, the user interface and user experience should be further enhanced. The acceptance of the eHealth app requires a certain level of technology literacy and a fair understanding of digital technology [30]. To have a full experience of eHealth, users are required to develop fundamental skills in health, information, science, media, computer, and the internet [31]. The publicity channels could be used to educate and provide some quick tips to the citizens. Users should also be encouraged to manage their family members, who are less familiar with the mobile apps, via the eHealth app.

Regarding the privacy issue, the security and privacy measures applied to the eHealth app should be reinforced. Further, it is an effective way to ensure widespread participation in the eHealth app by emphasizing the utility and benefits of the app [29,32]. The strategy is to present positively framed messages to the participants [33]. The usefulness and convenience of the eHealth app should be emphasized as they were strong predictive factors of acceptance of the eHealth app. A high percentage of respondents agreed that using the app could enhance their experience of health services, their concerns about health information, their management of health, and improve the health of family members.

In our findings, doctors had an important role in determining people’s acceptance of the app. Doctors could recommend citizens managing the eHealth account and sharing function, which were the top-rated perceived enablers. The app could also improve the workflow of the doctors by allowing them to access patients’ health records that have been shared in the eHRSS. Doctor was an important source for citizens to acknowledge the eHealth app. Therefore, it was also important to introduce the eHealth app to doctors and health care providers, encourage them to manage patients’ health, and facilitate comanagement by patients and their family with the assistance of the eHealth app.

For future improvement, personalized and age-specific health care recommendations should be provided to facilitate a more patient-centered eHealth app [34]. Health information, health care recommendation, and support could be individualized to the patients. Tailored health information was processed and selected by human or computer algorithms from a database developed for the citizens. The self-monitoring health data recorded in the app by the citizens are also one of the sources for the database. With more self-input health data in the app (eg, BMI, health vital, and medication list), the data collected could be used to provide tailor-made health tips. Tailored health messages or recommendations could thus be individualized according to the patients’ needs that were able to command greater attention and were easier to be understood [35]. Health information could be specific to the age and chronic diseases or other personal backgrounds of the citizen, which could improve the design of the app.

### Conclusions

Overall, the respondents had a high satisfaction rate and a positive attitude toward continuing to adopt and recommend the app. The eHealth app seemed to empower citizens and their family members by enhancing their health information, self-management strategies, and experience with health services. However, privacy concerns and perceived difficulties in adopting were the major challenges of promoting eHealth. More comprehensive health records and tailored health information were recommended to be included for future improvement.

## Appendix

Multimedia Appendix 1 Factors predictive of downloading and adoption of the eHealth app by the Technology Acceptance Model (TAM).

Multimedia Appendix 2 Factors predictive of downloading and adoption of the eHealth app by the Technology Acceptance Model (TAM).

Multimedia Appendix 3 Factors predictive of downloading and acceptance of the eHealth app by the Theory of Planned Behavior (TPB).

Multimedia Appendix 4 Survey administered for the different respondents in this study.

Multimedia Appendix 5 Distribution of study participants according to downloading and acceptance of the eHealth app.

Multimedia Appendix 6 Sources where the study participants learnt about the eHealth app.

Multimedia Appendix 7 Perception on processes of current and future acceptance of the eHealth app.

Multimedia Appendix 8 Applicability of the eHealth app.

Multimedia Appendix 9 Perception of the eHealth app.

Multimedia Appendix 10 Expectations on future development of the eHealth app.

## Abbreviations

eHRSS: Electronic Health Record Sharing System
HA: Hospital Authority
TAM: Technology Acceptance Model
TPB: Theory of Planned Behavior
TRA: Theory of Reasoned Action

## Data Availability

The data used for the analyses are available upon reasonable request from the corresponding author.
