# Supplementary material for: Factors Associated With the Acceptance of an eHealth App for Electronic Health Record Sharing System: Population-Based Study
Source: J Med Internet Res. 2022 Dec 12;24(12):e40370. doi: 10.2196/40370 (PMC9793296; doi:10.2196/40370)
Supplement: Multimedia Appendix 1 [file jmir_v24i12e40370_app1.docx]

|  | **Number (n=20)** | **%** |
| --- | --- | --- |
| Completed Responses | 13 | 65% |
| Average Response Time | 7 mins 40 seconds |  |
| **Characteristics** | | |
| Age |  |  |
| 16-30 | 2 | 15.4% |
| 31-40 | 3 | 23.1% |
| 41-50 | 3 | 23.1% |
| 51-60 | 3 | 23.1% |
| 61-70 | 0 | 0% |
| >70 | 2 | 15.4% |
| Gender |  |  |
| Male | 5 | 38.5% |
| Female | 8 | 61.5% |
