# Supplementary material for: Factors Associated With the Acceptance of an eHealth App for Electronic Health Record Sharing System: Population-Based Study
Source: J Med Internet Res. 2022 Dec 12;24(12):e40370. doi: 10.2196/40370 (PMC9793296; doi:10.2196/40370)
Supplement: Multimedia Appendix 2 [file jmir_v24i12e40370_app2.docx]

| **Components** | **Items actual wording** | **Question number** | **Value** |
| --- | --- | --- | --- |
| Perceived Usefulness | a. It is convenient to get information about different government subsidized medical programmes | Q2ga | 1-5 |
|  | b. I can view my accurate health records | Q2gb | 1-5 |
|  | c. I can manage my eHealth account easily (e.g. update the communication means) | Q2gc | 1-5 |
|  | d. I can give sharing consents to healthcare providers easily so that they can view my health records | Q2gd | 1-5 |
|  | e. I can find the healthcare providers and doctors that participating different health programmes with ease | Q2ge | 1-5 |
|  | f. I can check the remaining balance and record of Elderly Health Care Voucher Scheme | Q2gf | 1-5 |
|  | g. I can show the vaccination record / QR code | Q2gg | 1-5 |
|  | h. It helps to manage my and my families’ health | Q2gh | 1-5 |
| Perceived Ease of use | a. It is difficult to search for the “醫健通eHealth” App in Mobile Application store (e.g. App Store, Google Play etc.) **[Reversed]** | Q3_a | 1-5 |
|  | b. I am satisfied with the downloading process | Q3_b | 1-5 |
|  | c. The authentication and login process is easy | Q3_c | 1-5 |
|  | d. I can easily find the function I want | Q3_d | 1-5 |
|  | e. The font and font size is easy to read | Q3_e | 1-5 |
|  | f. The icons and graphs are clear and easy to interpret | Q3_f | 1-5 |
|  | g. The health information is clear and informative | Q3_g | 1-5 |
|  | h. I have encountered situations or crashing app, no response, or unable to open on a frequent basis **[Reversed]** | Q3_h | 1-5 |
|  | i. The process of updating is quick | Q3_i | 1-5 |
| Behavioral Intention | Have you completed the authentication process and successfully login to the “醫健通eHealth” App? **[Recode: 1=Yes, 0=No/Not sure]** | Q2c | 1=Yes  2=No  3=Not sure |
|  | How often do you use the functions in “醫健通eHealth” App that do not require login? (e.g. access to Health News) | Q2d1 | 1-5 |
|  | How likely is that for you to continuously use the “醫健通eHealth” App? | Q10 | 1-5 |
| Perceived Barriers | a. My doctor does not participate in eHealth | Q2ha | 0-5 |
|  | b. I visit the same doctor every time and he/she has my complete medical record | Q2hb | 0-5 |
|  | c. I am healthy | Q2hc | 0-5 |
|  | d. Concerns about the personal privacy and data security | Q2hd | 0-5 |
|  | e. My doctor did not mention about/recommend/think it is necessary to use “醫健通eHealth” App | Q2he | 0-5 |
|  | f. I do not know how to use smart phone/mobile application | Q2hf | 0-5 |
|  | g. I want the health record kept strictly to myself and no others | Q2hg | 0-5 |
|  | h. I am not sure about the benefit from using the “醫健通eHealth” App | Q2hh | 0-5 |
|  | i. The procedure (including download, authentication, and normal operation) of using the “醫健通eHealth” app is complicated | Q2hi | 0-5 |
| Acceptance of Technology | Downloaded and used eHealth application = Yes  Downloaded but not used eHealth application = No  Not having downloaded and used eHealth application = No | Gp01 | Yes/No (0,1) |
| Demographics (significant) | Is student or not? | Work_stud | Yes/No (0,1) |
|  | Using Android or not? | Mobile_an | Yes/No (0,1) |
|  | Using Harmony OS or not? | Mobile_harmony | Yes/No (0,1) |
|  | Did you register the “Electronic Health Record Sharing System” (eHealth)? | enrol | Yes/No (0,1) |

| **Path** | | | **Beta coefficient** | **Standard** | **t Value** | **Pr > \|t\|** |
| --- | --- | --- | --- | --- | --- | --- |
|  |  |  |  | **Error** |  |  |
| Q2ga | <=== | Perceived_Usefulness | 0.66848 | 0.01585 | 42.184 | <.0001 |
| Q2gb | <=== | Perceived_Usefulness | 0.7229 | 0.01394 | 51.853 | <.0001 |
| Q2gc | <=== | Perceived_Usefulness | 0.79372 | 0.0113 | 70.258 | <.0001 |
| Q2gd | <=== | Perceived_Usefulness | 0.71579 | 0.0142 | 50.417 | <.0001 |
| Q2ge | <=== | Perceived_Usefulness | 0.75044 | 0.01293 | 58.031 | <.0001 |
| Q2gf | <=== | Perceived_Usefulness | 0.69956 | 0.01477 | 47.351 | <.0001 |
| Q2gg | <=== | Perceived_Usefulness | 0.67955 | 0.01547 | 43.928 | <.0001 |
| Q2gh | <=== | Perceived_Usefulness | 0.78639 | 0.01158 | 67.924 | <.0001 |
| Behavioral_Intention | <=== | Perceived_Usefulness | 0.03165 | 0.03201 | 0.9887 | 0.3228 |
| Q3_a_R | <=== | Perceived_Ease_of_use | 0.27811 | 0.02546 | 10.922 | <.0001 |
| Q3_b | <=== | Perceived_Ease_of_use | 0.59338 | 0.01843 | 32.203 | <.0001 |
| Q3_c | <=== | Perceived_Ease_of_use | 0.63899 | 0.017 | 37.582 | <.0001 |
| Q3_d | <=== | Perceived_Ease_of_use | 0.80226 | 0.01123 | 71.419 | <.0001 |
| Q3_e | <=== | Perceived_Ease_of_use | 0.70431 | 0.0148 | 47.578 | <.0001 |
| Q3_f | <=== | Perceived_Ease_of_use | 0.80186 | 0.01125 | 71.291 | <.0001 |
| Q3_g | <=== | Perceived_Ease_of_use | 0.79413 | 0.01154 | 68.843 | <.0001 |
| Q3_h_R | <=== | Perceived_Ease_of_use | 0.33704 | 0.02453 | 13.74 | <.0001 |
| Q3_i | <=== | Perceived_Ease_of_use | 0.62344 | 0.0175 | 35.627 | <.0001 |
| Perceived_Usefulness | <=== | Perceived_Ease_of_use | 0.51991 | 0.0218 | 23.852 | <.0001 |
| Behavioral_Intention | <=== | Perceived_Ease_of_use | 0.19159 | 0.03232 | 5.9275 | <.0001 |
| Q10 | <=== | Behavioral_Intention | 0.19759 | 0.0253 | 7.8085 | <.0001 |
| Q2c_R | <=== | Behavioral_Intention | 0.74601 | 0.02409 | 30.973 | <.0001 |
| Q2d1 | <=== | Behavioral_Intention | 0.25722 | 0.02514 | 10.232 | <.0001 |
| Acceptance | <=== | Behavioral_Intention | 0.88637 | 0.02536 | 34.948 | <.0001 |
| Q2ha | <=== | Perceived_Barriers | 0.36142 | 0.02662 | 13.579 | <.0001 |
| Q2hb | <=== | Perceived_Barriers | 0.44208 | 0.02509 | 17.618 | <.0001 |
| Q2hc | <=== | Perceived_Barriers | 0.45445 | 0.02483 | 18.299 | <.0001 |
| Q2hd | <=== | Perceived_Barriers | 0.50141 | 0.02381 | 21.06 | <.0001 |
| Q2he | <=== | Perceived_Barriers | 0.46775 | 0.02455 | 19.051 | <.0001 |
| Q2hf | <=== | Perceived_Barriers | 0.55536 | 0.02255 | 24.631 | <.0001 |
| Q2hg | <=== | Perceived_Barriers | 0.5421 | 0.02286 | 23.709 | <.0001 |
| Q2hh | <=== | Perceived_Barriers | 0.64522 | 0.02035 | 31.713 | <.0001 |
| Q2hi | <=== | Perceived_Barriers | 0.51673 | 0.02346 | 22.027 | <.0001 |
| gp01 | <=== | Acceptance | 1.14687 | 0.00506 | 226.8 | <.0001 |
| Perceived_Usefulness | <=== | age | 0.07424 | 0.0254 | 2.9225 | 0.0035 |
| Perceived_Usefulness | <=== | work_stud | -0.0942 | 0.02537 | -3.713 | 0.0002 |
| Perceived_Barriers | <=== | mobile_an | -0.0854 | 0.02956 | -2.888 | 0.0039 |
| Perceived_Barriers | <=== | enrol | -0.0667 | 0.0296 | -2.255 | 0.0241 |
