# Supplementary material for: Factors Associated With the Acceptance of an eHealth App for Electronic Health Record Sharing System: Population-Based Study
Source: J Med Internet Res. 2022 Dec 12;24(12):e40370. doi: 10.2196/40370 (PMC9793296; doi:10.2196/40370)
Supplement: Multimedia Appendix 3 [file jmir_v24i12e40370_app3.docx]

|  |  |  |  |
| --- | --- | --- | --- |
| **Components** | **Items actual wording** | **Question Number** | **Value** |
| Attitude | a. It is convenient to get information about different government subsidized medical programmes | Q2ga | 1-5 |
|  | b. I can view my accurate health records | Q2gb | 1-5 |
|  | c. I can manage my eHealth account easily (e.g. update the communication means) | Q2gc | 1-5 |
|  | d. I can give sharing consents to healthcare providers easily so that they can view my health records | Q2gd | 1-5 |
|  | e. I can find the healthcare providers and doctors that participating different health programmes with ease | Q2ge | 1-5 |
|  | f. I can check the remaining balance and record of Elderly Health Care Voucher Scheme | Q2gf | 1-5 |
|  | g. I can show the vaccination record / QR code | Q2gg | 1-5 |
|  | h. It helps to manage my and my families’ health | Q2gh | 1-5 |
| Subjective Norm | i. My friend recommends me to use the “醫健通eHealth” App | Q2gi | 1-5 |
|  | j. My family recommend me to use the “醫健通eHealth” App | Q2gj | 1-5 |
|  | k. My doctor recommends me to use the “醫健通eHealth” App | Q2gk | 1-5 |
|  | l. Government’s advertisement of the “醫健通eHealth” App | Q2gl | 1-5 |
| Perceived Behavioral Control | c. The authentication and login process is easy | Q3_c | 0-5 |
|  | d. I can easily find the function I want | Q3_d | 0-5 |
|  | g. The health information is clear and informative | Q3_g | 0-5 |
|  | h. I have encountered situations or crashing app, no response, or unable to open on a frequent basis **[Reversed]** | Q3_h | 1-5 |
|  | i. The process of updating is quick | Q3_i | 0-5 |
|  | k. Overall, the App is simple and easy to use | Q3_k | 0-5 |
| Behavioral Intention | How likely is that for you to continuously use the “醫健通eHealth” App? | Q10 | 1-5 |
|  | How likely would you recommend your family/friends/peers to download the “醫健通eHealth” App? | Q11 | 1-5 |
|  | Have you completed the authentication process and successfully login to the “醫健通eHealth” App? **[Recode: 1=Yes, 0=No/Not sure]** | Q2c | 1=Yes  2=No  3=Not sure |
|  | How often do you use the functions in “醫健通eHealth” App that do not require login? (e.g. access to Health News) | Q2d1 | 1-5 |
| Behavior | Downloaded and used eHealth application = Yes  Downloaded but not used eHealth application = No  Not having downloaded and used eHealth application = No | Gp01 | Yes/No (0,1) |

| Path | | | Beta coefficient | Standard | t Value | Pr > \|t\| |
| --- | --- | --- | --- | --- | --- | --- |
|  |  |  |  | Error |  |  |
| Q2ga | <=== | Attitude | 0.6497 | 0.01666 | 38.999 | <.0001 |
| Q2gb | <=== | Attitude | 0.6947 | 0.01513 | 45.923 | <.0001 |
| Q2gc | <=== | Attitude | 0.7691 | 0.01241 | 61.971 | <.0001 |
| Q2gd | <=== | Attitude | 0.693 | 0.01519 | 45.632 | <.0001 |
| Q2ge | <=== | Attitude | 0.7296 | 0.01388 | 52.586 | <.0001 |
| Q2gf | <=== | Attitude | 0.6817 | 0.01558 | 43.751 | <.0001 |
| Q2gg | <=== | Attitude | 0.6622 | 0.01624 | 40.767 | <.0001 |
| Q2gh | <=== | Attitude | 0.7742 | 0.01222 | 63.369 | <.0001 |
| Behavioral_Intention | <=== | Attitude | 0.2987 | 0.03726 | 8.0173 | <.0001 |
| Q2gi | <=== | Subjective_Norm | 0.8848 | 0.00804 | 110 | <.0001 |
| Q2gj | <=== | Subjective_Norm | 0.8878 | 0.00795 | 111.7 | <.0001 |
| Q2gk | <=== | Subjective_Norm | 0.7673 | 0.0123 | 62.38 | <.0001 |
| Q2gl | <=== | Subjective_Norm | 0.6423 | 0.01674 | 38.373 | <.0001 |
| Behavioral_Intention | <=== | Subjective_Norm | 0.3702 | 0.03668 | 10.091 | <.0001 |
| Q3_c | <=== | Perceived_Behavioral_Control | 0.6551 | 0.01703 | 38.472 | <.0001 |
| Q3_d | <=== | Perceived_Behavioral_Control | 0.8327 | 0.01101 | 75.604 | <.0001 |
| Q3_g | <=== | Perceived_Behavioral_Control | 0.7472 | 0.01392 | 53.696 | <.0001 |
| Q3_h_R | <=== | Perceived_Behavioral_Control | 0.3302 | 0.02515 | 13.127 | <.0001 |
| Q3_i | <=== | Perceived_Behavioral_Control | 0.6423 | 0.01744 | 36.831 | <.0001 |
| Q3_k | <=== | Perceived_Behavioral_Control | 0.7855 | 0.01259 | 62.379 | <.0001 |
| Behavior | <=== | Perceived_Behavioral_Control | 0.1381 | 0.02923 | 4.724 | <.0001 |
| Q10 | <=== | Behavioral_Intention | 0.7587 | 0.01768 | 42.909 | <.0001 |
| Q11 | <=== | Behavioral_Intention | 0.8577 | 0.0169 | 50.764 | <.0001 |
| Q2c_R | <=== | Behavioral_Intention | 0.1511 | 0.02821 | 5.3569 | <.0001 |
| Q2d1 | <=== | Behavioral_Intention | 0.2689 | 0.02698 | 9.9667 | <.0001 |
| Behavior | <=== | Behavioral_Intention | 0.1306 | 0.03012 | 4.3355 | <.0001 |
| gp01 | <=== | Behavior | 1.0012 | 0.00834 | 120.1 | <.0001 |
| Attitude | ===> | Subjective_Norm | 0.3648 | 0.01323 | 27.578 | <.0001 |
| Subjective_Norm | ===> | Perceived_Behavioral_Control | 0.2691 | 0.02119 | 12.7 | <.0001 |
| Attitude | <=== | Subjective_Norm | 0.356 | 0.02645 | 13.46 | <.0001 |
| Attitude | <==> | Perceived_Behavioral_Control | 0.239 | 0.02389 | 10.005 | <.0001 |
| Subjective_Norm | <=== | Perceived_Behavioral_Control | 0.1136 | 0.00894 | 12.7 | <.0001 |
