# Supplementary material for: Factors Associated With the Acceptance of an eHealth App for Electronic Health Record Sharing System: Population-Based Study
Source: J Med Internet Res. 2022 Dec 12;24(12):e40370. doi: 10.2196/40370 (PMC9793296; doi:10.2196/40370)
Supplement: Multimedia Appendix 4 [file jmir_v24i12e40370_app4.docx]

**Supplementary Legends**

**Supplementary File 1 Survey for respondents have enrolled in the eHRSS, downloaded, and adopted eHealth app**

**Supplementary File 2 Survey for respondents have enrolled in eHRSS, downloaded the eHealth app, but not adopted the application**

**Supplementary File 3 Survey for respondents have enrolled in eHRSS, but have not downloaded nor adopted the app**

**Supplementary File 1 Survey for respondents have enrolled in the eHRSS, downloaded, and adopted eHealth app**
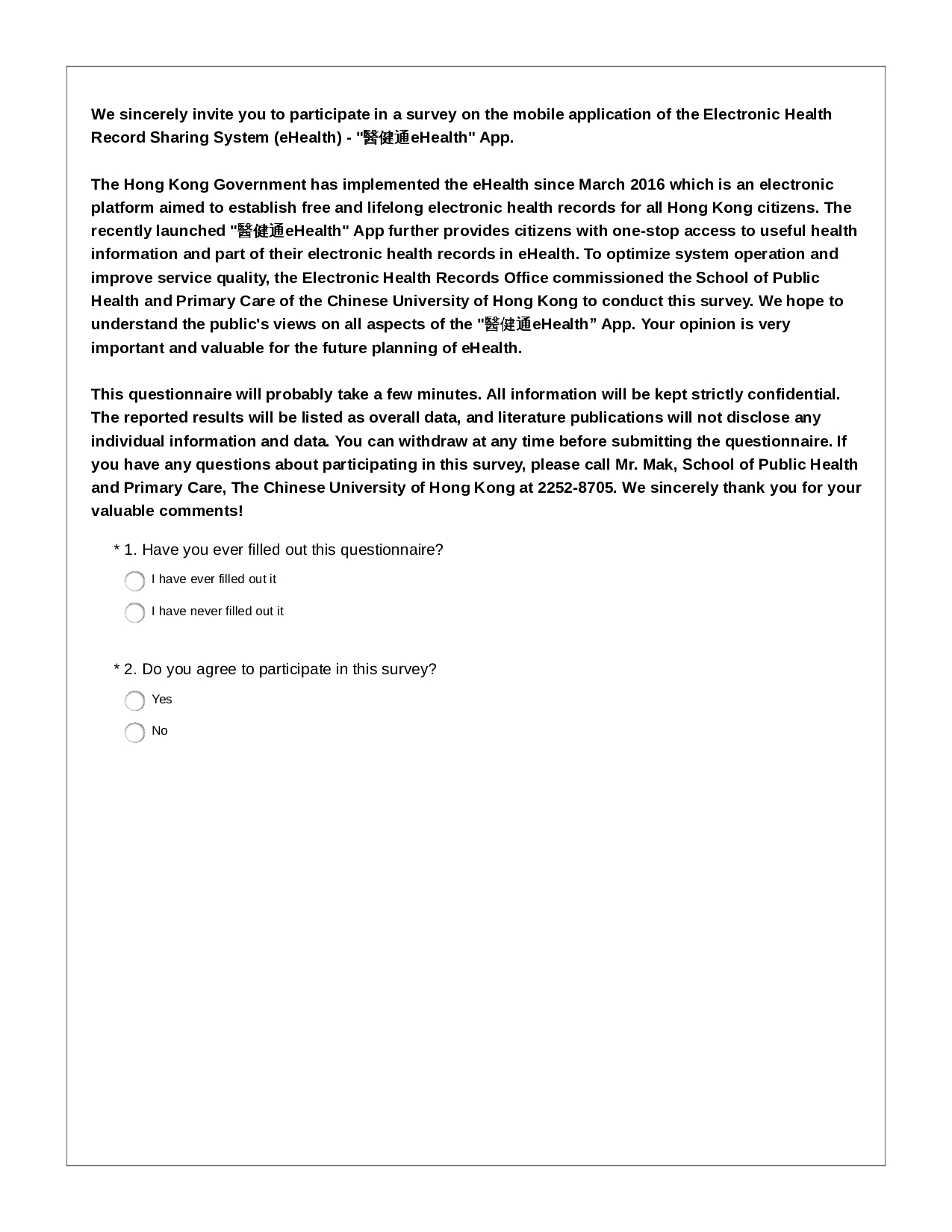

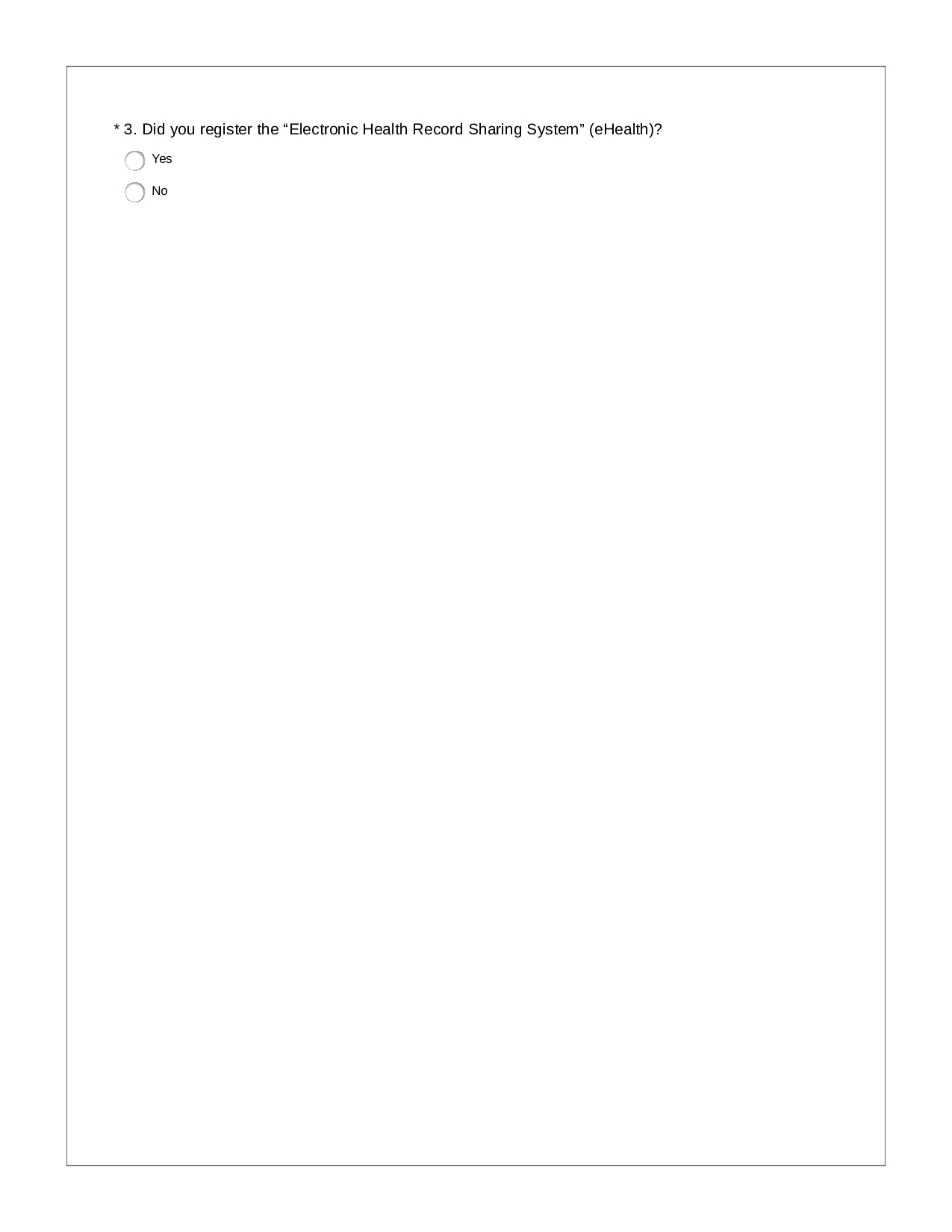

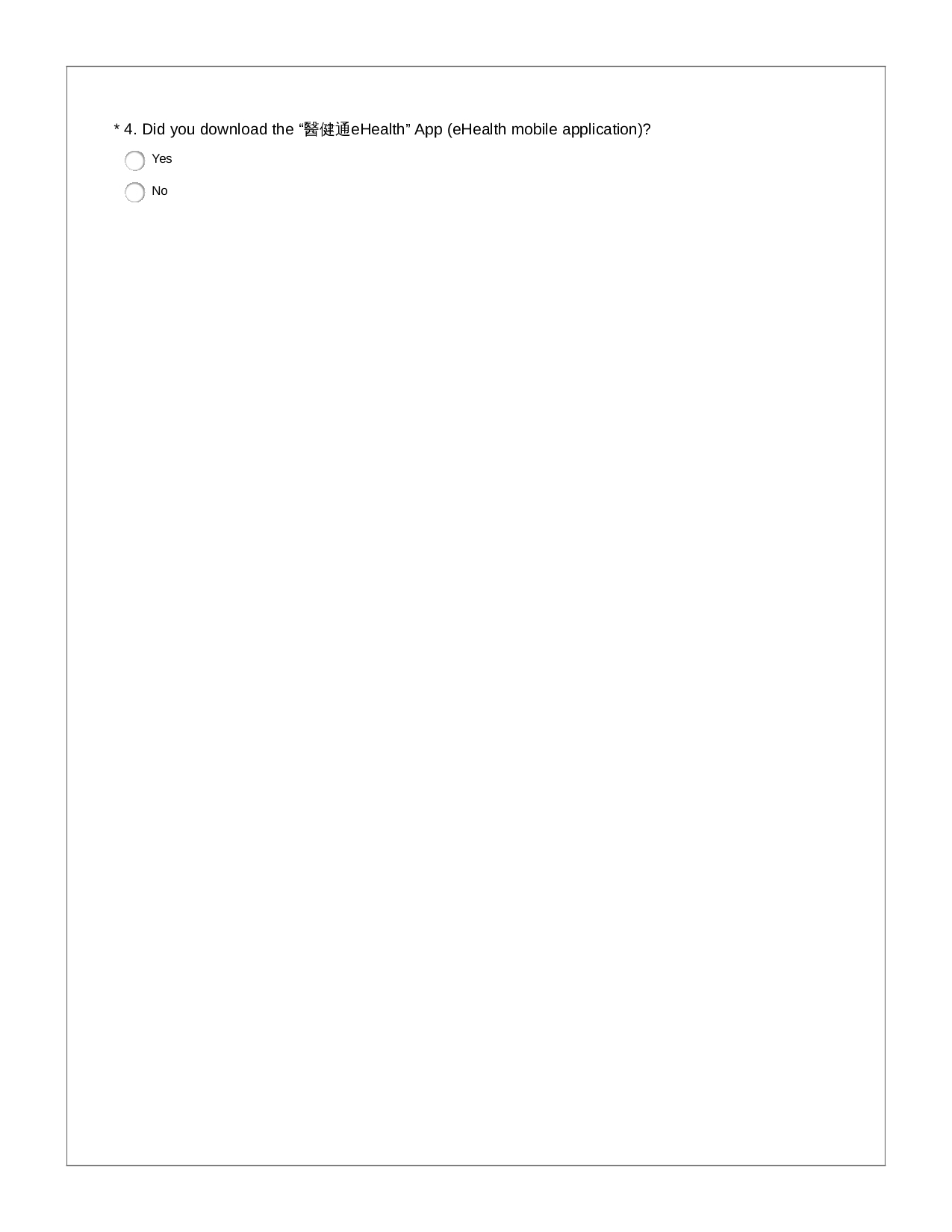

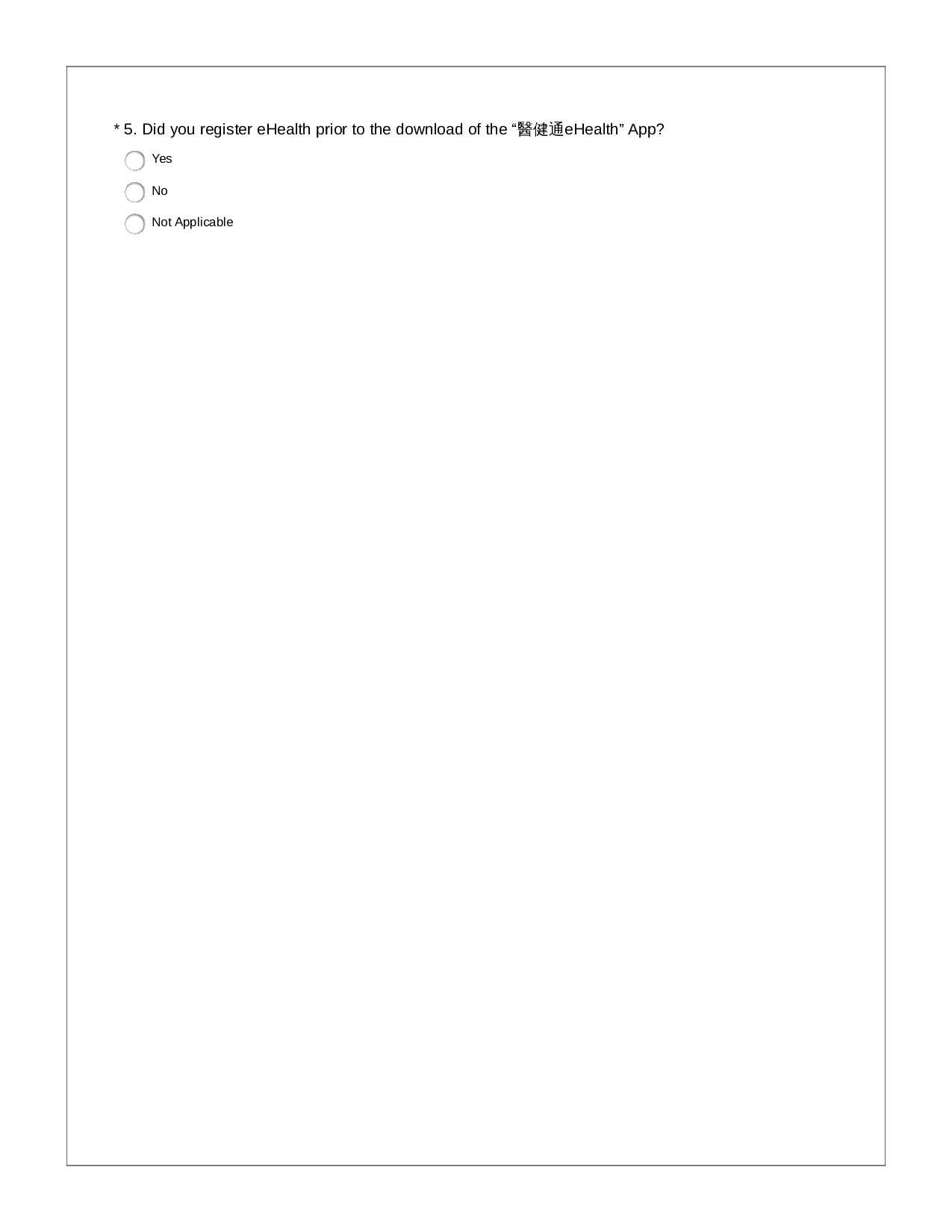

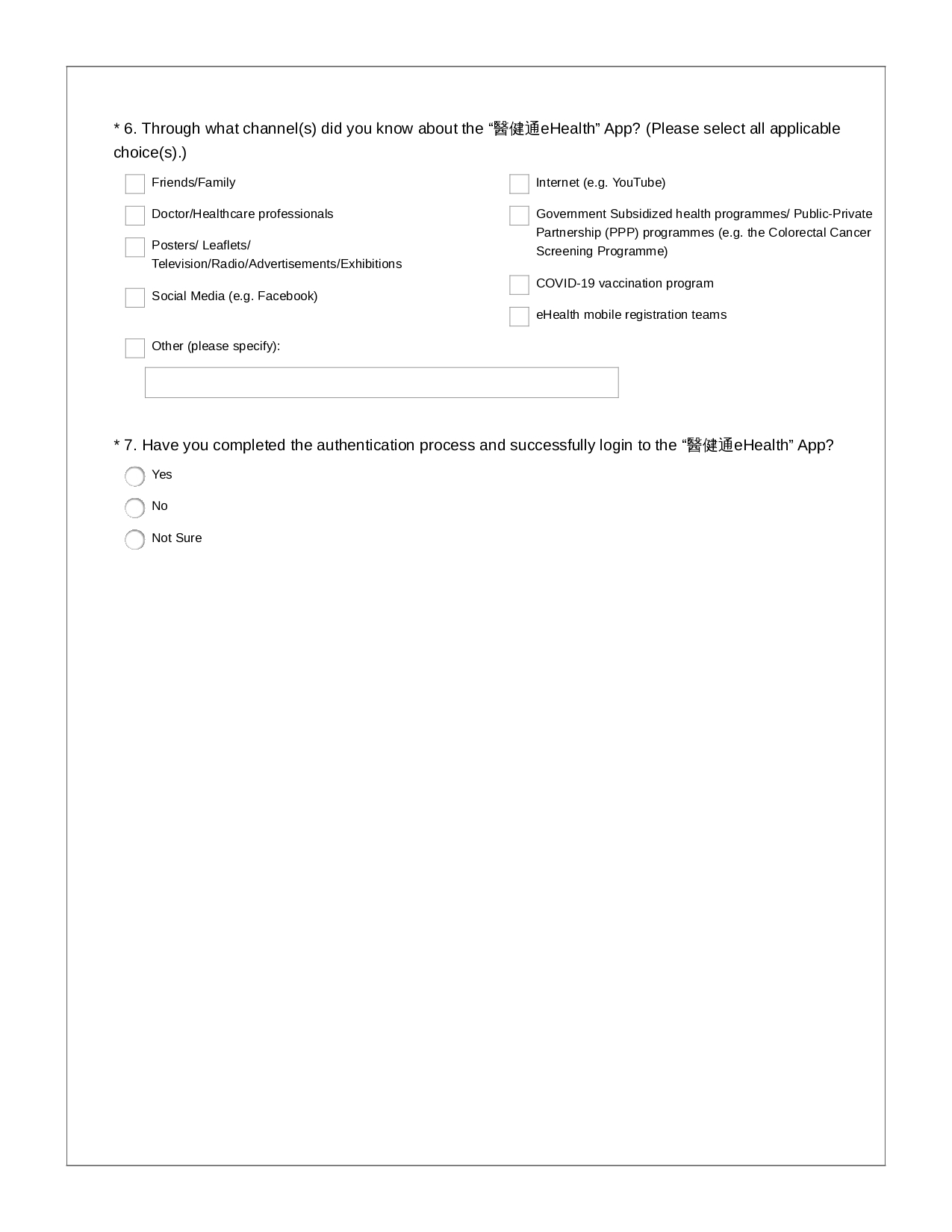

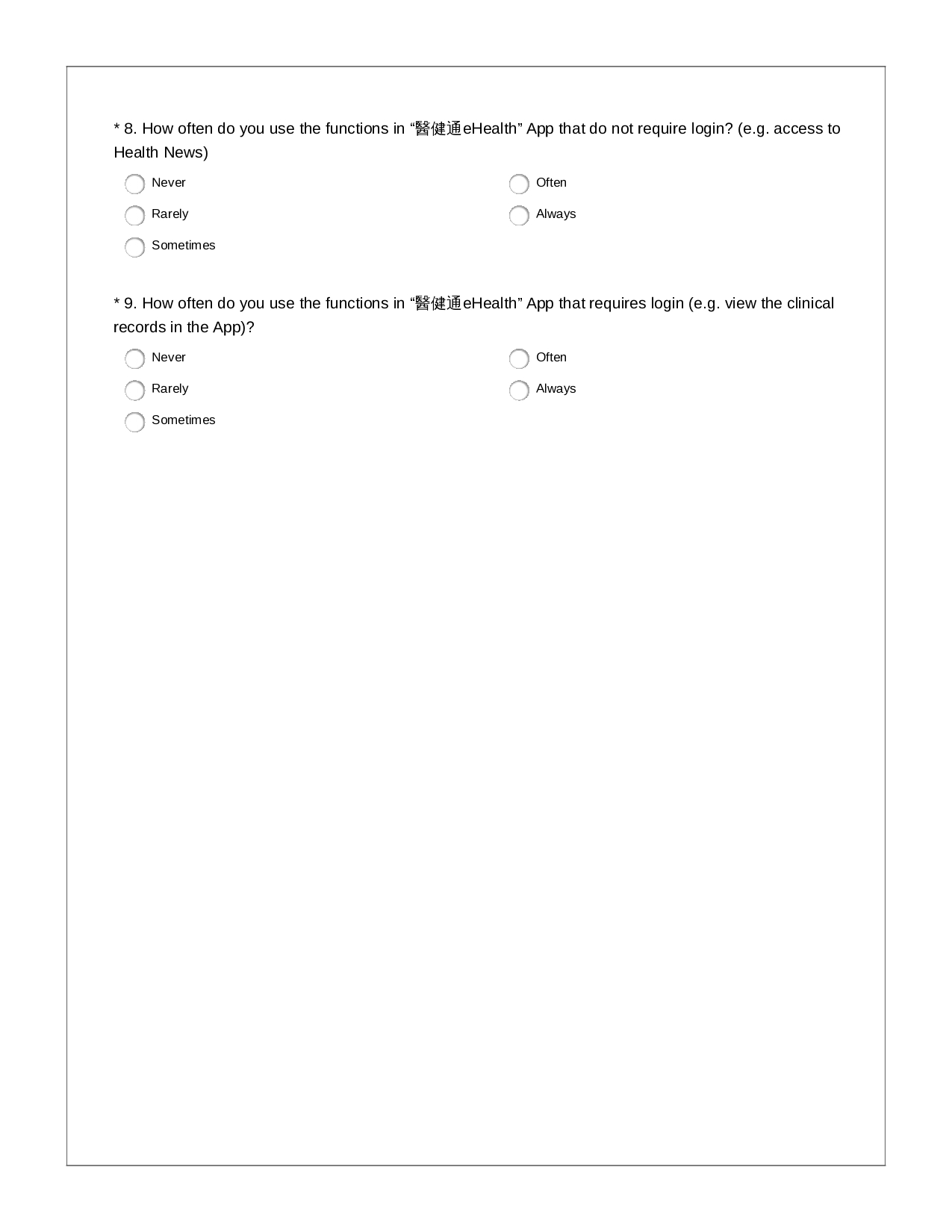

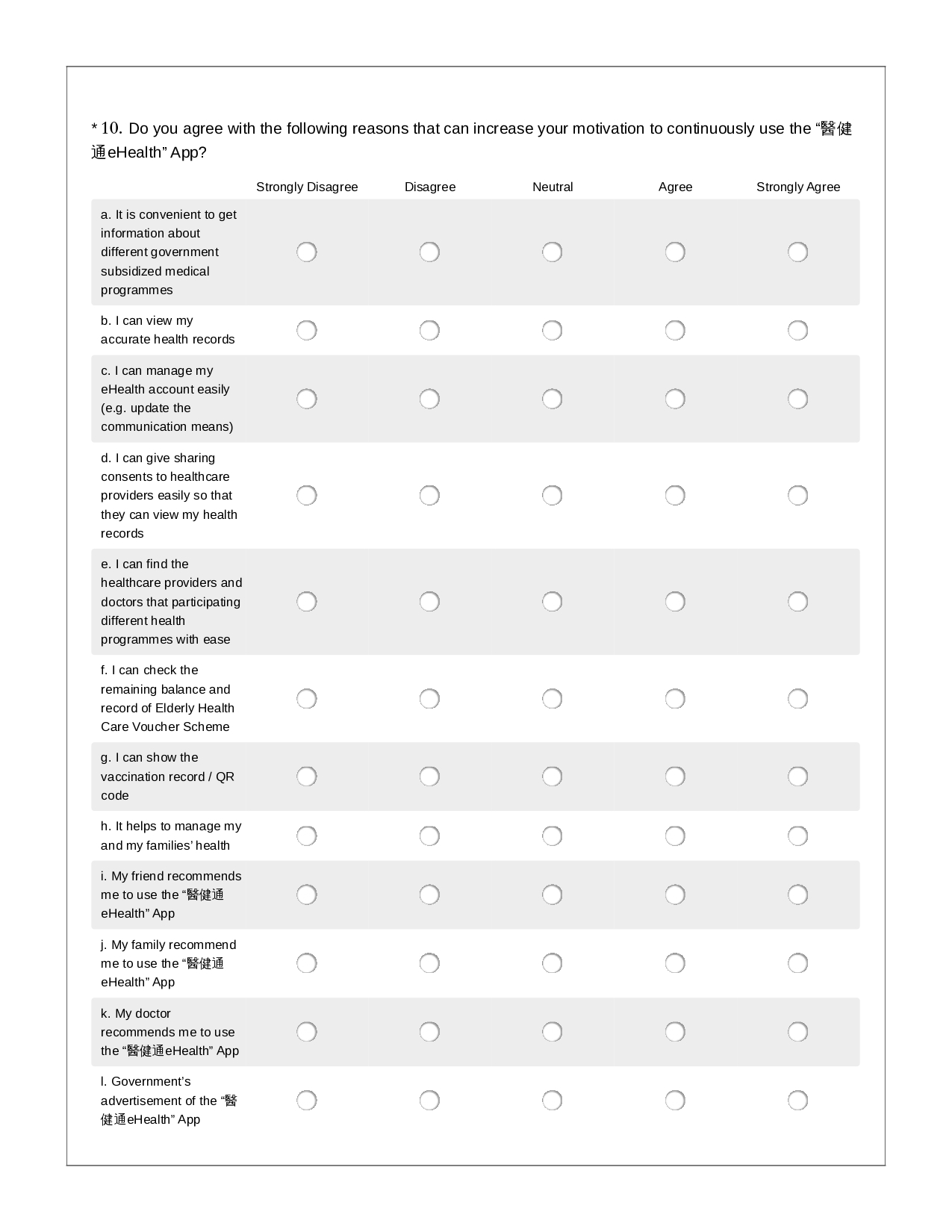

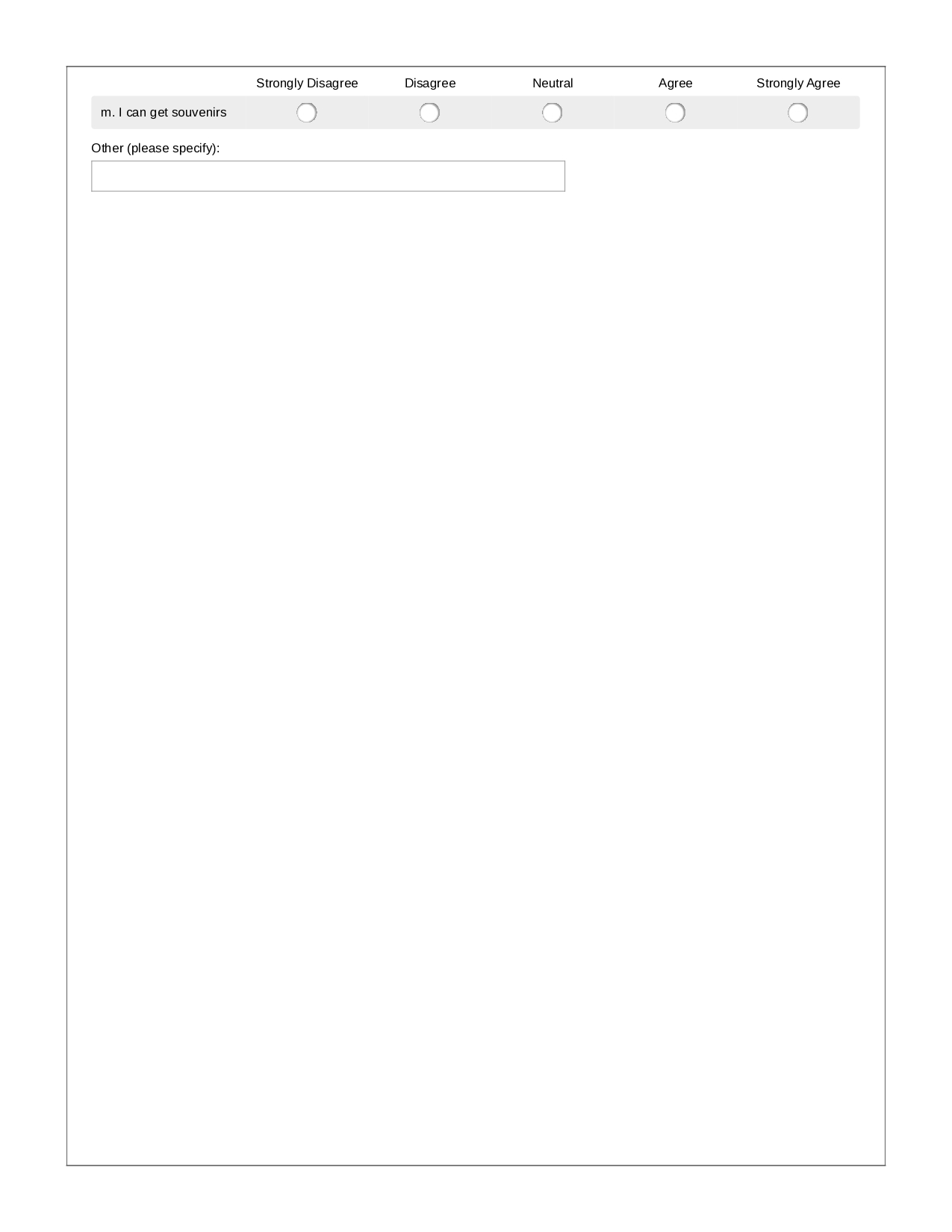

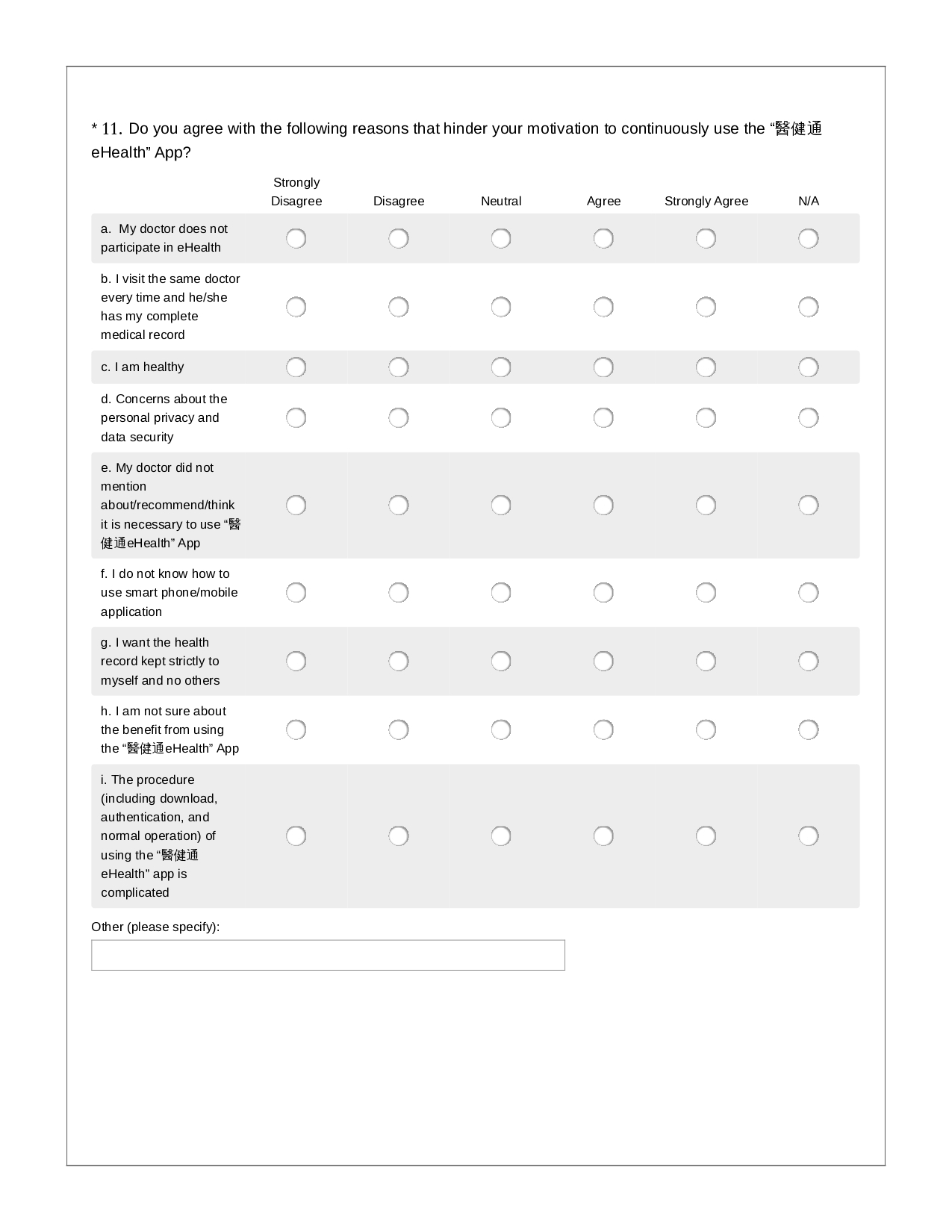

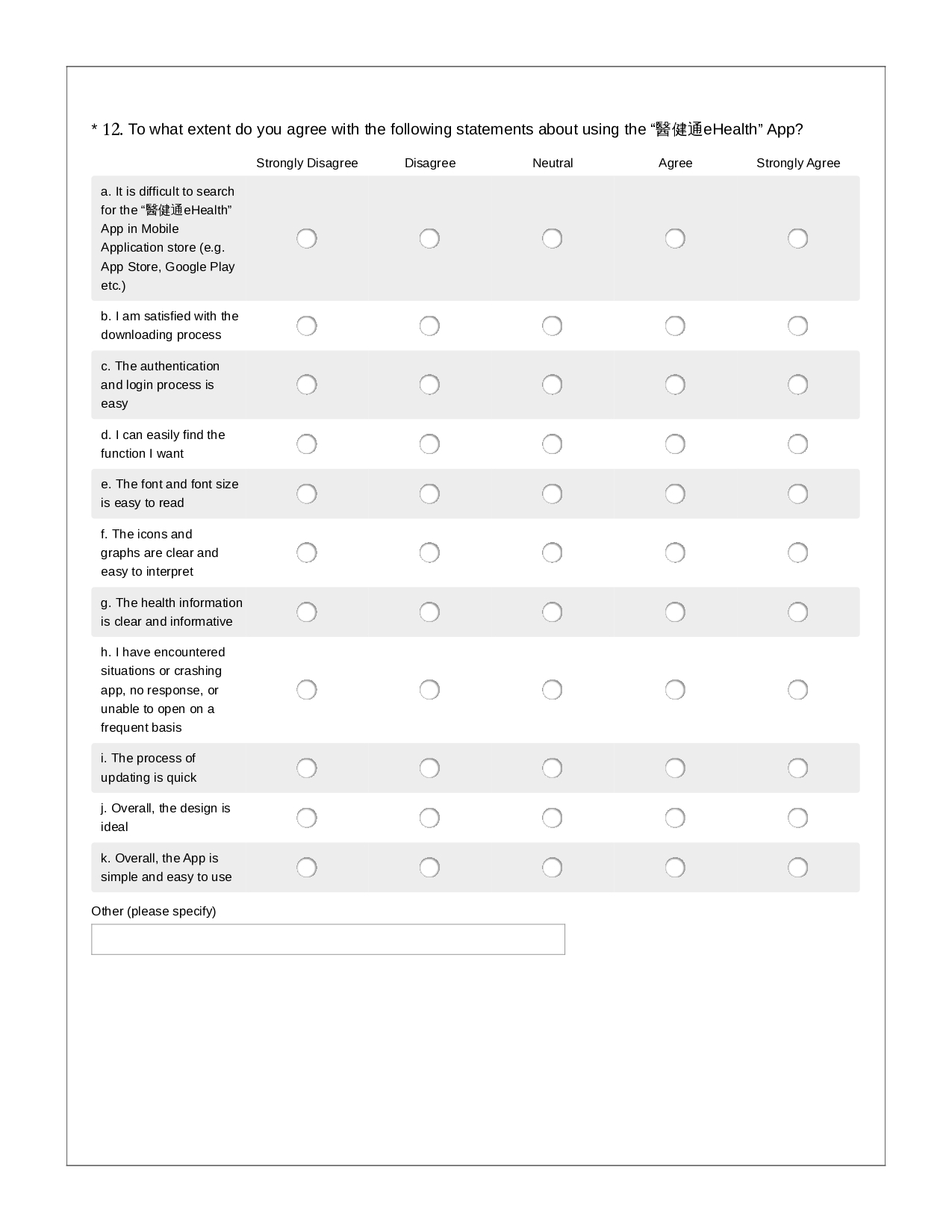

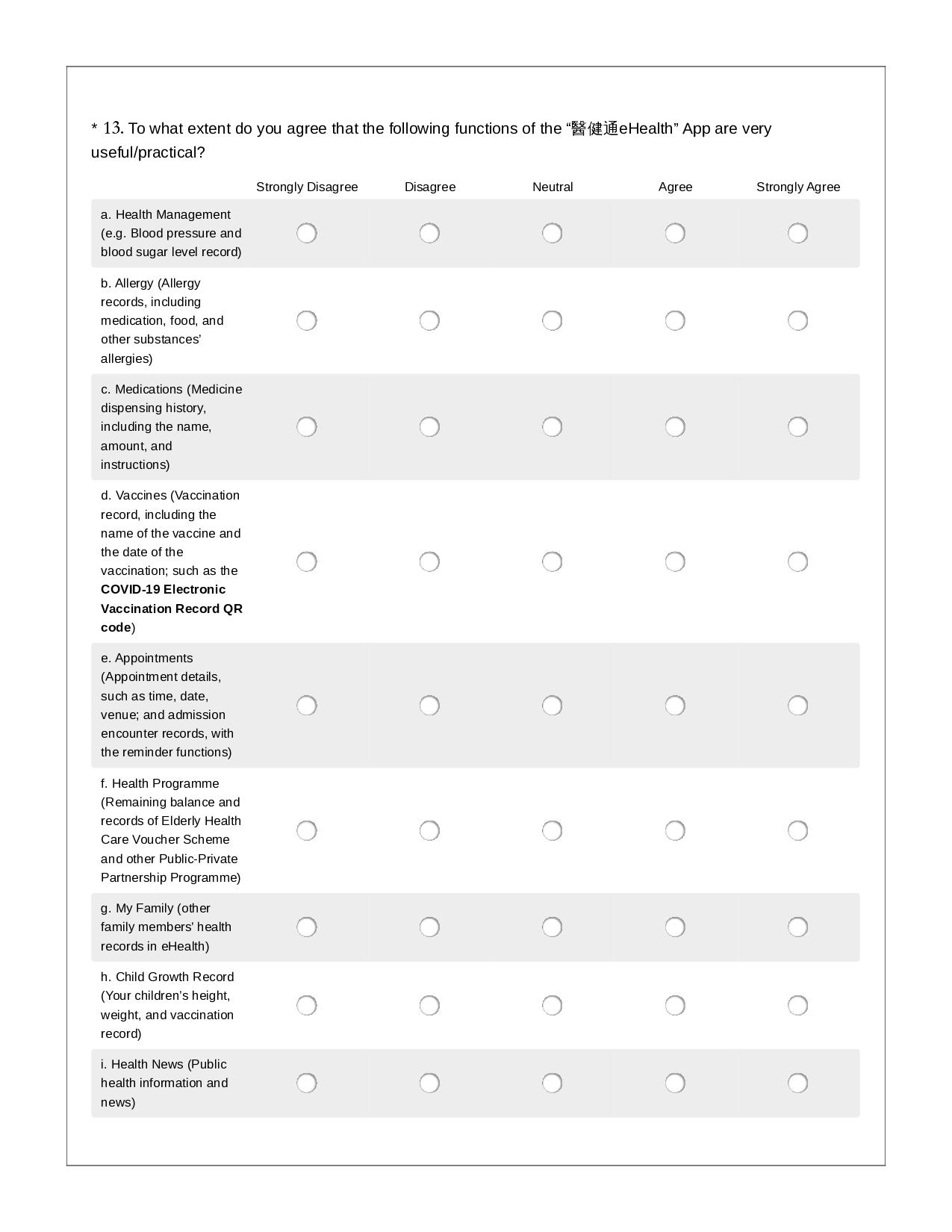

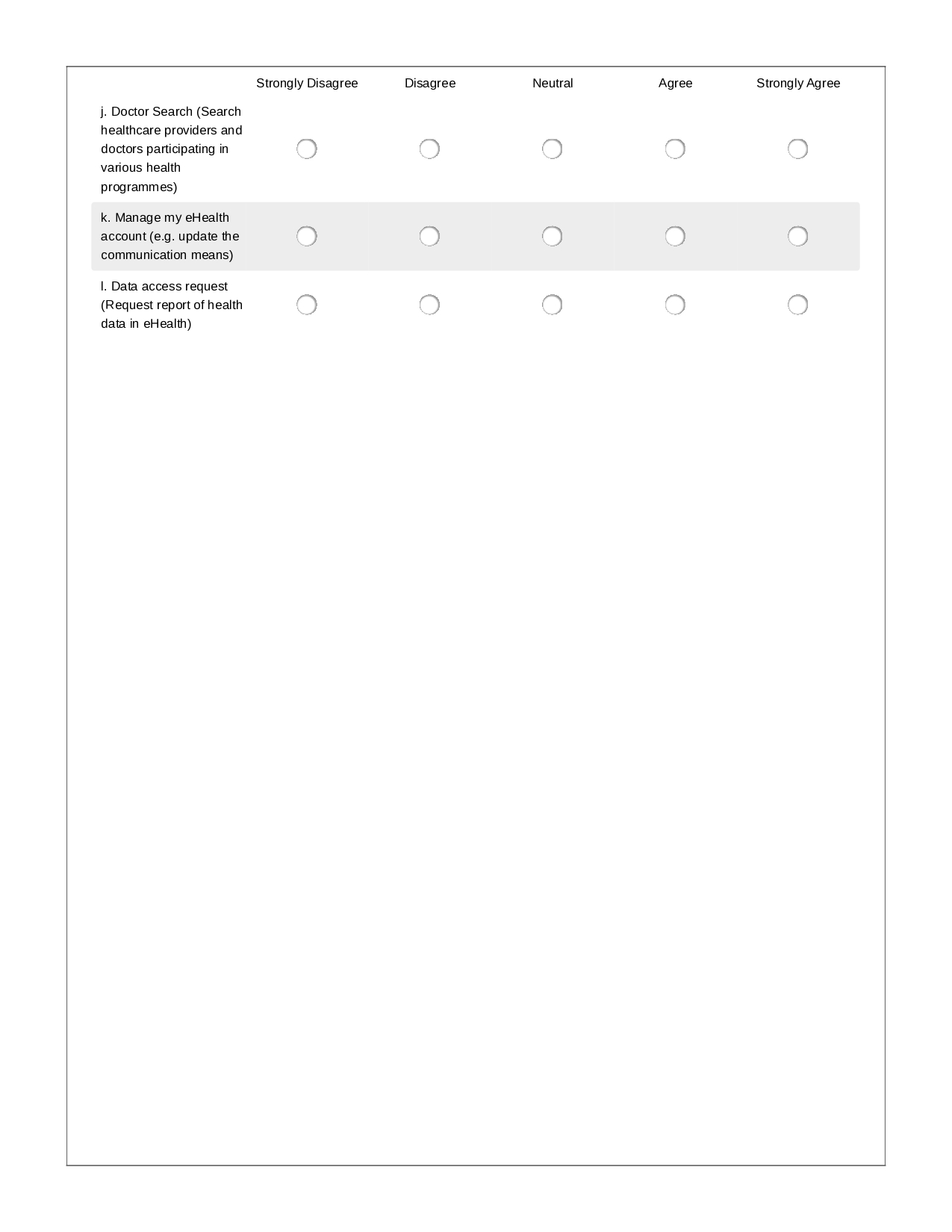

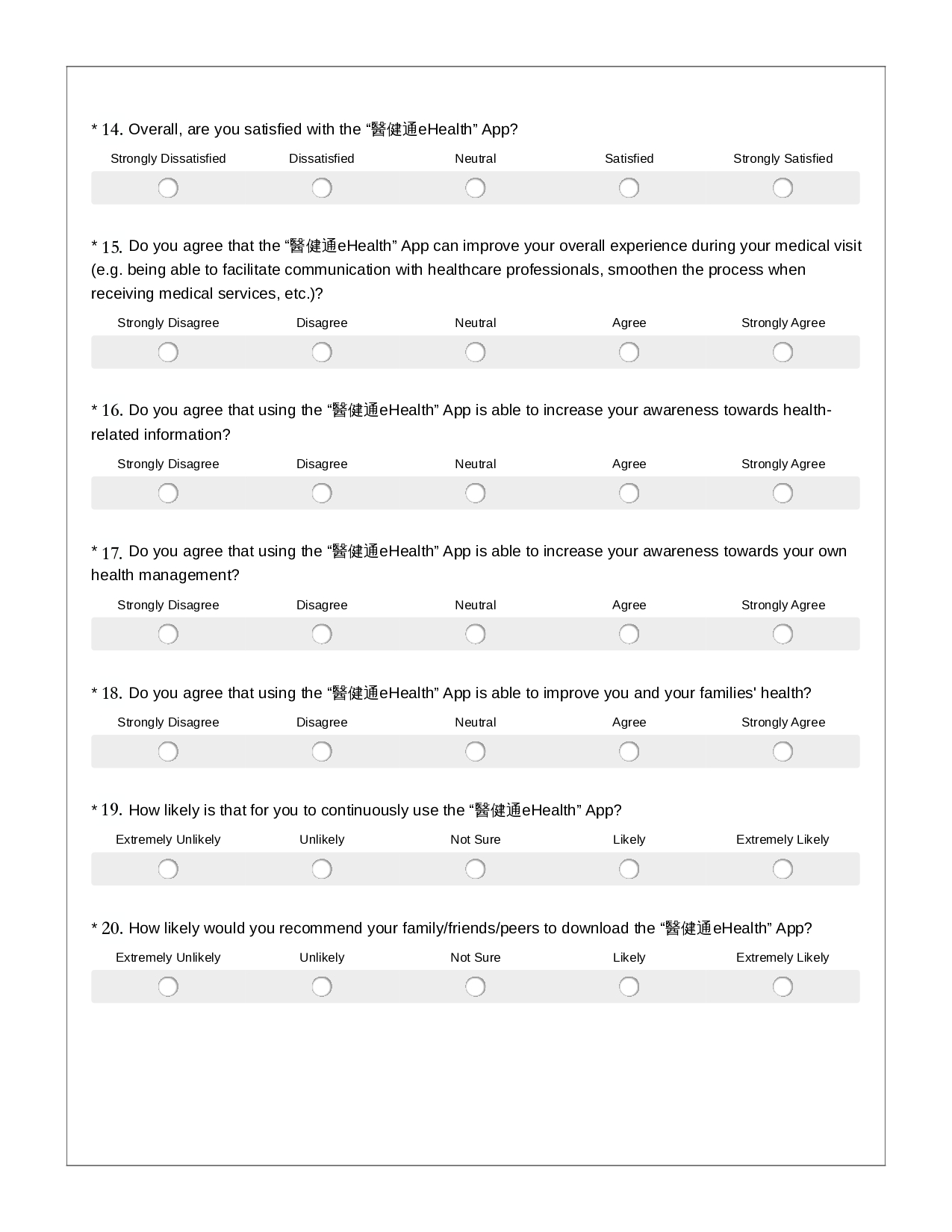

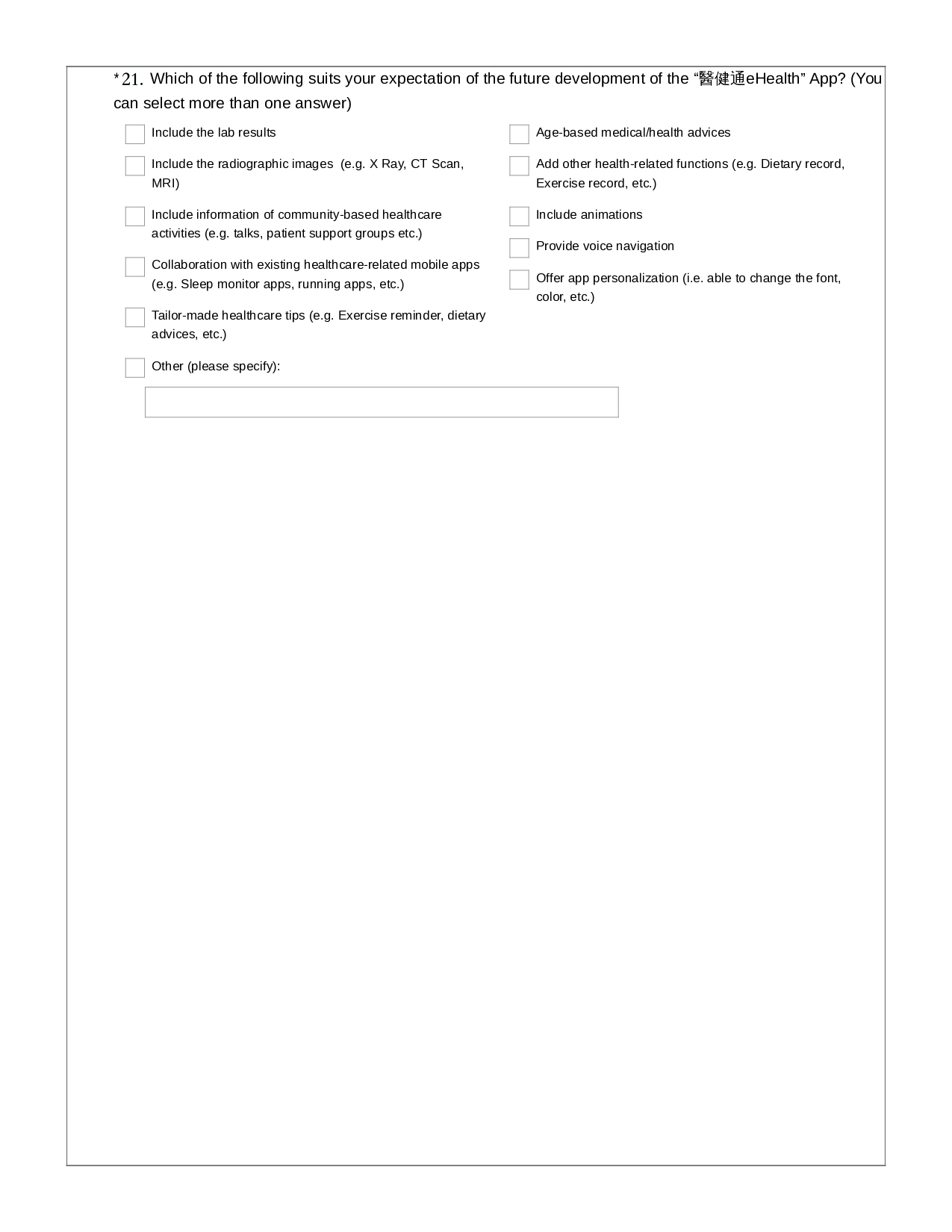

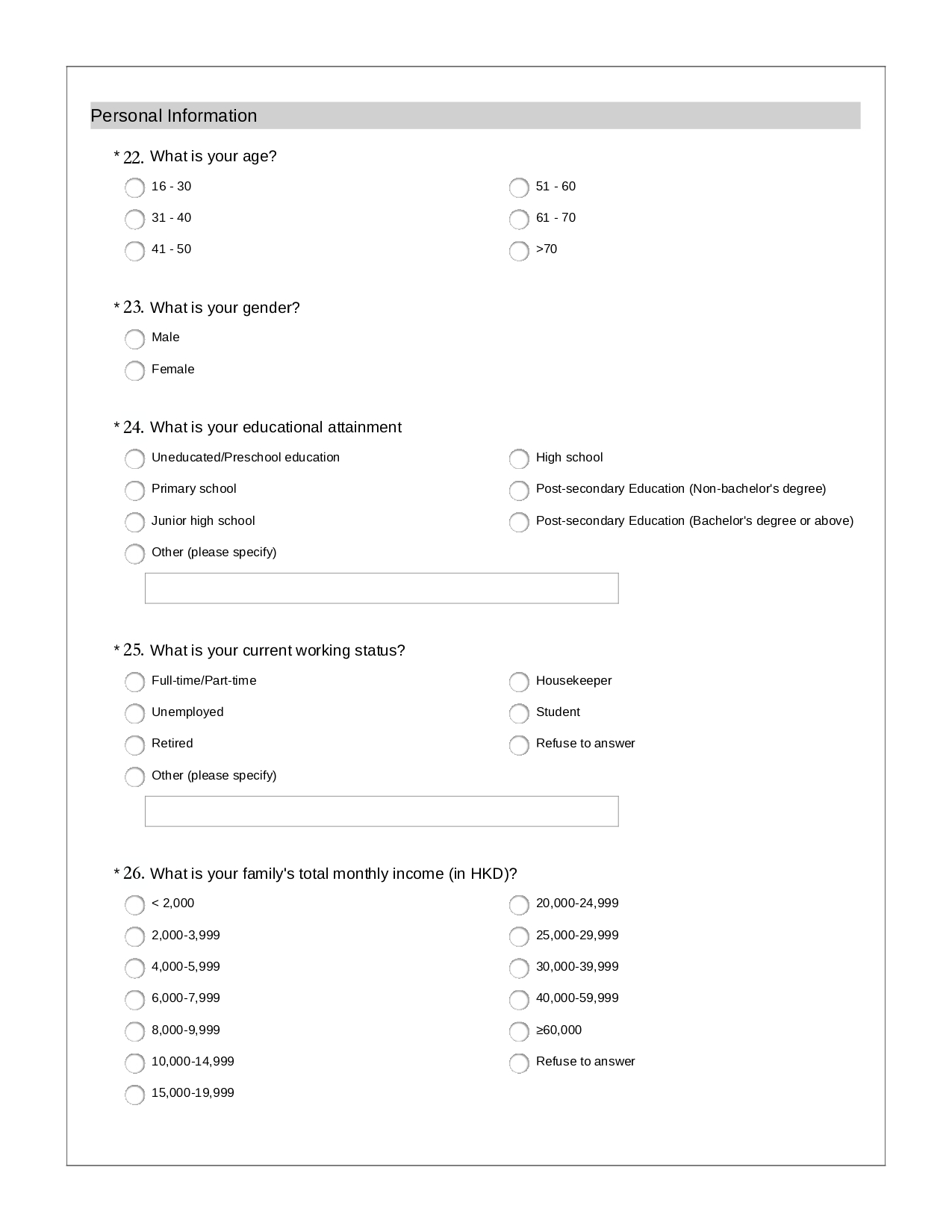

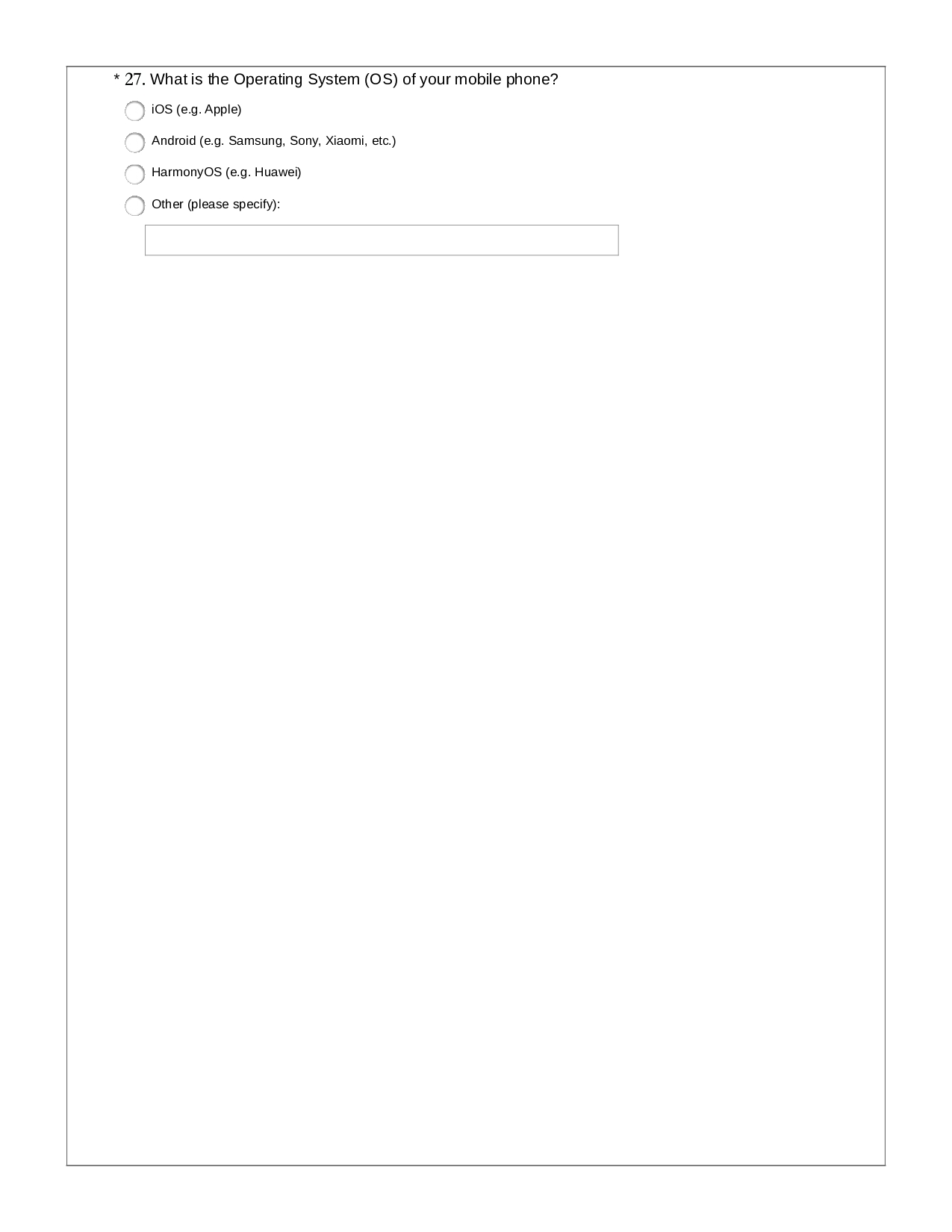


**Supplementary File 2 Survey for respondents have enrolled in eHRSS, downloaded the eHealth app, but not adopted the application
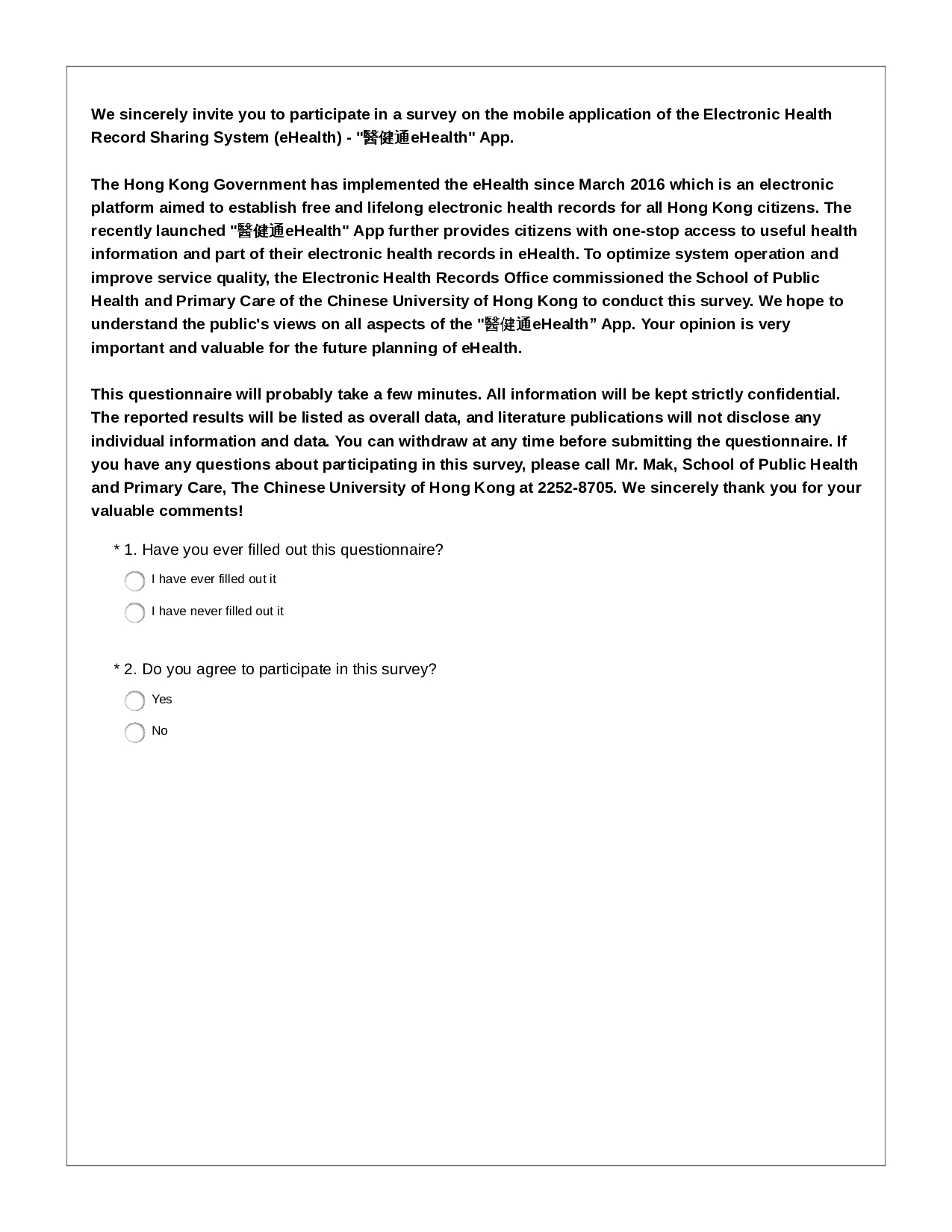

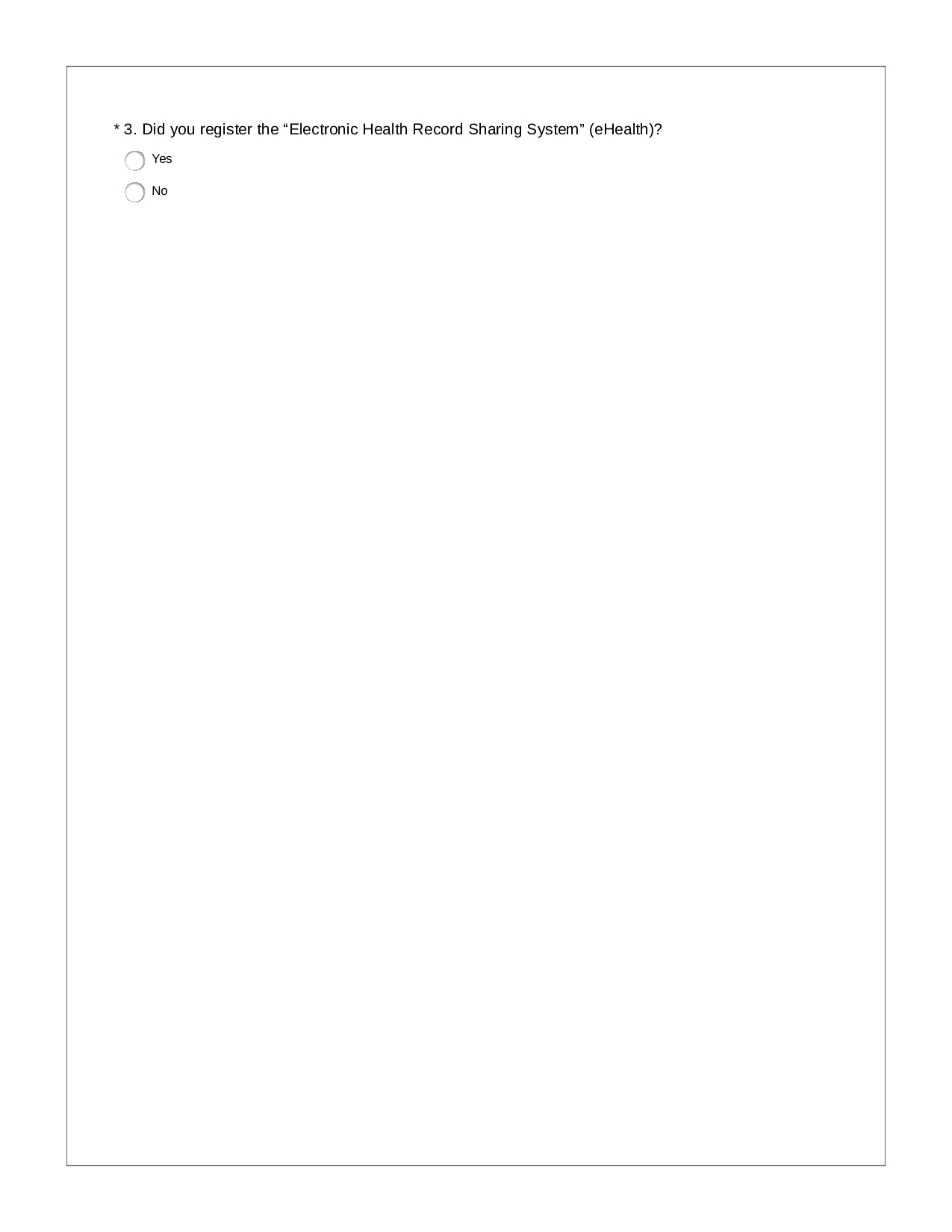

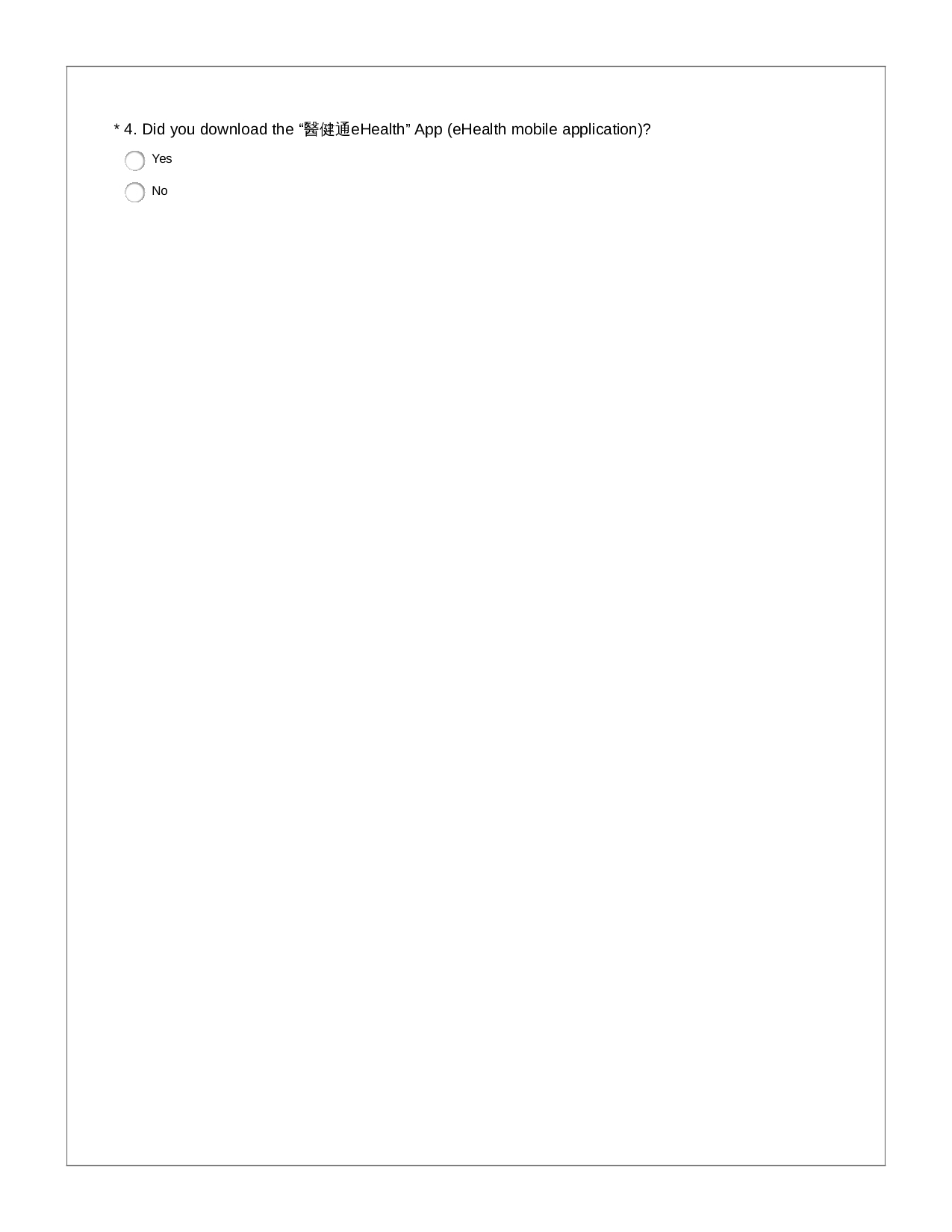

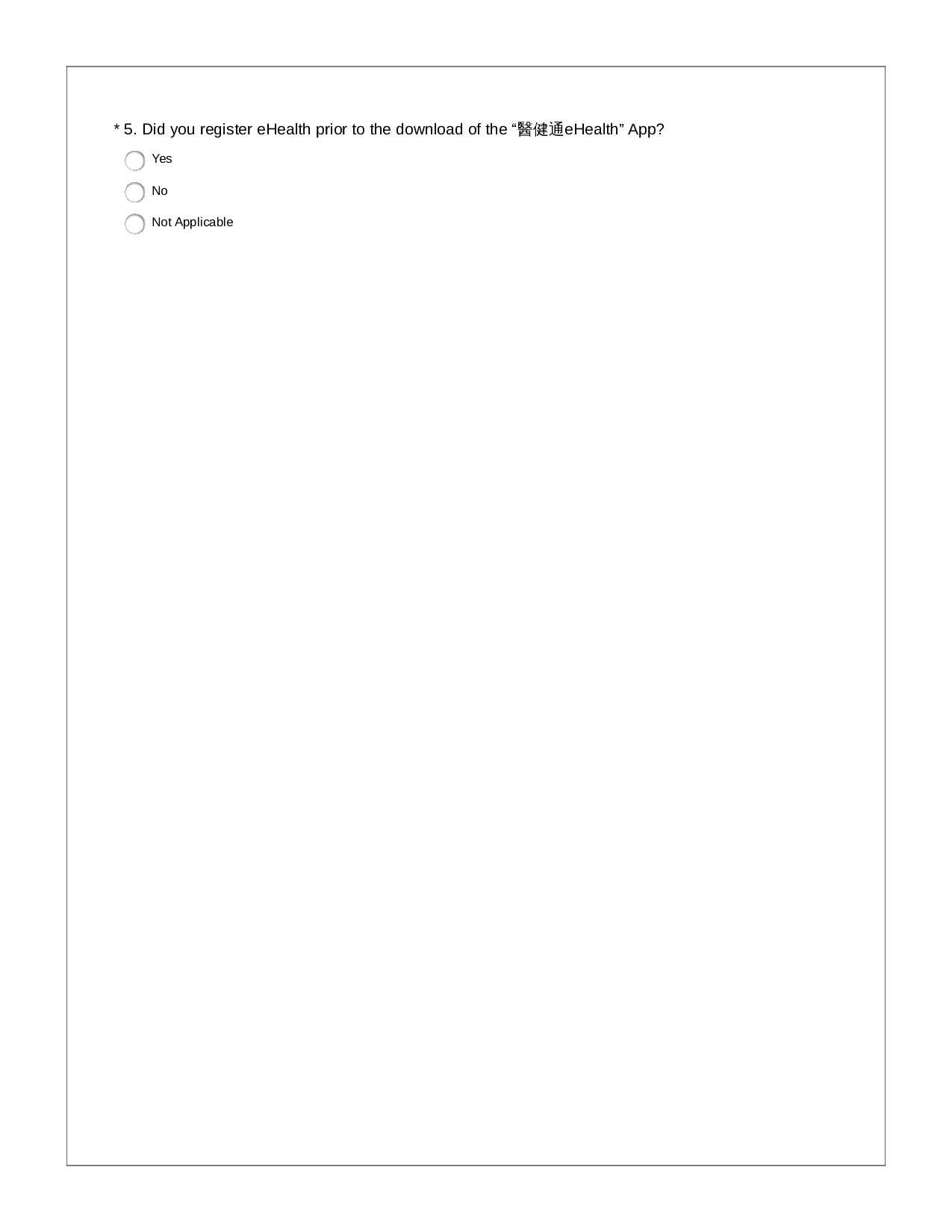

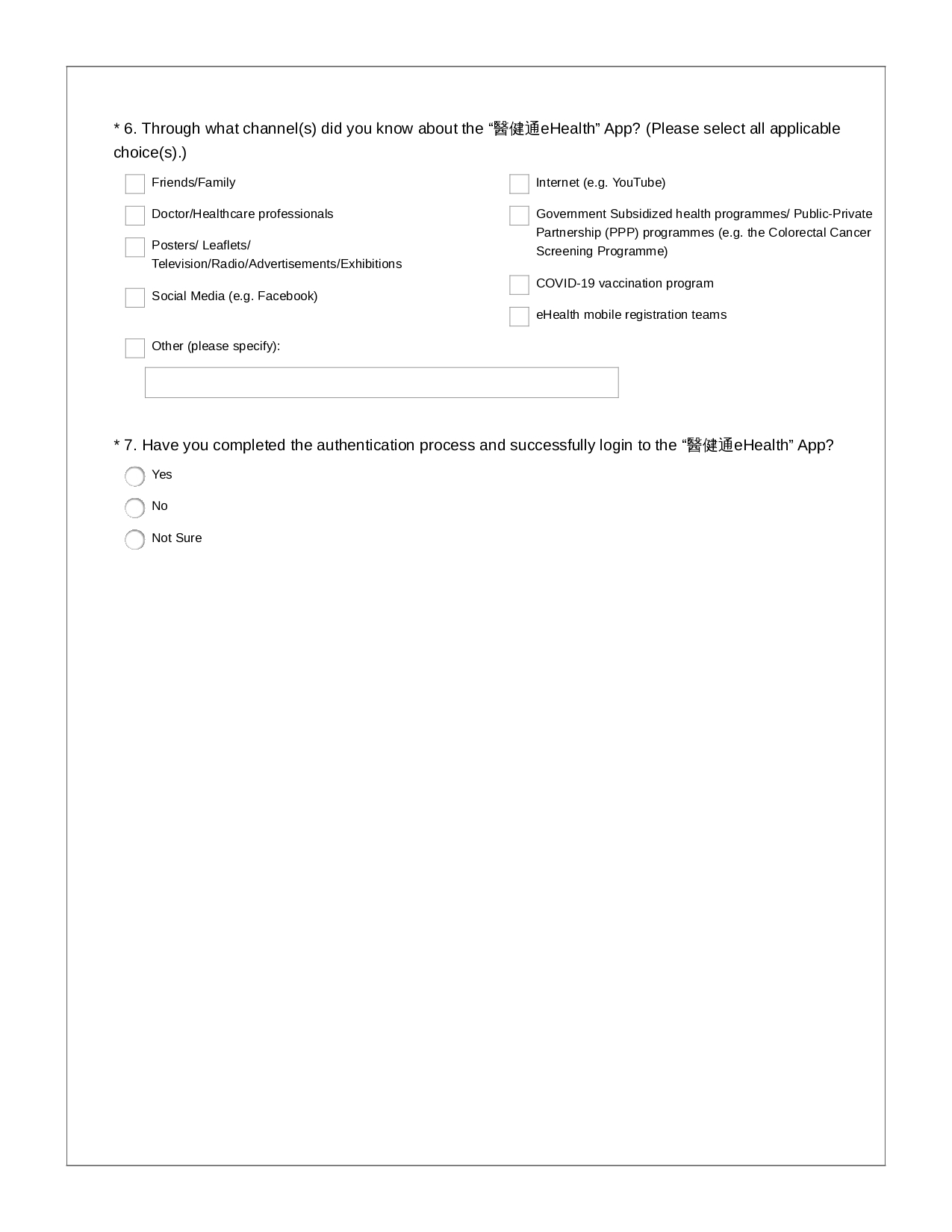

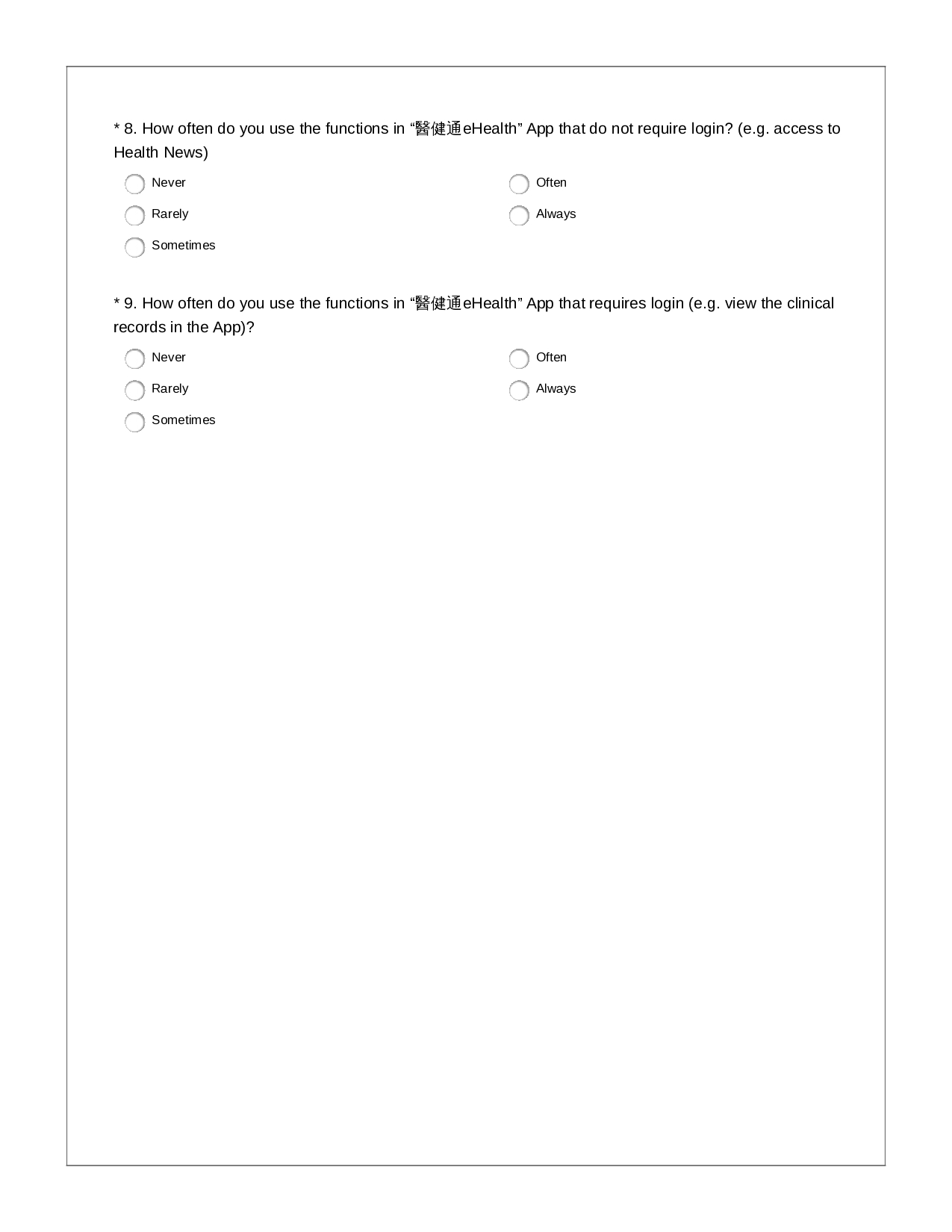

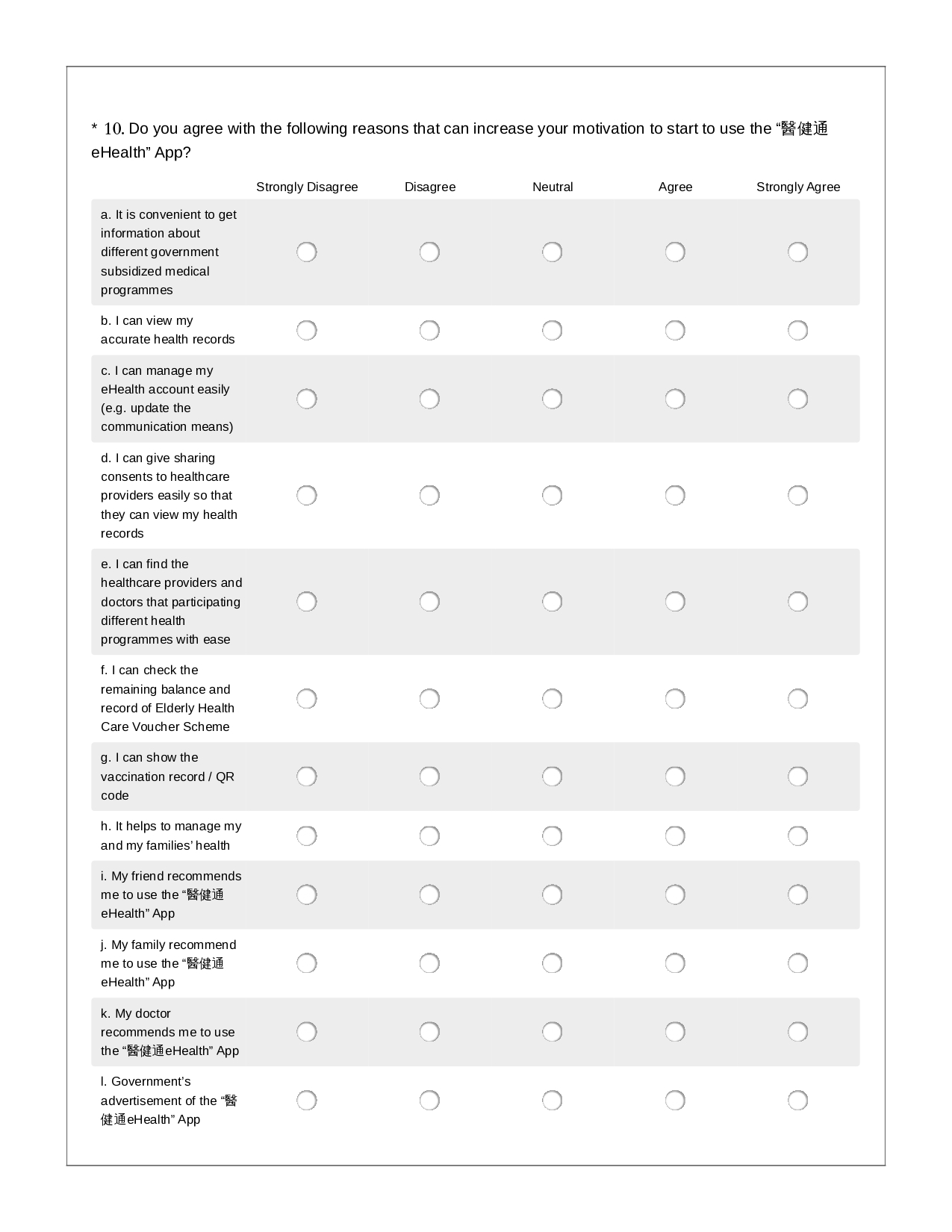

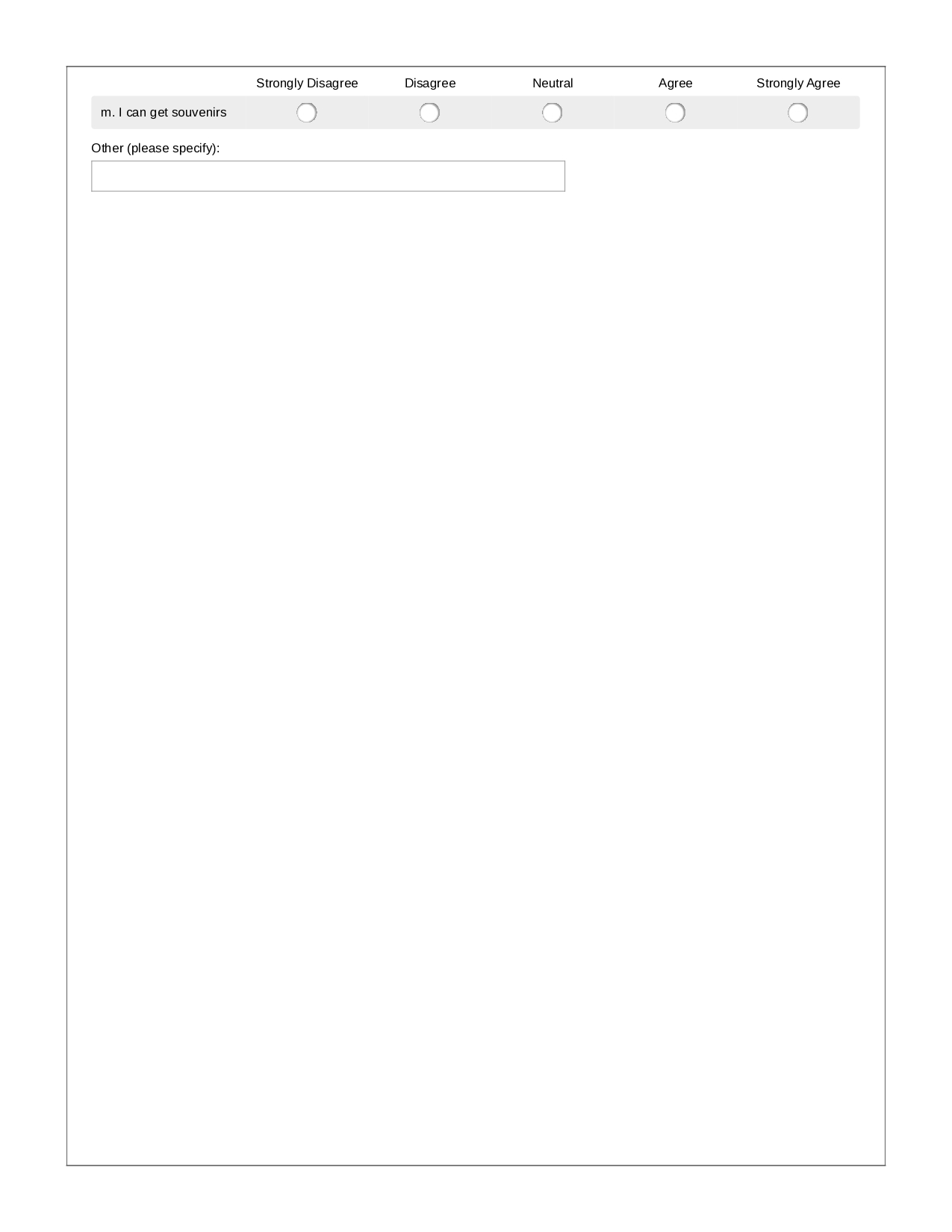

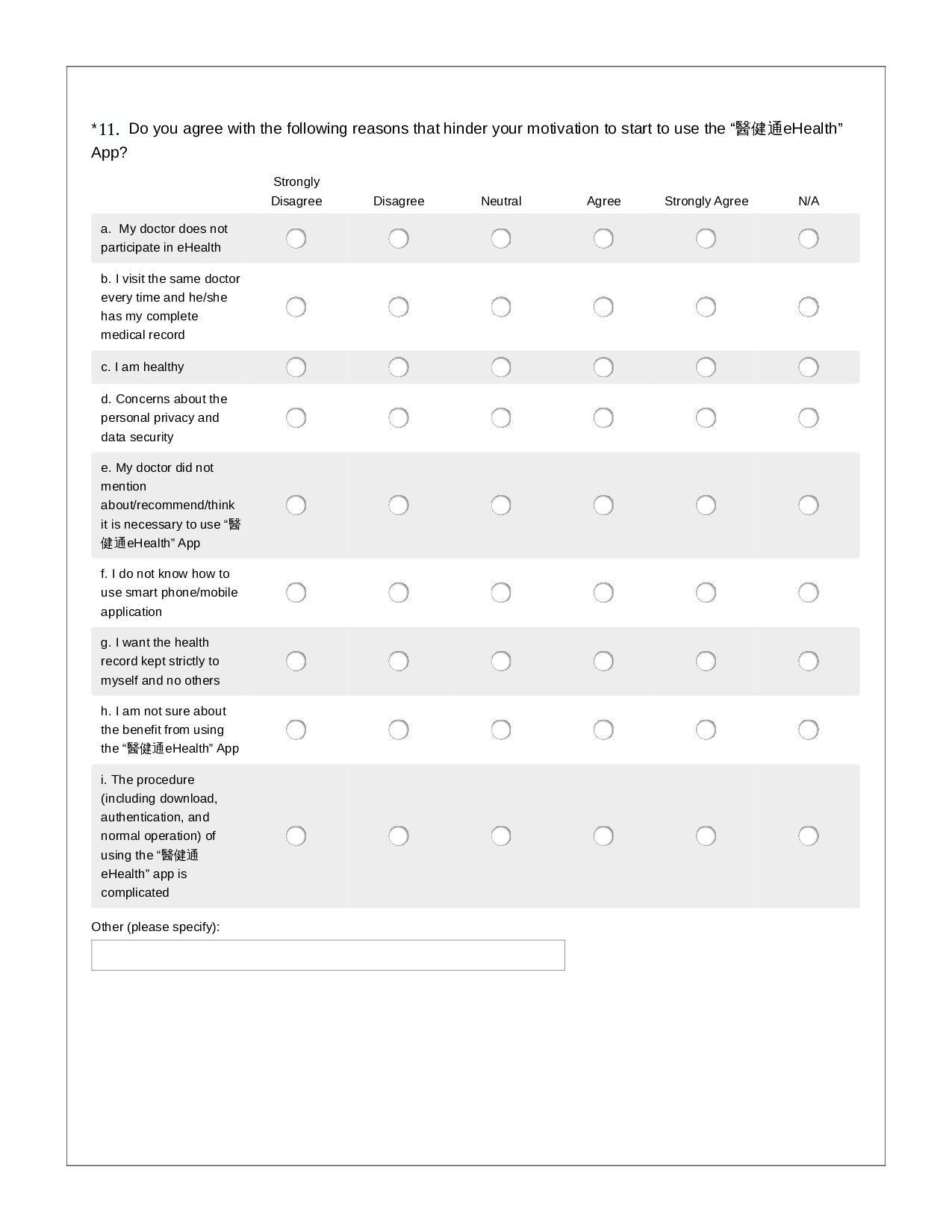

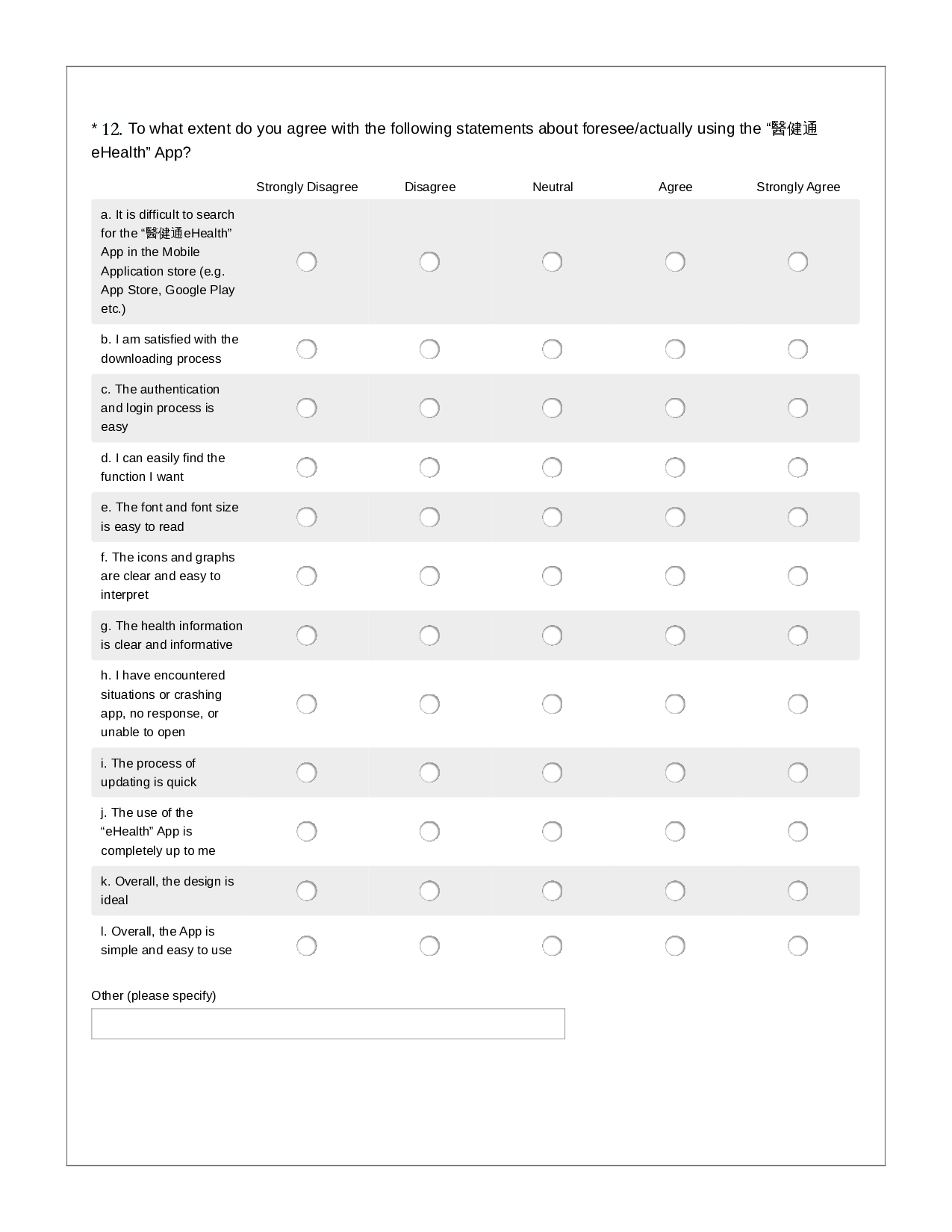

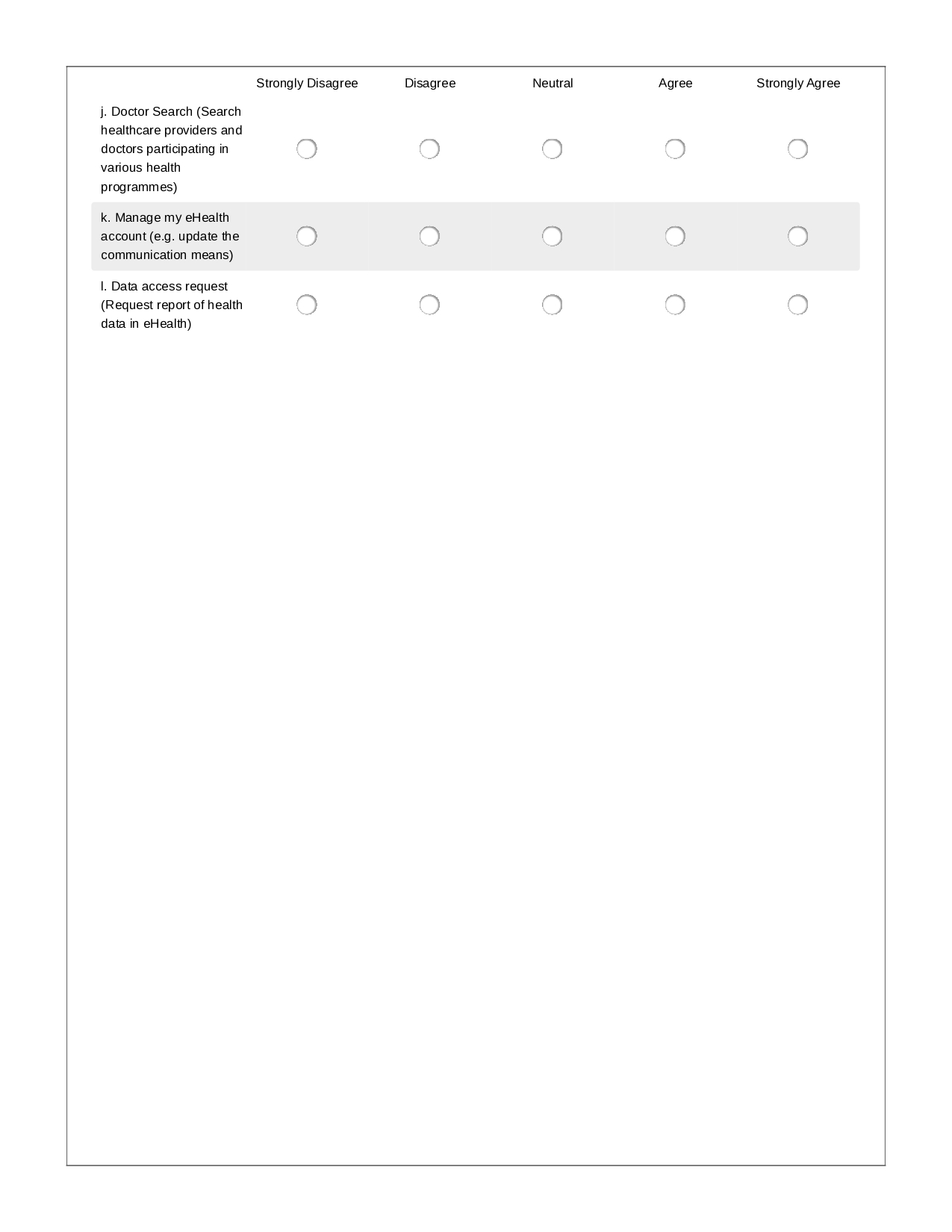

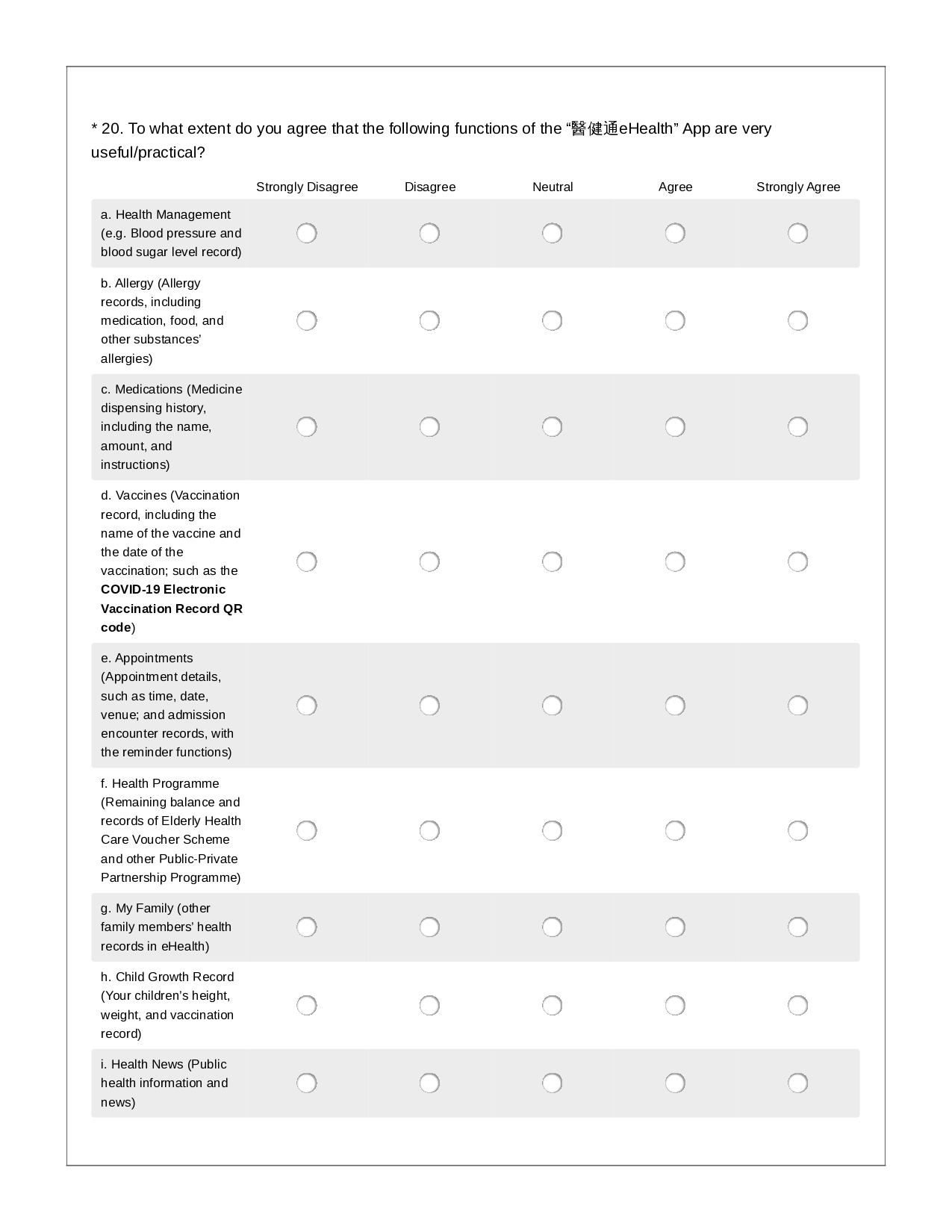

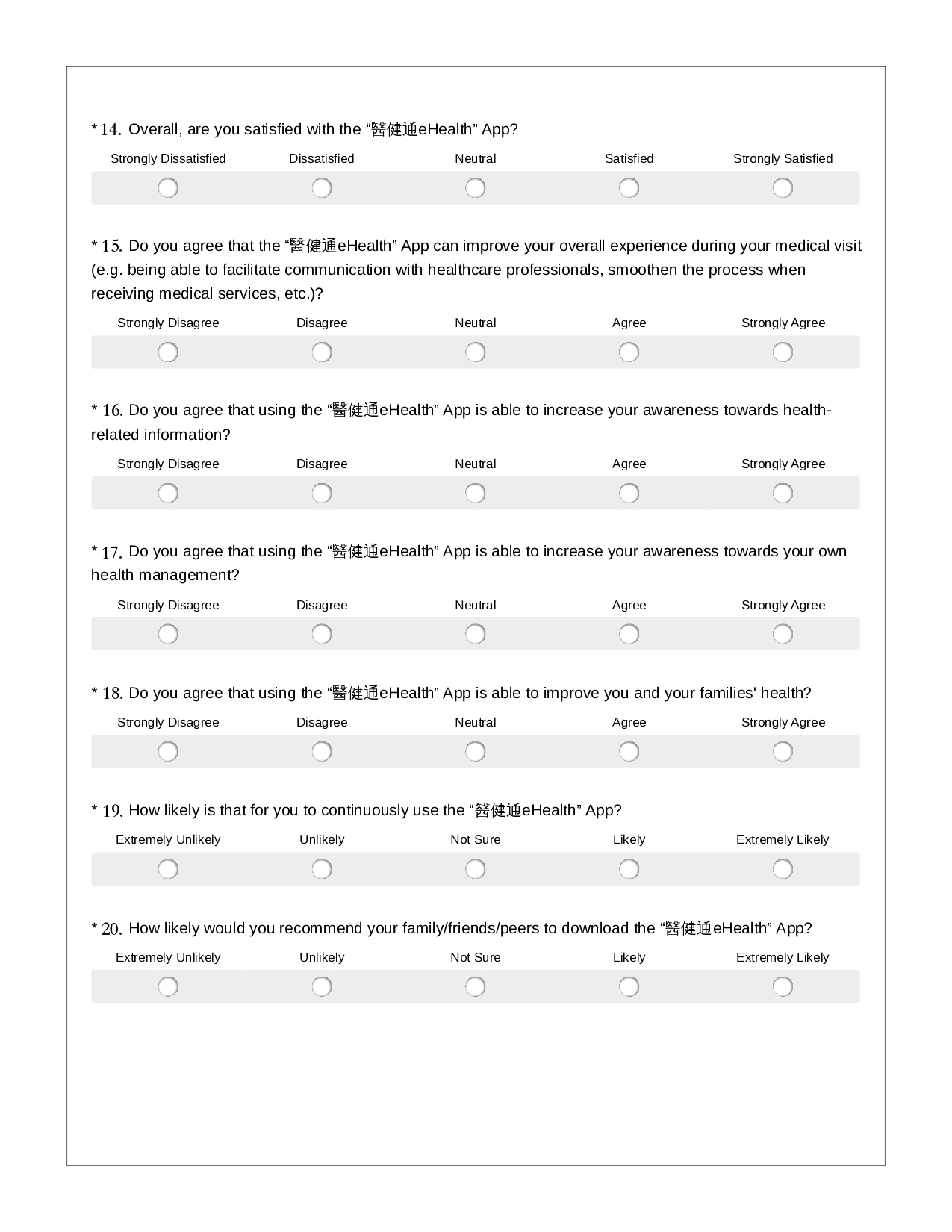

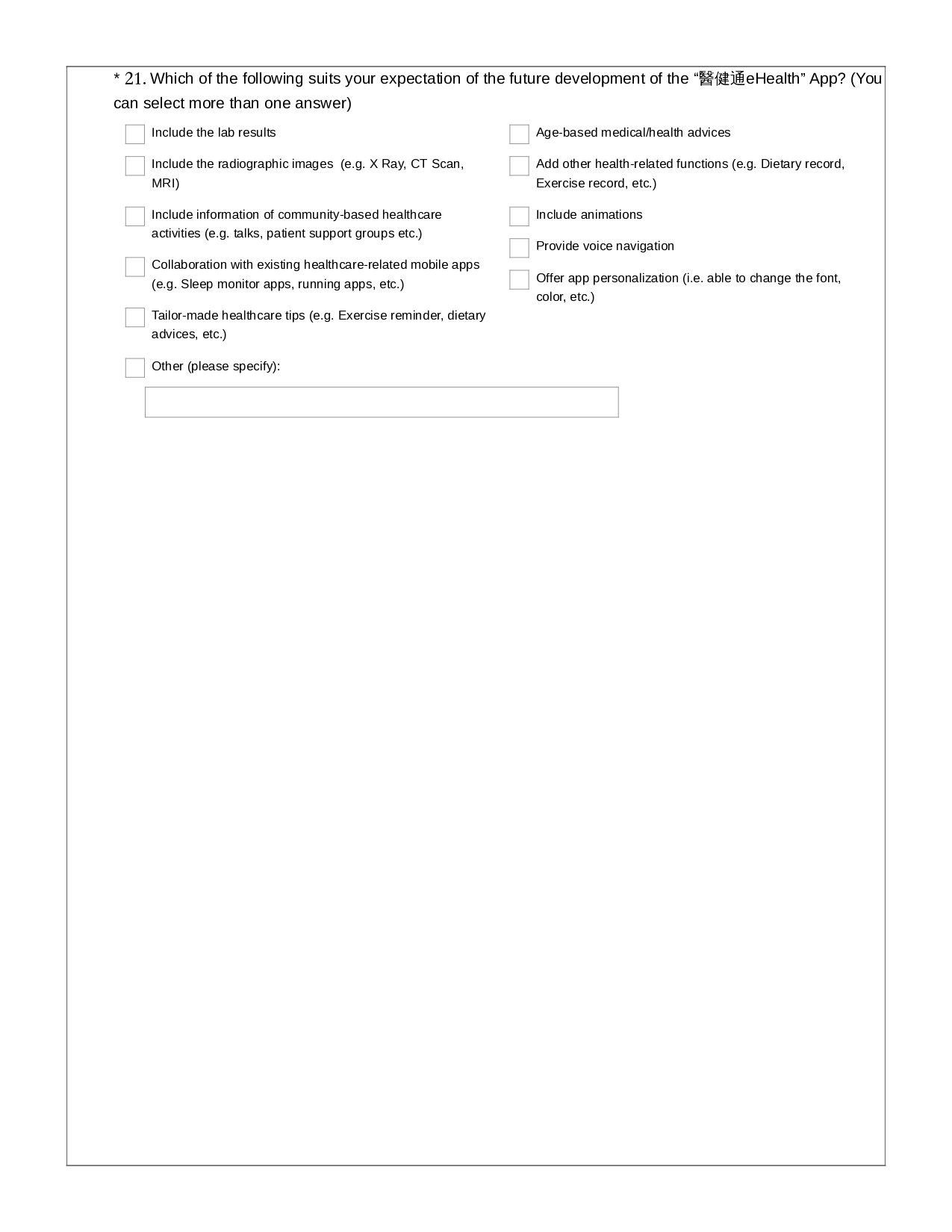

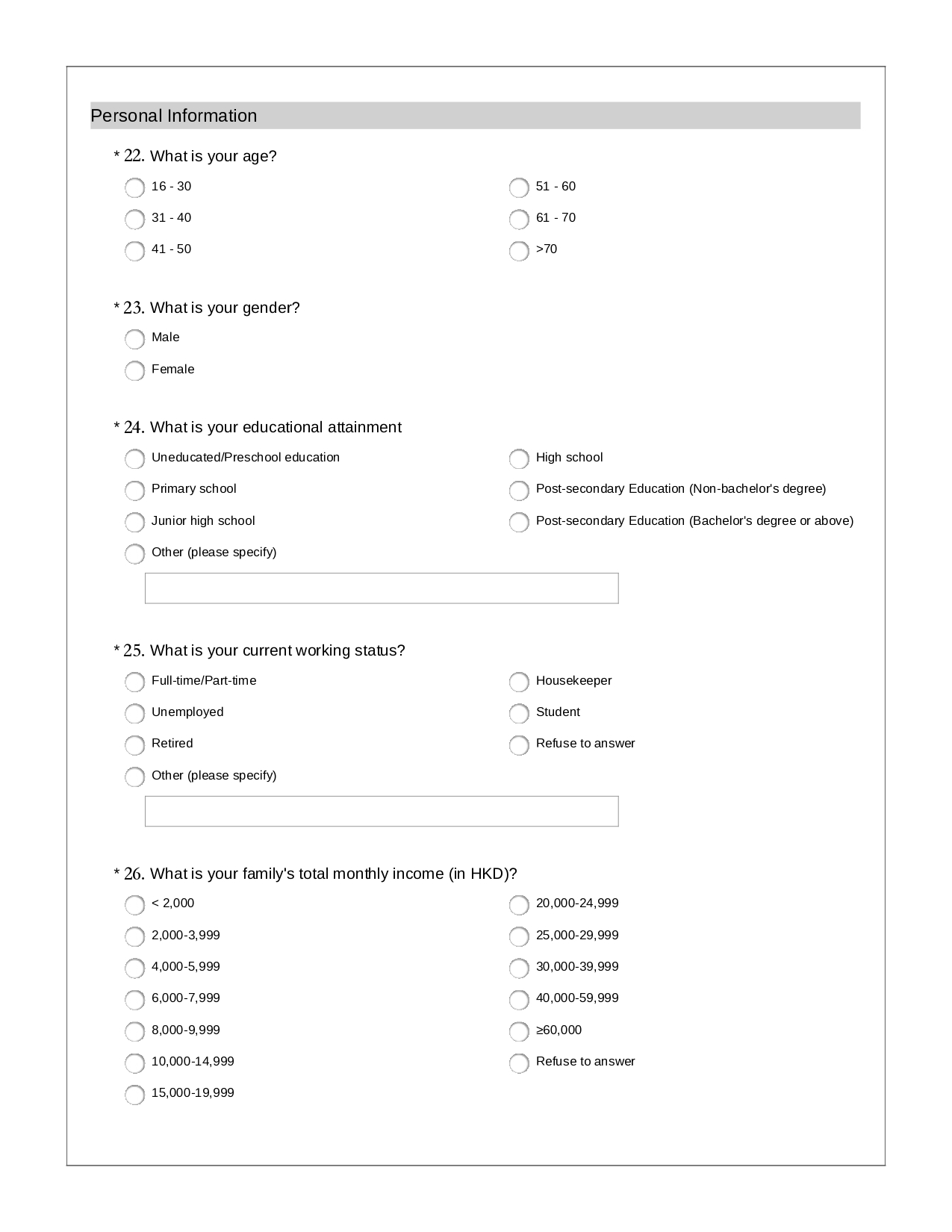

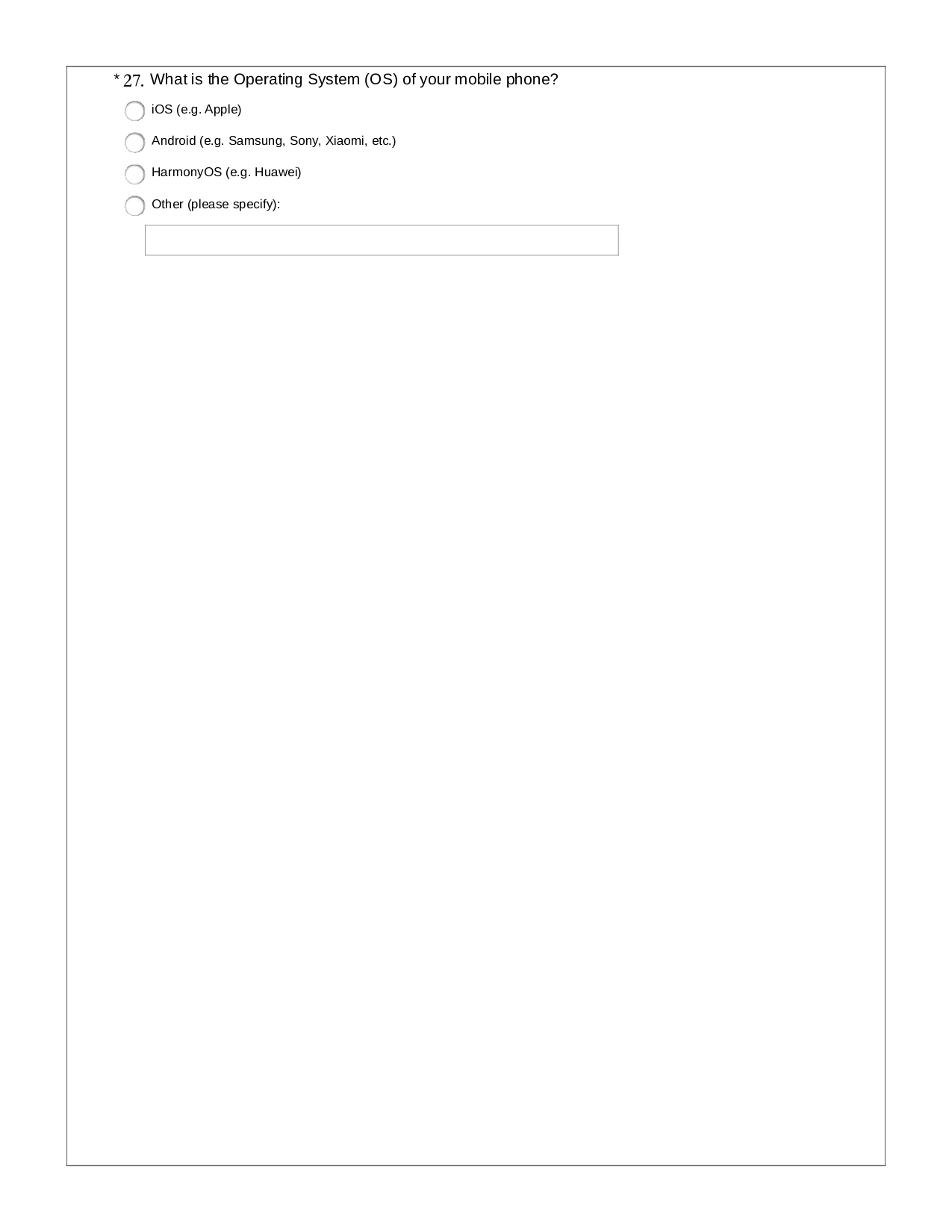
**

**Supplementary File 3 Survey for respondents have enrolled in eHRSS, but have not downloaded nor adopted the app
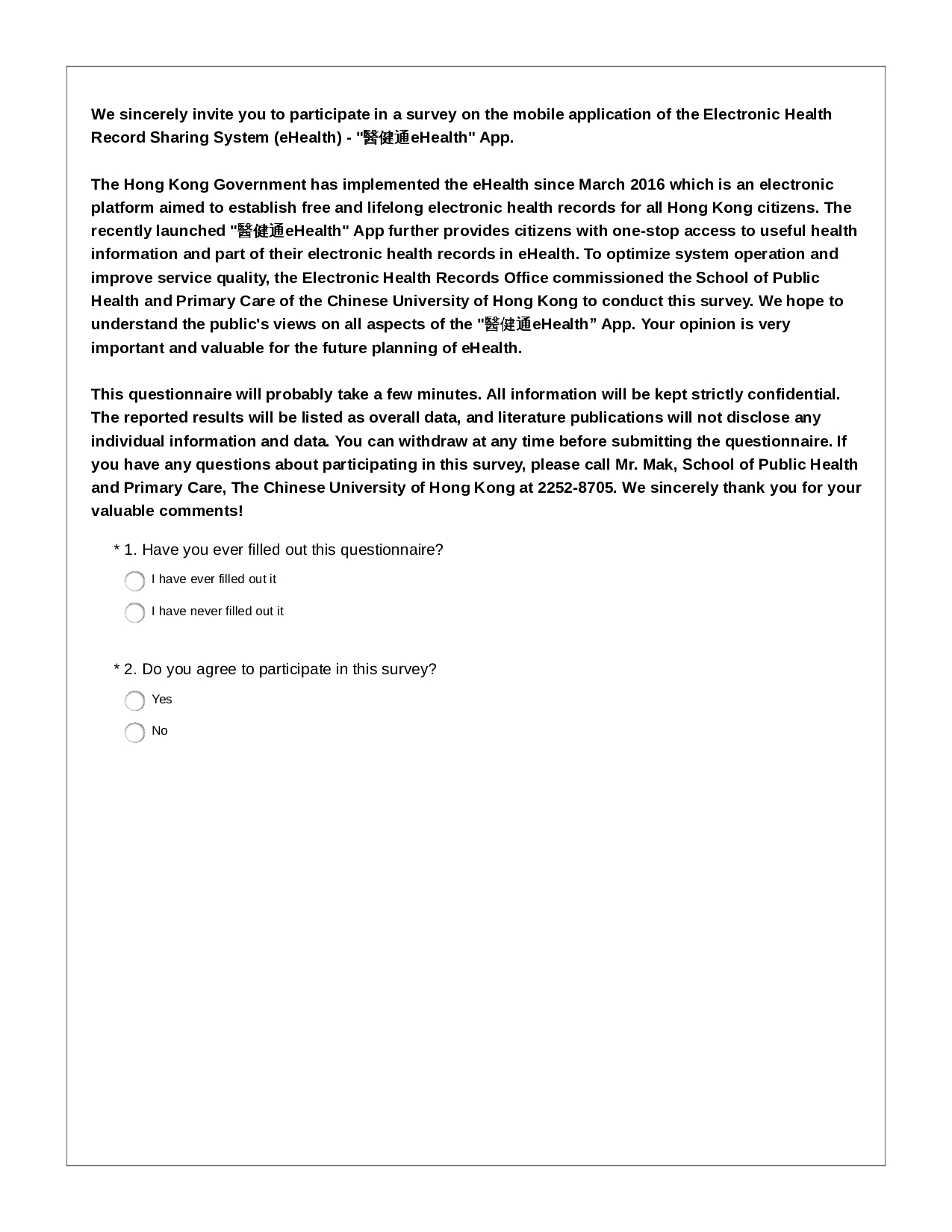

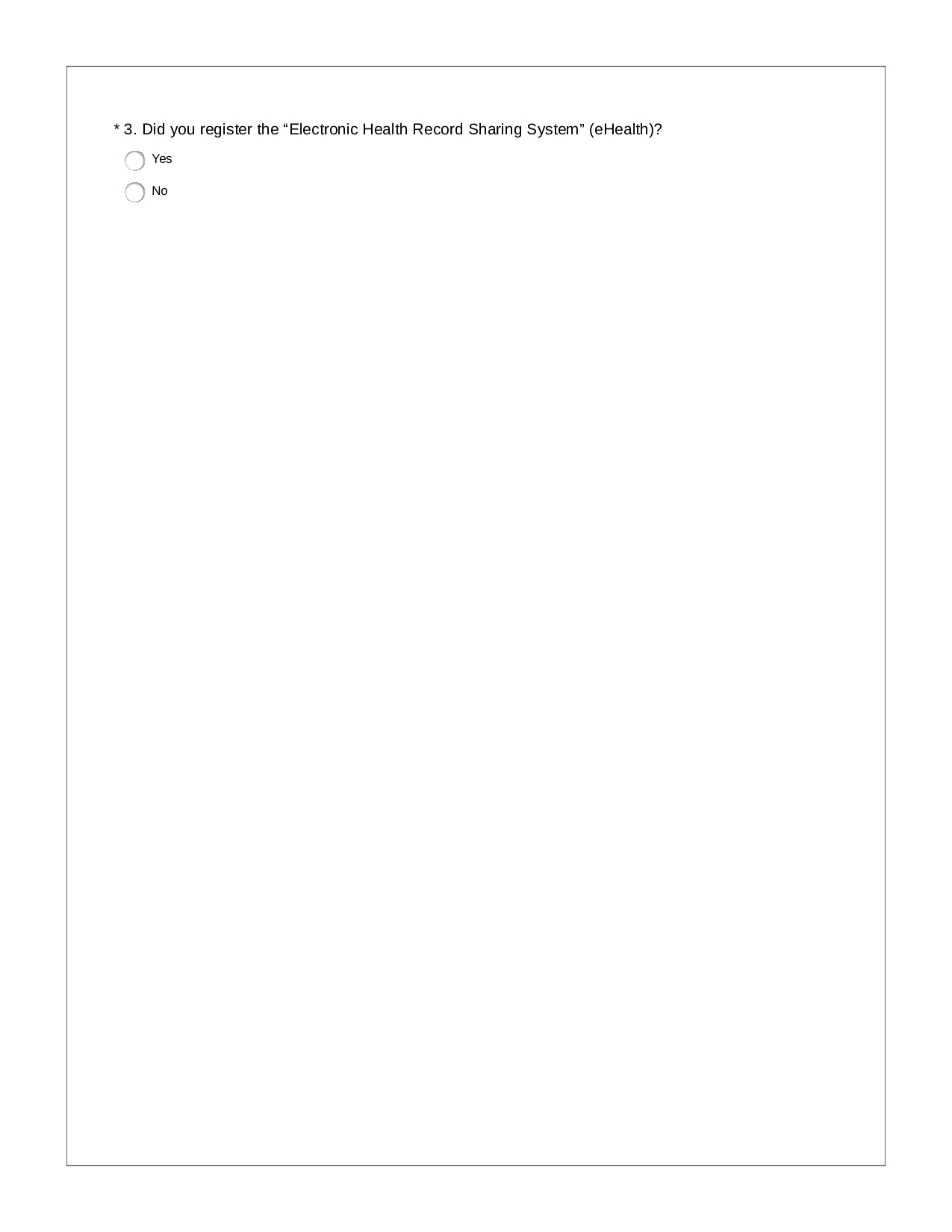

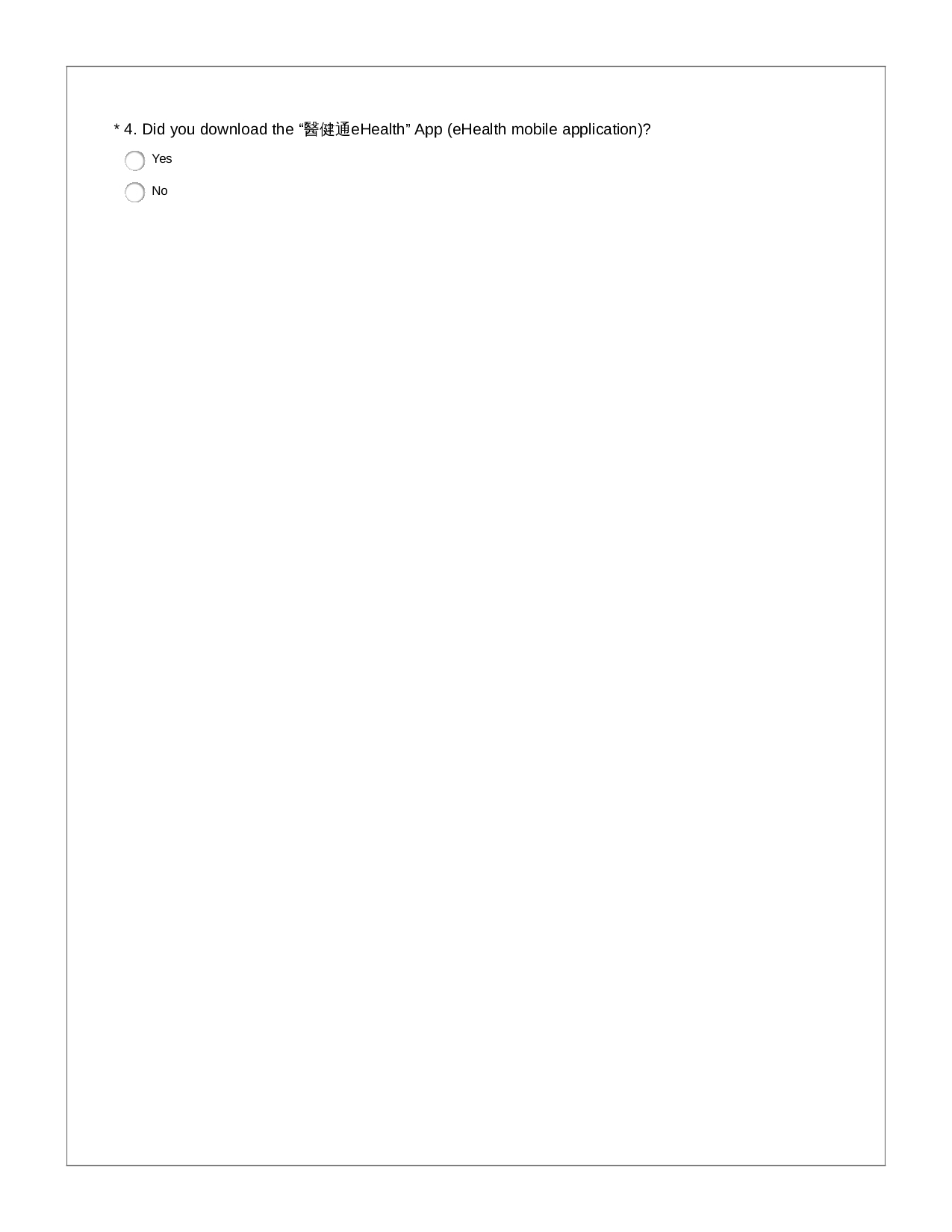

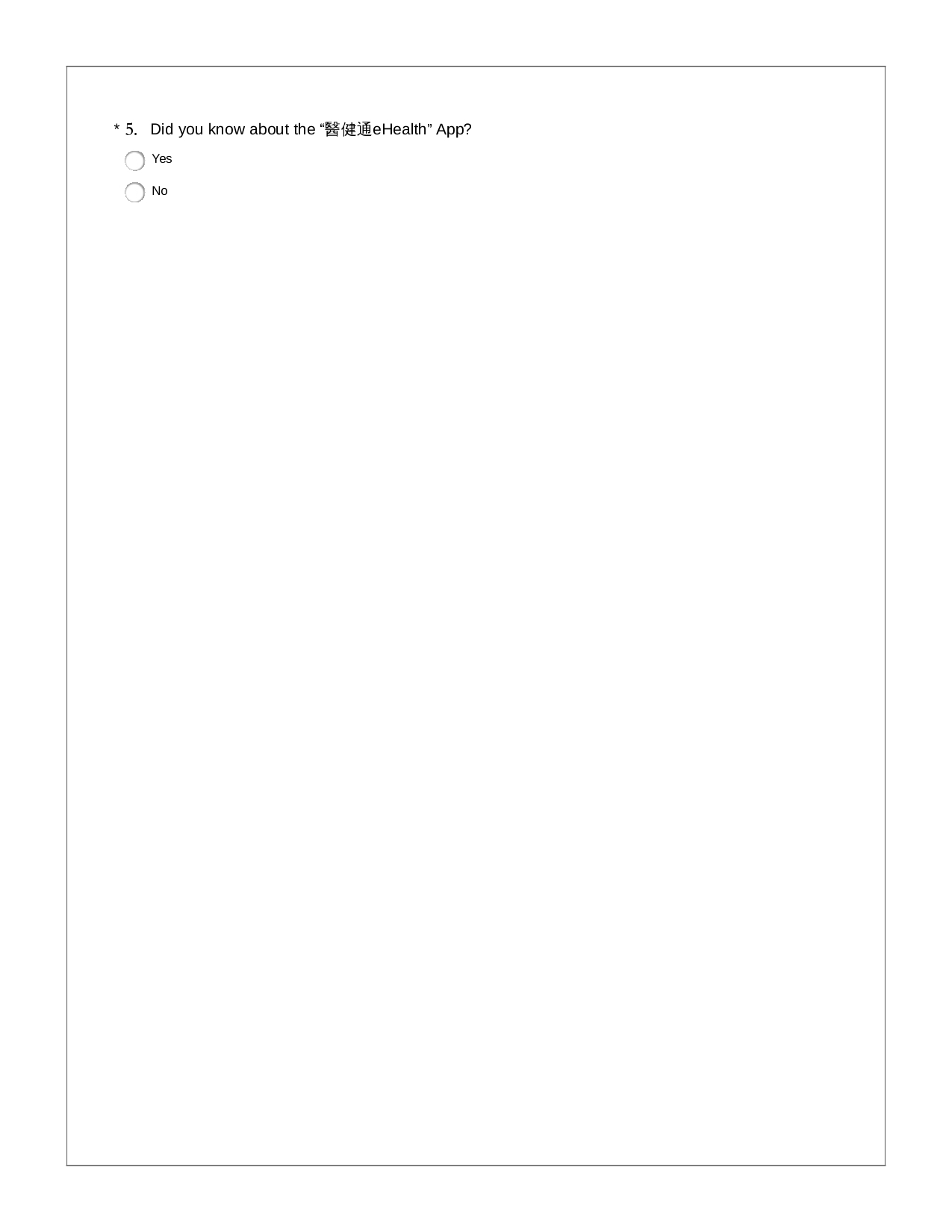

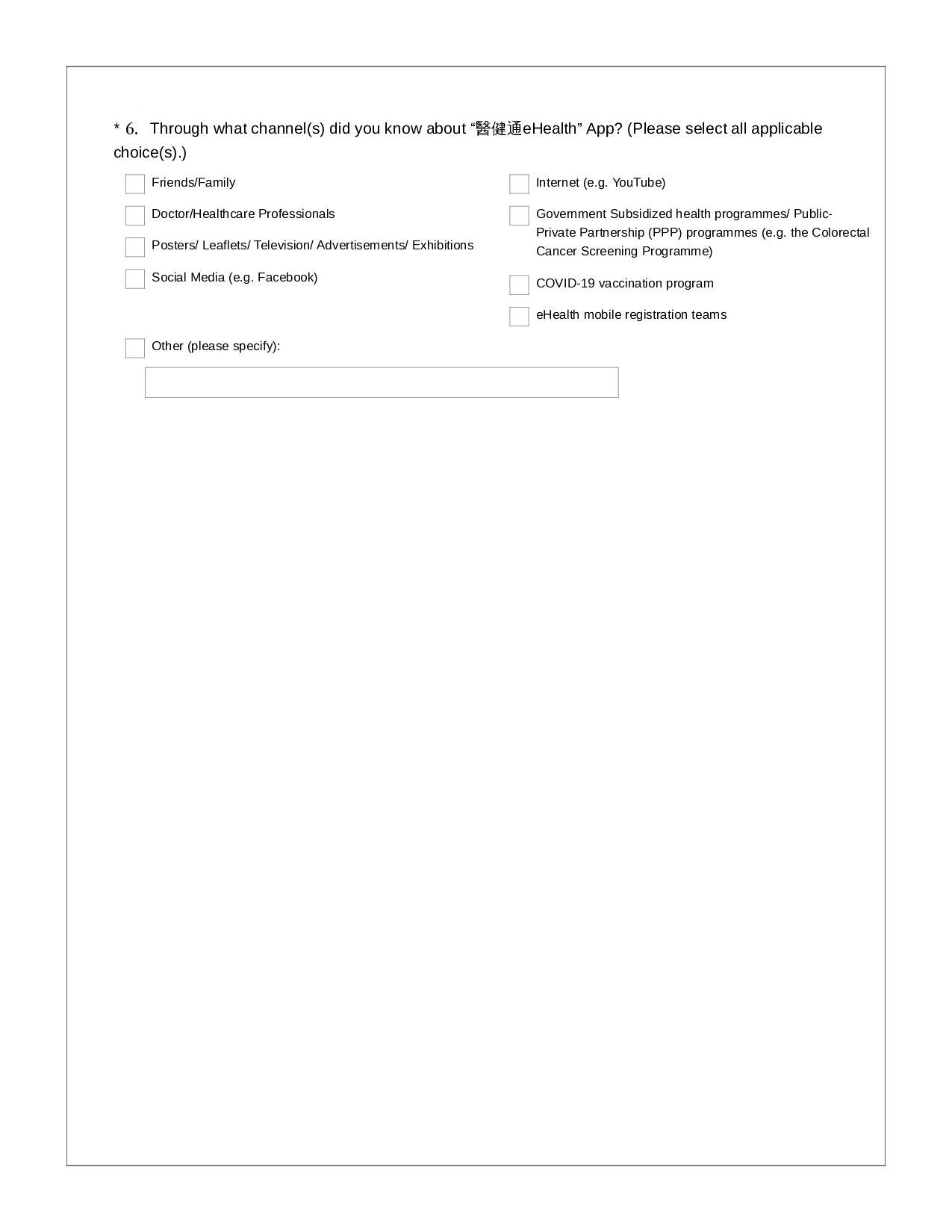

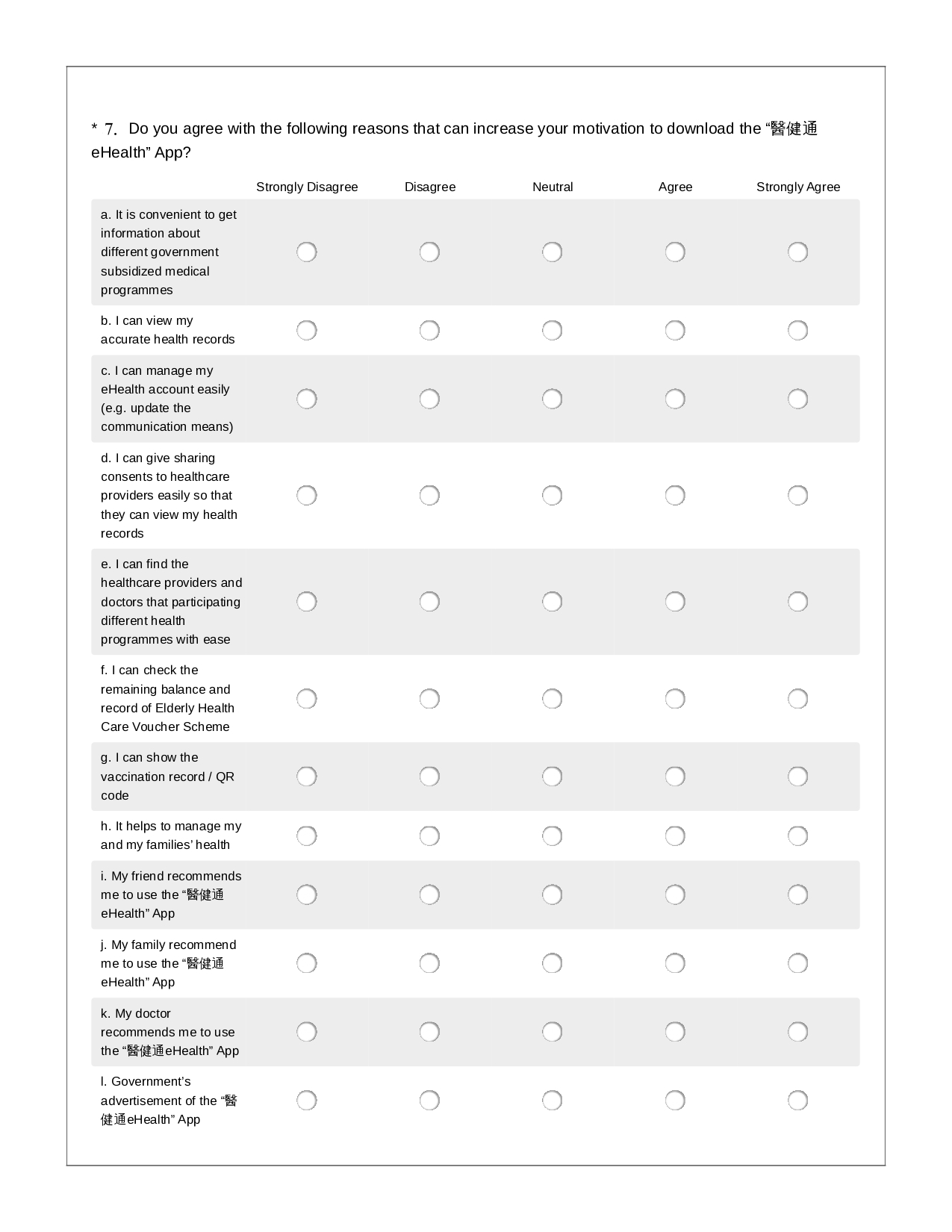

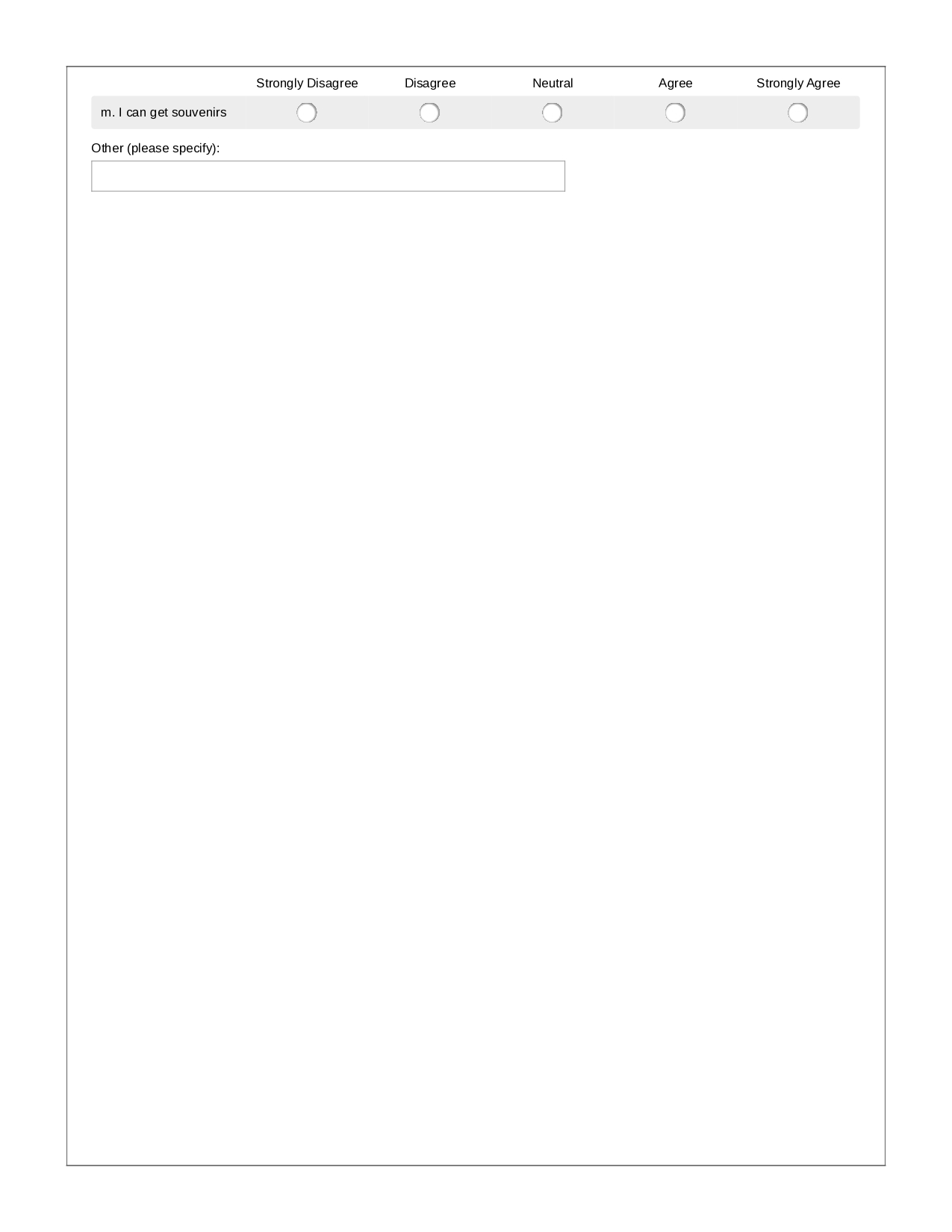

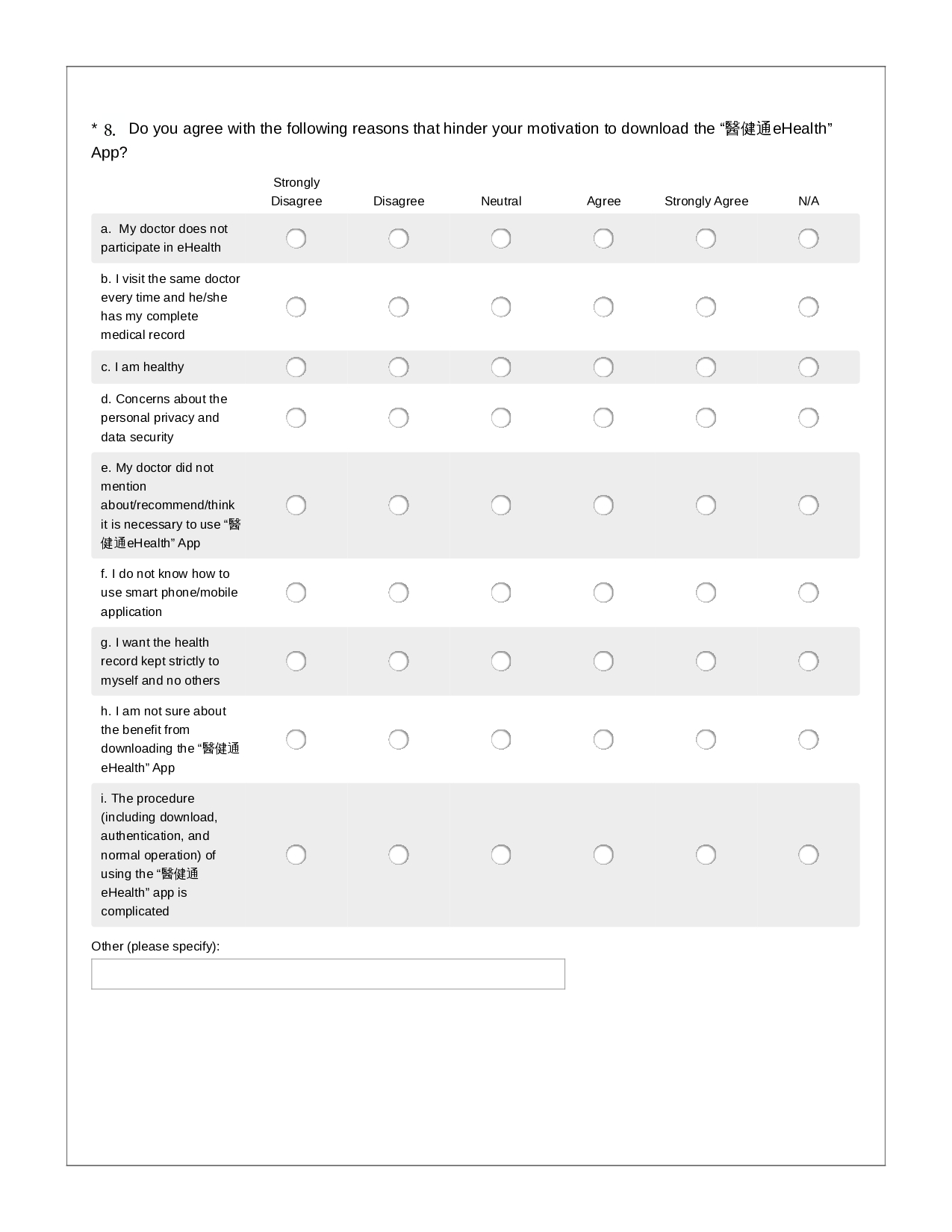

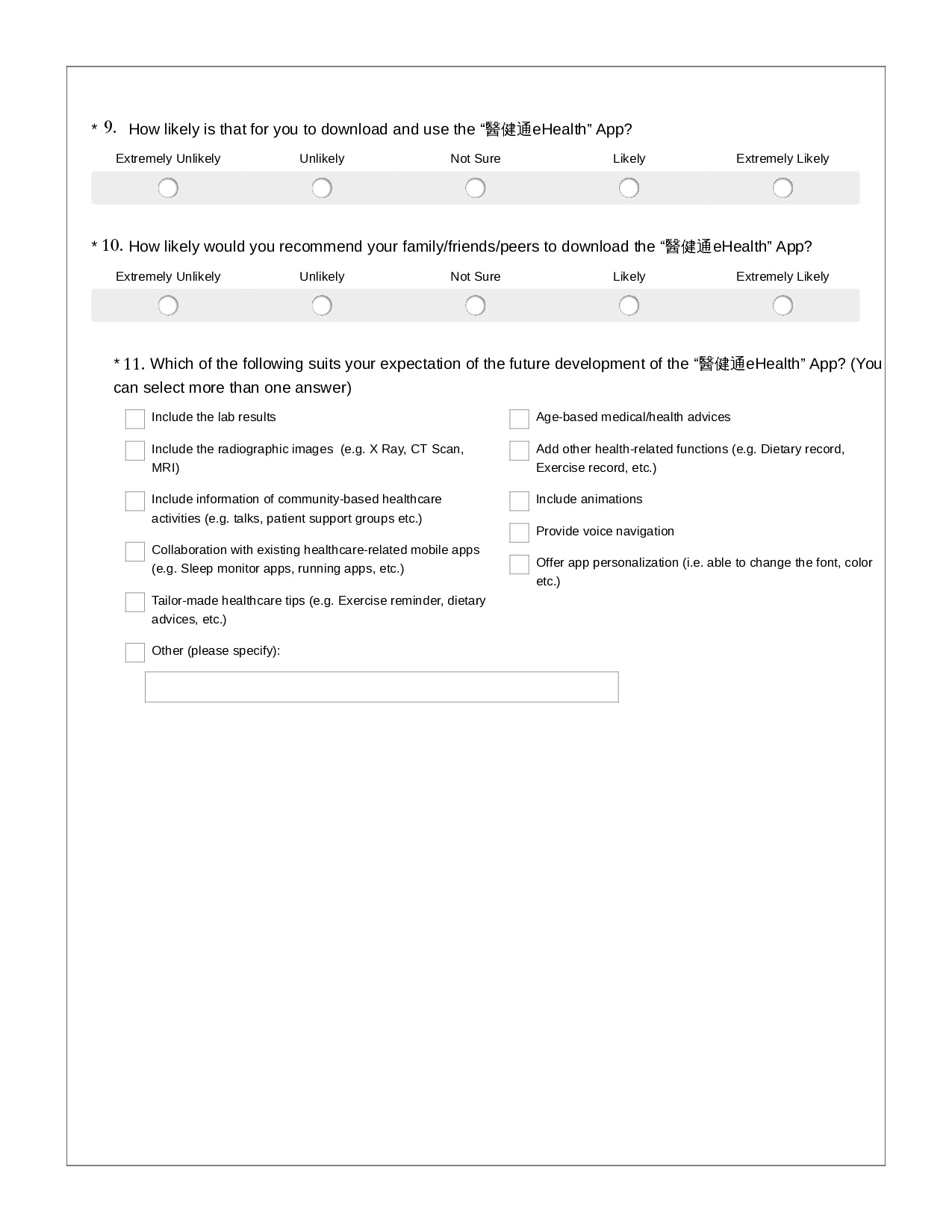

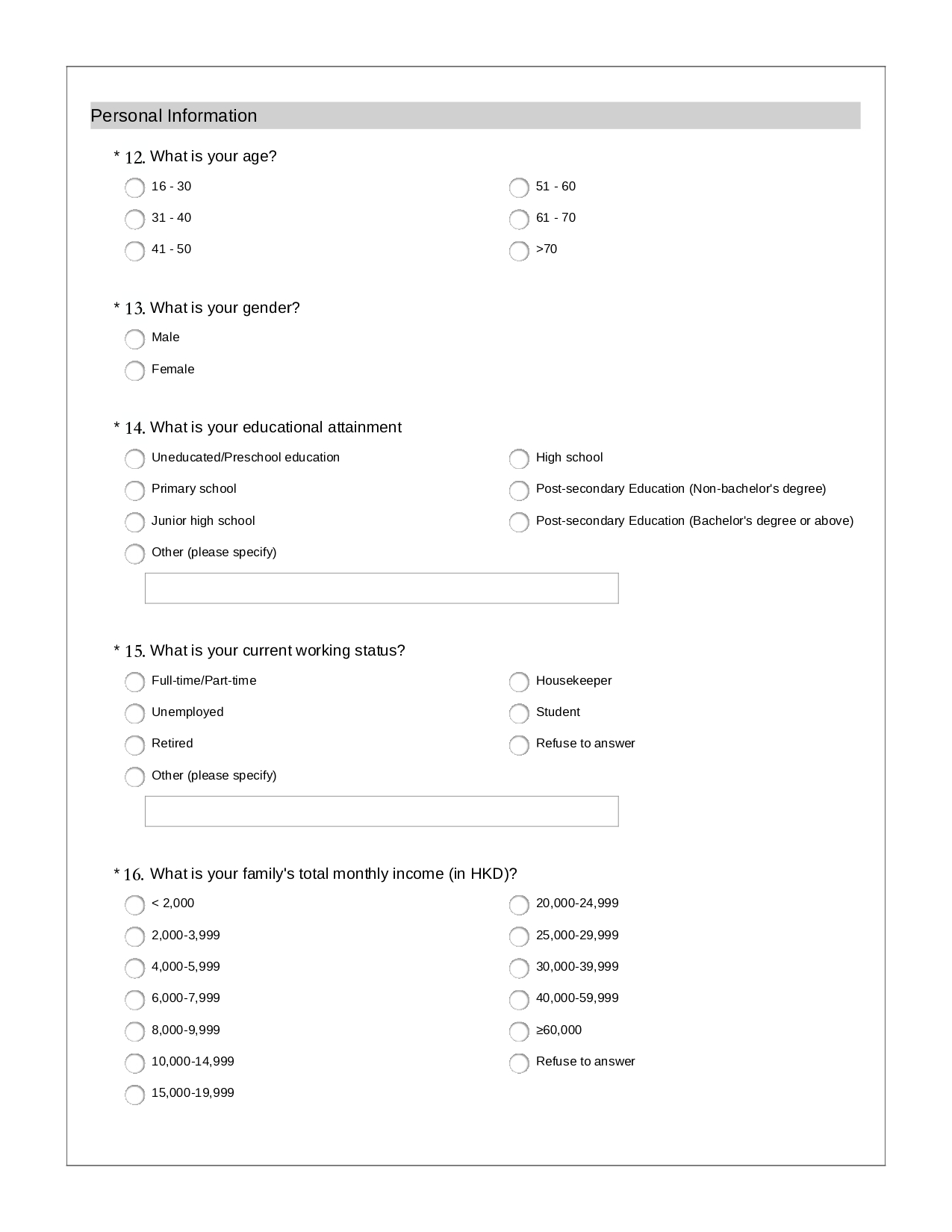

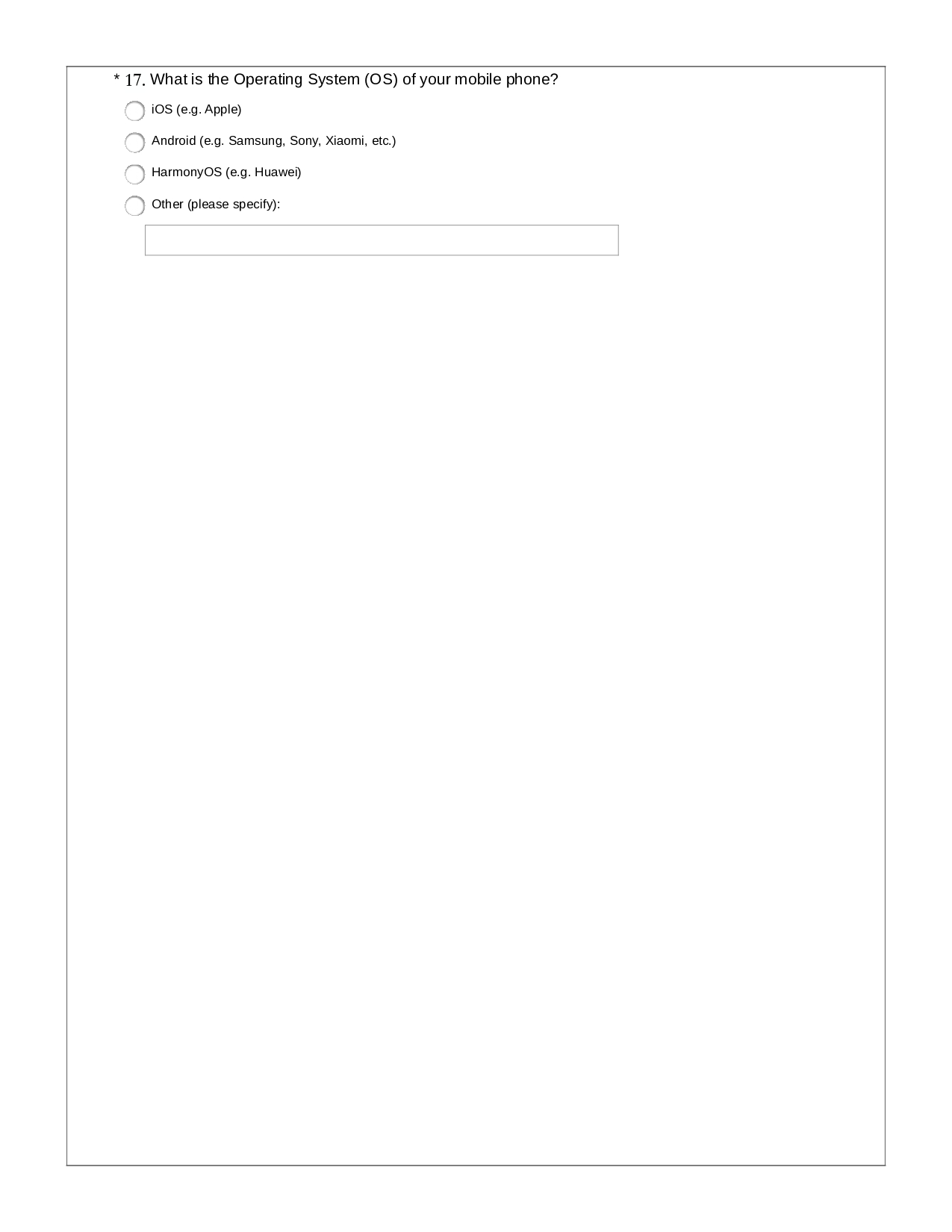
**
