# Supplementary material for: Factors Associated With the Acceptance of an eHealth App for Electronic Health Record Sharing System: Population-Based Study
Source: J Med Internet Res. 2022 Dec 12;24(12):e40370. doi: 10.2196/40370 (PMC9793296; doi:10.2196/40370)
Supplement: Multimedia Appendix 5 [file jmir_v24i12e40370_app5.docx]

|  | **Enrolled** | **Downloaded** | **Adopted** | **Total number** |
| --- | --- | --- | --- | --- |
|  | **in** | **eHealth app** | **eHealth app** | **(N=2,110)** |
|  | **eHRSS** |  |  |  |
| 1* | Yes | Yes | Yes | 1,242 |
| 2 | Yes | Yes | No | 275 |
| 3 | Yes | No | No | 203 |
| 4 | No | No | No | 266 |
| 5 | No | Yes | No | 124 |
