# Supplementary material for: Factors Associated With the Acceptance of an eHealth App for Electronic Health Record Sharing System: Population-Based Study
Source: J Med Internet Res. 2022 Dec 12;24(12):e40370. doi: 10.2196/40370 (PMC9793296; doi:10.2196/40370)
Supplement: Multimedia Appendix 6 [file jmir_v24i12e40370_app6.docx]

|  | **Downloaded and used eHealth app**  **(n=1242)** | | **Downloaded but not used eHealth app**  **(n=399)** | | **Not having downloaded and used eHealth app**  **(n=469)** | |
| --- | --- | --- | --- | --- | --- | --- |
|  | **n** | **%** | **n** | **%** | **n** | **%** |
| **Friends/ family members** | 234 | 18.8 | 78 | 19.5 | 76 | 16.2 |
| **Medical doctors** | 395 | 31.8 | 128 | 32.1 | 124 | 26.4 |
| **Publicity*** | 352 | 28.3 | 87 | 21.8 | 94 | 20.0 |
| **Social media** | 90 | 7.2 | 21 | 5.3 | 34 | 7.2 |
| **Websites** | 103 | 8.3 | 28 | 7.0 | 26 | 5.5 |
| **Government subsidized programmes**** | 114 | 9.2 | 23 | 5.8 | 31 | 6.6 |
| **COVID-19 vaccination programme** | 389 | 31.3 | 129 | 32.3 | 131 | 27.9 |
| **eHRSS registration station** | 224 | 18.0 | 62 | 15.5 | 37 | 7.9 |
| **Others** | 35 | 2.8 | 18 | 4.5 | 8 | 1.7 |

eHRSS: electronic Health Record Sharing System

*Publicity includes posters, pamphlets, television, outdoor advertisement

**These include public private partnership and government initiated programmes
