# Supplementary material for: Factors Associated With the Acceptance of an eHealth App for Electronic Health Record Sharing System: Population-Based Study
Source: J Med Internet Res. 2022 Dec 12;24(12):e40370. doi: 10.2196/40370 (PMC9793296; doi:10.2196/40370)
Supplement: Multimedia Appendix 7 [file jmir_v24i12e40370_app7.docx]

|  | **Downloaded and used eHealth app**  **(n=1242)** | | **Downloaded but not used eHealth app**  **(n=399)** | |
| --- | --- | --- | --- | --- |
|  | **n** | **strongly agree or agree (%)** | **n** | **strongly agree or agree (%)** |
| **Downloading processes** | | | | |
| Difficult to search the downloading function | 157 | 12.6 | 94 | 23.6 |
| Satisfied about the downloading processes | 908 | 73.1 | 239 | 59.9 |
| **Adoption processes** | | | | |
| Smooth login process | 788 | 63.4 | 158 | 39.6 |
| I can easily find the function I want | 755 | 60.8 | 144 | 36.1 |
| Easy to read the fonts and size of the words | 947 | 76.2 | 242 | 60.7 |
| Easy to understand the icons and Tables | 880 | 70.9 | 190 | 47.6 |
| Clear message | 772 | 62.2 | 164 | 41.1 |
| Frequent encounter of technical errors | 181 | 14.6 | 106 | 26.6 |
| Quick updating process | 788 | 63.4 | 172 | 43.1 |
| Overall, satisfactory design | 781 | 62.9 | 171 | 42.9 |
| Overall, easy to use | 869 | 70.0 | 158 | 39.6 |

|  | **Downloaded and used eHealth app (n=1242)** | | | **Downloaded but not used eHealth app (n=399)** | | | |
| --- | --- | --- | --- | --- | --- | --- | --- |
|  | **n** | **Mean (SD)** | **95% CI** | | **n** | **Mean (SD)** | **95% CI** |
| **Downloading processes** | | | | | | | |
| Difficult to search the downloading function | 157 | 2.36 (0.92) | 3.66-3.86 | | 94 | 2.77 (0.96) | 2.68-2.87 |
| Satisfied about the downloading processes | 908 | 3.79 (0.76) | 2.31-2.41 | | 239 | 3.48 (0.84) | 3.40-3.57 |
| **Adoption processes** | | | | | | | |
| Smooth login process | 788 | 3.58 (0.89) | 3.53-3.63 | | 158 | 3.10 (0.99) | 3.00-3.20 |
| I can easily find the function I want | 755 | 3.57 (0.83) | 3.53-3.62 | | 144 | 3.15 (0.89) | 3.06-3.24 |
| Easy to read the fonts and size of the words | 947 | 3.83 (0.65) | 3.80-3.87 | | 242 | 3.60 (0.66) | 3.53-3.66 |
| Easy to understand the icons and Tables | 880 | 3.75 (0.70) | 3.71-3.79 | | 190 | 3.40 (0.77) | 3.33-3.48 |
| Clear message | 772 | 3.63 (0.76) | 3.59-3.68 | | 164 | 3.31 (0.79) | 3.23-3.39 |
| Frequent encounter of technical errors | 181 | 2.56 (0.90) | 2.51-2.61 | | 106 | 3.04 (0.82) | 2.95-3.12 |
| Quick updating process | 788 | 3.50 (0.71) | 3.46-3.54 | | 172 | 3.25 (0.71) | 3.18-3.32 |
| Overall, satisfactory design | 781 | 3.63 (0.78) | 3.59-3.68 | | 171 | 3.28 (0.87) | 3.20-3.37 |
| Overall, easy to use | 869 | 3.73 (0.75) | 3.68-3.77 | | 158 | 3.27 (0.85) | 3.18-3.35 |
