# Supplementary material for: Factors Associated With the Acceptance of an eHealth App for Electronic Health Record Sharing System: Population-Based Study
Source: J Med Internet Res. 2022 Dec 12;24(12):e40370. doi: 10.2196/40370 (PMC9793296; doi:10.2196/40370)
Supplement: Multimedia Appendix 8 [file jmir_v24i12e40370_app8.docx]

|  | **Downloaded and used eHealth app**  **(n=1242)** | | **Downloaded but not used eHealth app**  **(n=135*)** | |
| --- | --- | --- | --- | --- |
|  | **n** | **strongly agree or agree (%)** | **n** | **strongly agree or agree (%)** |
| Health management | 786 | 63.3 | 66 | 48.9 |
| Allergy records | 924 | 74.4 | 83 | 61.5 |
| Medication records | 1015 | 81.7 | 80 | 59.3 |
| Vaccine records | 1108 | 89.2 | 107 | 79.3 |
| Appointment records | 1055 | 84.9 | 97 | 71.9 |
| Health programmes | 846 | 68.1 | 77 | 57.0 |
| My Family (other family members’ health records in eHealth) | 600 | 48.3 | 53 | 39.3 |
| Child Growth Record (Your children’s height, weight, and vaccination record) | 555 | 44.7 | 44 | 32.6 |
| Obtaining public health information and news | 754 | 60.7 | 67 | 49.6 |
| Search of doctors | 665 | 53.5 | 66 | 48.9 |
| Management of one’s eHealth records | 737 | 59.3 | 55 | 40.7 |
| Data access request (Request report of health data in eHealth) | 715 | 57.6 | 58 | 43.0 |

|  | **Downloaded and used eHealth app (n=1242)** | | | **Downloaded but not used eHealth app (n=135*)** | | |
| --- | --- | --- | --- | --- | --- | --- |
|  | **n** | **Mean (SD)** | **95% CI** | **n** | **Mean (SD)** | **95% CI** |
| Health management | 786 | 3.70 (0.82) | 3.66-3.75 | 66 | 3.51 (0.82) | 3.37-3.65 |
| Allergy records | 924 | 3.90 (0.74) | 3.86-3.94 | 83 | 3.68 (0.72) | 3.56-3.80 |
| Medication records | 1015 | 4.02 (0.74) | 3.97-4.06 | 80 | 3.65 (0.84) | 3.51-3.79 |
| Vaccine records | 1108 | 4.21 (0.70) | 4.17-4.24 | 107 | 3.97 (0.75) | 3.84-4.10 |
| Appointment records | 1055 | 4.12 (0.76) | 4.07-4.16 | 97 | 3.84 (0.82) | 3.71-3.98 |
| Health programmes | 846 | 3.85 (0.76) | 3.80-3.89 | 77 | 3.66 (0.69) | 3.54-3.78 |
| My Family (other family members’ health records in eHealth) | 600 | 3.52 (0.80) | 3.48-3.57 | 53 | 3.41 (0.76) | 3.28-3.54 |
| Child Growth Record (Your children’s height, weight, and vaccination record) | 555 | 3.48 (0.74) | 3.44-3.52 | 44 | 3.31 (0.74) | 3.19-3.44 |
| Obtaining public health information and news | 754 | 3.66 (0.74) | 3.61-3.70 | 67 | 3.50 (0.70) | 3.38-3.62 |
| Search of doctors | 665 | 3.57 (0.76) | 3.53-3.62 | 66 | 3.50 (0.72) | 3.37-3.62 |
| Management of one’s eHealth records | 737 | 3.66 (0.69) | 3.62-3.69 | 55 | 3.38 (0.75) | 3.25-3.51 |
| Data access request (Request report of health data in eHealth) | 715 | 3.61 (0.82) | 3.56-3.65 | 58 | 3.39 (0.86) | 3.25-3.54 |

*164 respondents did not answer the questions in either the English or Chinese version of the survey
