# Supplementary material for: Factors Associated With the Acceptance of an eHealth App for Electronic Health Record Sharing System: Population-Based Study
Source: J Med Internet Res. 2022 Dec 12;24(12):e40370. doi: 10.2196/40370 (PMC9793296; doi:10.2196/40370)
Supplement: Multimedia Appendix 9 [file jmir_v24i12e40370_app9.docx]

|  | **Downloaded and used eHealth app**  **(n=1242)** | | **Downloaded but not used eHealth app**  **(n=399)** | |
| --- | --- | --- | --- | --- |
|  | **n** | **strongly agree or agree (%)** | **n** | **strongly agree or agree (%)** |
| Satisfied about the eHealth app overall | 975 | 78.5 | 224 | 56.1 |
| Enhances experience of health services | 962 | 77.5 | 262 | 65.7 |
| Enhances concerns about health information | 926 | 74.6 | 264 | 66.2 |
| Enhances management of health by one’s own self | 889 | 71.6 | 247 | 61.9 |
| Improves health of family members | 663 | 53.4 | 211 | 52.9 |

|  | **Downloaded and used eHealth app (n=1242)** | | | **Downloaded but not used eHealth app (n=399)** | | |
| --- | --- | --- | --- | --- | --- | --- |
|  | **n** | **Mean (SD)** | **95% CI** | **n** | **Mean (SD)** | **95% CI** |
| Satisfied about the eHealth app overall | 975 | 3.86 (0.74) | 3.81-3.90 | 224 | 3.43 (0.88) | 3.34-3.52 |
| Enhances experience of health services | 962 | 3.88 (0.74) | 3.84-3.92 | 262 | 3.64 (0.8) | 3.56-3.72 |
| Enhances concerns about health information | 926 | 3.81 (0.74) | 3.76-3.85 | 264 | 3.62 (0.8) | 3.55-3.70 |
| Enhances management of health by one’s own self | 889 | 3.79 (0.76) | 3.74-3.83 | 247 | 3.58 (0.79) | 3.50-3.66 |
| Improves health of family members | 663 | 3.52 (0.81) | 3.48-3.57 | 211 | 3.46 (0.83) | 3.38-3.54 |
