# Supplementary material for: Factors Associated With the Acceptance of an eHealth App for Electronic Health Record Sharing System: Population-Based Study
Source: J Med Internet Res. 2022 Dec 12;24(12):e40370. doi: 10.2196/40370 (PMC9793296; doi:10.2196/40370)
Supplement: Multimedia Appendix 10 [file jmir_v24i12e40370_app10.docx]

|  | **Downloaded and used eHealth app**  **(n=1242)** | | | **Downloaded but not used eHealth app**  **(n=399)** | | **Not having downloaded and used eHealth app**  **(n=469)** | | |
| --- | --- | --- | --- | --- | --- | --- | --- | --- |
|  | **n** | **yes (%)** | **n** | | **yes (%)** | **n** | **yes (%)** |  |
| Will download, adopt, or continuously adopt in the future | 1105 | 89.0 | 283 | | 70.9 | 320 | 68.2 |  |
| Will recommend friends, colleagues and family members to download | 1024 | 83.2 | 291 | | 72.9 | 304 | 64.8 |  |
| Include the lab results | 1094 | 88.1 | 293 | | 73.4 | 320 | 68.2 |  |
| Include the radiographic images (e.g. X Ray, CT Scan, MRI) | 980 | 78.9 | 243 | | 60.9 | 261 | 55.7 |  |
| Include information on community health activities | 488 | 39.3 | 140 | | 35.1 | 169 | 36.0 |  |
| Collaboration and interface with other software | 567 | 45.7 | 167 | | 41.9 | 208 | 44.3 |  |
| Tailored health tips | 668 | 53.8 | 206 | | 51.6 | 247 | 52.7 |  |
| Provide age-specific healthcare recommendations | 843 | 67.9 | 244 | | 61.2 | 291 | 62.0 |  |
| Include functions of other health applications (e.g. sleep monitoring, exercise records) | 489 | 39.4 | 136 | | 34.1 | 157 | 33.5 |  |
| Include animations | 166 | 13.4 | 54 | | 13.5 | 65 | 13.9 |  |
| Provide voice navigation | 290 | 23.3 | 94 | | 23.6 | 98 | 20.9 |  |
| Offer app personalization (i.e. able to change the font, color etc.) | 270 | 21.7 | 55 | | 13.8 | 81 | 17.3 |  |
| Others | 150 | 12.1 | 43 | | 10.8 | 21 | 4.5 |  |

|  | **Downloaded and used eHealth app**  **(n=1242)** | | | **Downloaded but not used eHealth app**  **(n=399)** | | | **Not having downloaded and used eHealth app**  **(n=469)** | | |
| --- | --- | --- | --- | --- | --- | --- | --- | --- | --- |
|  | **n** | **Mean (SD)** | **95% CI** | **n** | **Mean (SD)** | **95% CI** | **n** | **Mean (SD)** | **95% CI** |
| Will download, adopt, or continuously adopt in the future | 1105 | 4.52 (0.73) | 4.48-4.56 | 283 | 4.09 (0.89) | 4.00-4.18 | 320 | 3.90 (0.88) | 3.82-3.98 |
| Will recommend friends, colleagues and family members to download | 1024 | 4.26 (0.82) | 4.21-4.30 | 291 | 3.96 (0.93) | 3.87-4.05 | 304 | 3.76 (0.90) | 3.68-3.84 |
